# In Situ Quantization with Memory‐Transistor Transfer Unit Based on Electrochemical Random‐Access Memory for Edge Applications

**DOI:** 10.1002/advs.202521815

**Published:** 2026-01-21

**Authors:** Zhen Yang, Yuxiang Yang, Baiqian Wang, Yaoyu Tao, Zelun Pan, Lei Cai, Teng Zhang, Longhao Yan, Xianbin Li, Yuchao Yang

**Affiliations:** ^1^ New Cornerstone Science Laboratory Beijing Advanced Innovation Center for Integrated Circuits School of Integrated Circuits Peking University Beijing China; ^2^ State Key Laboratory of Integrated Optoelectronics College of Electronic Science and Engineering Jilin University Changchun China; ^3^ Center For Brain Inspired Chips Institute For Artificial Intelligence Frontiers Science Center For Nano‐optoelectronics Peking University Beijing China; ^4^ New Cornerstone Science Laboratory Guangdong Provincial Key Laboratory of In‐Memory Computing Chips School of Electronic and Computer Engineering Shenzhen Graduate School Peking University Shenzhen China; ^5^ Center For Brain Inspired Intelligence Chinese Institute for Brain Research (CIBR) Beijing China

**Keywords:** ECRAM, edge computing, in‐memory computing, weight quantization

## Abstract

In‐memory computing based on nonvolatile synaptic arrays with computing functions has significantly improved the computing energy efficiency of neural networks. However, current synaptic devices are mostly limited to accelerating matrix‐vector multiplication operators, and the differentiated requirements for device characteristics in the training/inference stage have led to a sharp increase in the integration complexity of hybrid synaptic units. Hence, for low‐precision quantization calculations of networks, a compact synaptic unit based on ionic nonvolatile memory‐transistor coupling integration, which enables in situ approximate weight quantization without additional binary programming while maintaining parallel MVM computing capabilities, is developed. Results show that the quantization function, derived from the cell's physical electrical properties, achieves classification accuracy in binary neural networks comparable to the ideal quantization function. This approach supports low‐precision continual learning, mitigates catastrophic forgetting, and enables efficient computations for binary/ternary large language models. At a 4 Mb array scale, ECRAM‐ and RRAM‐based units achieve energy consumption advantage of 25.51× and 4.84×, respectively, over traditional digital platforms, offering a robust in situ quantization framework for low‐precision edge training.

## Graphical abstract

By combining ionic nonvolatile memories and transistors, this work proposes a compact synaptic unit to enable low‐precision neural network training. The design supports in situ weight quantization without extra programming and achieves accuracy comparable to ideal methods. This work obtains energy consumption advantage of 25.51× (ECRAM) and 4.84× (RRAM) over digital platforms, showing the potential for efficient edge AI and large language model computations.

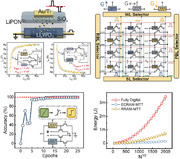

## 1 Introduction

Recently, with rapid progress in deep learning (DL), a range of complicated tasks have been incredibly resolved, such as image classification [1, 2, 3], natural language processing [4, 5, 6], speech recognition [7, 8], and autonomous decision‐making [9, 10, 11]. By continually increasing the depth and size of the deep neural network (DNN) models, DL will even outperform the human's level in some cognitive tasks [3, 12]. However, exponentially growing network parameters impose unsustainable hardware resource and power demands, particularly for edge‐based data‐intensive applications [13, 14, 15]. In conventional computing systems, frequent data transfers between memory and processing units further degrade energy efficiency, posing a critical bottleneck for scaling future models [16, 17].

**FIGURE 1:**
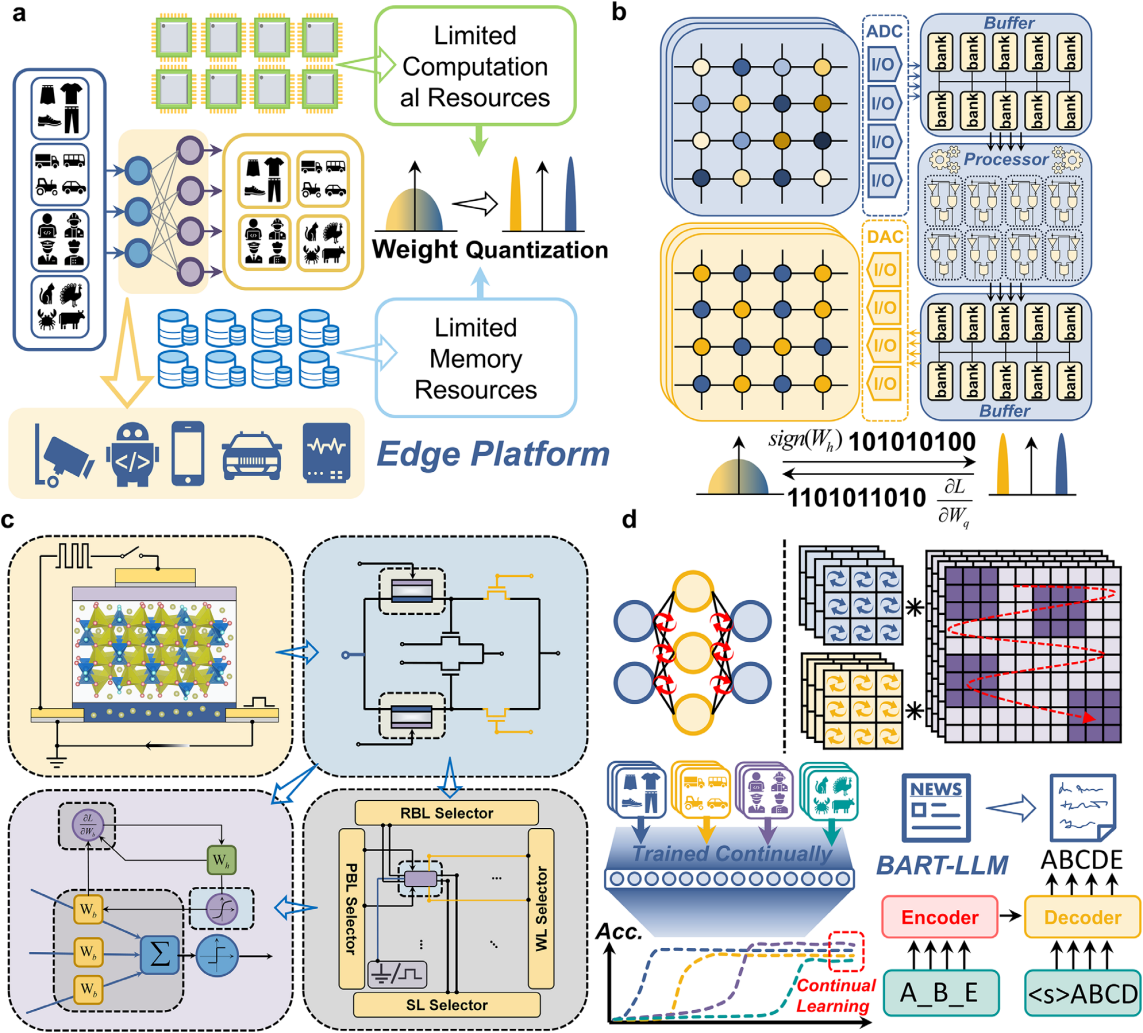
In situ quantization for edge computing based on MTT unit. a) A schematic diagram of the weight quantization of neural networks deployed at the edge, aimed at accommodating limited computational and storage resources. b) During the network quantization‐aware training process, data needs to be transferred between two weight storage arrays with different precision frequently, where the access reads, quantization computations, and low‐precision programming contribute significantly to the additional latency and energy consumption. c) The context of this article is based on novel non‐volatile memory devices leading to the proposed memory‐transistor transfer unit, as well as the array operations and reading methods based on this unit structure, including the final application of network algorithms. d) The relevant applications addressed in this paper include lightweight network models for edge deployment, metaplasticity‐inspired continual learning models, and large language models based on low‐precision quantization.

To address the trade‐off between massive parameters and limited computing power, brain‐inspired computing‐in‐memory (CIM) architectures leveraging emerging nonvolatile memory (eNVM) devices have been proposed to improve energy efficiency in DL applications [18]. By integrating these eNVM devices into the crossbar arrays, matrix‐vector multiplication (MVM) based on electrical principles can be physically in situ conducted without frequent data movements, which has been broadly utilized to accelerate the neural network inferences by mapping the weights into device conductance [19, 20, 21]. However, modern high‐performing DNN models often rely on high‐precision parameters (e.g., 32‐bit floating‐point), demanding complex computations and large memory capacity [22]. For most eNVM devices, such as resistive random‐access memory (RRAM) [23, 24, 25, 26], phase‐change memory (PCM) [27, 28, 29], magnetoresistive random‐access memory (MRAM) [30, 31], and ferroelectric tunnel junction (FTJ) [32, 33, 34], the limited number of conductance states require adjusted weight quantization. Although some researchers have proposed more advanced programming method to significantly enhance storage state numbers in single devices [21, 35], low‐bit weight quantization in hardware employment is still necessary. As demonstrated in Figure 1a, for the limited computational and memory resources in edge platforms, such as mobile phones and embedded devices, neural network quantization, in which original floating‐point weights are represented with highly low precision while keeping performance degradation extremely low, has been utilized to compress the model size [36]. By quantizing the neural network weights into 1‐bit precision, which is also termed as binarization, a recent study replaced the heavy MVM operations well with bitwise XNOR operations and bitcount operations, and realized up to 32× memory saving and 58× speedup on CPUs [37].

As shown in Figure 1b, the integration of CIM architecture with hardware‐friendly binary neural networks (BNNs) utilizes two distinct arrays to store different precision weights: real‐valued analog and quantized binary weights [36]. For edge applications in adapting to complex environments, frequent neural network finetuning is necessary, which requires lots of quantization operations during online training. This process demands simultaneous storage of both weight types due to quantization's irreversible nature. However, hardware quantization still involves abundant data transfer between analog memory arrays and digital processing units, as well as between digital units and binary programming arrays, incurring additional energy and latency overheads [38]. Although in situ MVM has been realized by adopting CIM architecture, plenty of weight quantization computation and low‐precision programming operations, which dominate in neural network quantization algorithms, are still performed separately due to the lack of in situ physical operators of quantization function in the CIM paradigm [39].

Many works about on‐chip implementation of BNN focus on the inference stage, by mapping the trained weights into CIM arrays, but few take account of enhancing low‐precision neural network training efficiency [36, 37]. For the hardware implementation of training low‐precision neural networks, such as BNN, D. Shang et al. proposed a hybrid analog‐digital hardware system equipped with an RRAM chip to deploy a mixed‐precision continual learning (MPCL) model [40]. In their MPCL hardware employment, binary weights are represented by the normalized conductance of RRAM differential pairs while high‐precision floating‐point weights and related operations are carried out on the general digital processor. Through in situ MAC accelerations in the RRAM CIM arrays, about 200× energy consumption has been reduced during the inference phase compared with the conventional digital systems, but separate storage of different‐precision weights still limits their online training efficiency, and the RRAM‐digital mixed storing methods are broadly adopted in other works about BNN online training [36, 41]. To solve the separated weight transfer issues, Martemucci et al. proposed a hybrid FeRAM/RRAM synaptic circuit to enable on‐chip inference and learning of BNN, utilizing the excellent writing characteristics of FeRAMs and the nondisruptive reading capability of RRAMs [38]. Nevertheless, extra binary programming and low‐precision reading for performing ex situ inference are still needed, and the large footprint of FeRAM consumes significant chip area.

Electrochemical random‐access memory (ECRAM), a three‐terminal eNVM with metal‐oxide‐semiconductor field‐effect transistor (MOSFET)‐like structure, has emerged as a promising synaptic device for artificial neural network (ANN) accelerators [42, 43, 44, 45, 46, 47], whose conductance can be accurately regulated through electrochemical ionic doping and dedoping processes. In contrast to conventional two‐terminal memristive devices, ECRAM enables multi‐state programming in a more linear and symmetrical manner, accompanied by low variability and highly deterministic tuning characteristics. Owing to its nearly ideal conductance programming attributes, ECRAM exhibits significant potential in addressing the challenges associated with on‐chip training of ANNs.

In this study, we aim to address the inefficiency of separate data handling in low‐precision DNN quantization within conventional CIM architectures. Based on the superior linear and stable programming characteristics of WO_x_‐ECRAM, we develop an analog‐binary weight transfer unit called memory‐transistor transfer (MTT) unit, enabling in‐situ approximate quantization without extra binary programming while preserving parallel MVM capability in CIM‐like arrays. The weight transfer functions are also systematically explored under the impacts of different bias conditions. Furthermore, compatible programming and inference schemes of MTT‐based arrays were developed and experimentally demonstrated to verify the availability. The neural network testing results reveal that the quantization function derived from the inherent physical electrical properties of the proposed cell architecture maintains classification accuracy in binary neural networks on par with the ideal function. This approach facilitates low‐precision continual learning model computations, effectively mitigating the catastrophic forgetting problem inherent in conventional neural networks and enabling efficient computations for binary/ternary large language models (LLMs). At a 4 Mb array scale, the ECRAM‐based and RRAM‐based quantization units exhibit significant enhancements in energy consumption, achieving improvements of 25.51× and 4.84×, respectively, over traditional digital platforms in quantization‐related computation and storage. This advancement offers a highly energy‐efficient in situ quantization computing framework for low‐precision network training in edge computing environments.

## 2 Results and Discussion

### 2.1 Characteristics of ECRAM Used in MTT Units

**FIGURE 2:**
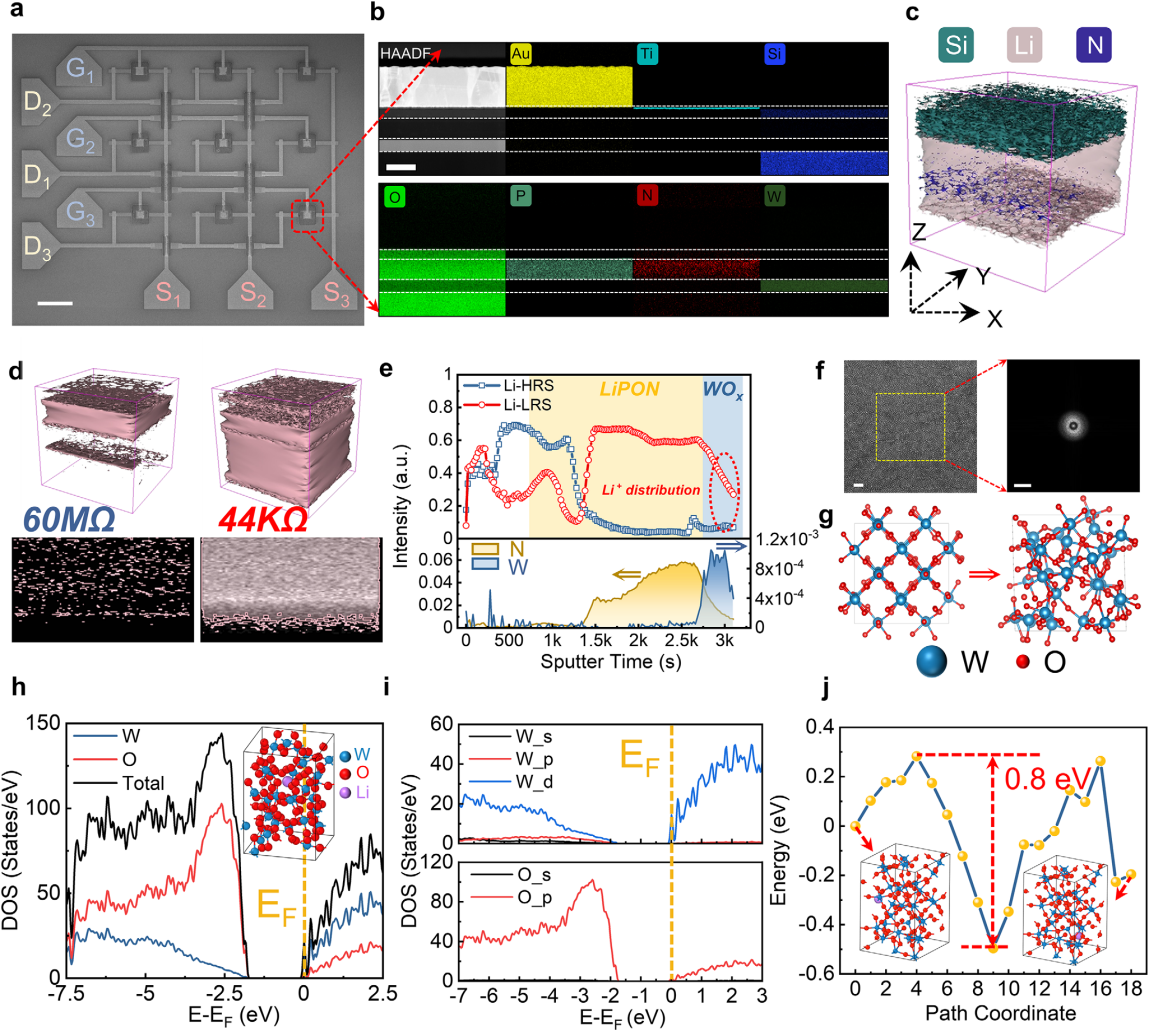
Characterization of ionic regulation mechanisms within ECRAM. a) SEM image of the ECRAM array, which contains nine independent ECRAM devices that can be programmed and read by different gate, drain, and source lines. Scale bar, 50 µm. b) EDS distribution map of the layers in the core region of the ECRAM device. Scale bar, 100 nm. c) 3D SIMS distribution for Si, Li, and N elements when Li ions were intercalated into a channel layer. d) Comparison of Li spatial distribution for ECRAM at different states. e) SIMS depth profiles for the ECRAM in low‐resistance and high‐resistance states, where the yellow and blue backgrounds represent the LiPON and WO_x_ layers, respectively. f) HRTEM image of the WO_x_ layer and the corresponding diffraction pattern extracted by fast Fourier transformation for the yellow rectangular regions, the scale bar for the two images are 2 nm and 10 nm^−1^, respectively. g) The transformation from crystalline structure to amorphous structure of WO_x_ by using molecular dynamics. h) The density of states (DOS) of amorphous WO_x_ after Li element doping. The Fermi level is set to zero. i) Projected DOS of W and O elements in panel (h). j) Energy changes of Li ions during migration.

High‐performance memory devices with minimal programming stochasticity and high endurance are essential for reliable weight conversion in MTT units. In this work, we use ECRAM as hidden weights, where conductance modulation enables indirect control of the inference transistor's linear region resistance, facilitating weight conversion and in situ approximate quantization. Figure 2a shows the fabricated ECRAM array (fabrication details in Figure S1 and the Experimental Section). An additional SiO_2_ layer is employed to isolate the gate line from the drain line, preventing short‐circuiting between the two. The device structure (Figure 2b) features: (1) a WO_x_ channel with tunable band structure via ionic doping, (2) lithium phosphorus oxynitride (LiPON) solid electrolyte, in which abundant moveable Li ions can be controlled by the gate voltage, and (3) an SiO_x_ passivation layer fabricated along with LiPON protecting LiPON from ambient degradation (elemental analysis in Figure S2a,b). Time‐of‐flight secondary ion mass spectrometry (TOF‐SIMS) analysis is utilized to better reveal the spatial distribution of Li ions (see the Experimental Section for experimental details). As shown in Figure 2c, the three‐dimensional (3D) spatial distribution of Si, Li, and N elements can be well visualized, and a large number of mobile Li ions are the basis of ECRAM programming. A more intuitive comparison of Li element distribution within ECRAM of different conductance states can be seen in Figure 2d, showing the Li ions are more concentrated near the channel region when the ECRAM is at higher conductance states. The SIMS depth profile along the gate stack is shown in Figure 2e accordingly, where many Li ions will be driven to the WO_x_‐based channel and results in the depletion at the upper region of the LiPON electrolyte.

To reveal the ionic modulation mechanisms in the ECRAM programming more clearly, the first‐principles calculations are performed based on the characterization data. Different from the previously used crystalline material in the ECRAM channel [44, 48], the WO_x_‐based channel is produced by the reactive sputtering without subsequent annealing to simplify the process flow and reduce the thermal budget, so its amorphous structure will be kept, verified by Figure 2f and Figure S2c,d. Therefore, we first generate an amorphous WO_x_ cell model through the rapid quenched molecular dynamics, with construction process depicted in Figure S3a. The process mainly includes four stages, that is initialization, melting‐diffusion, quenching and relax, and the changes in the final microstructure are compared in Figure S3b. The WO_x_ structure (Figure 2g) is obtained to conduct the following electronic structure calculations, whose isotropy can help improve uniformity during ionic doping. Then, the density of states (DOS) of the Li‐WO_x_ system is calculated, and the results are shown in Figure 2h, in which the Fermi Level is located at the conduction band. These results, compared with Figure S4a, show that Li ions lead to n‐type doping in WO_x_. The projected DOS in Figure 2i reveals that the main contributions to the conduction band are from W_5d_ and O_2p_ electrons. As shown in Figure S4b,c, the DOS with the Li ion at different site are both similar to the results in Figure 2h, which accords the isotropy of the amorphous WO_x_. The migration energy barrier for Li ions in the WO_x_ matrix is shown in Figure 2j, whose maximum barrier is only 0.8 eV, which indicates the Li ions can be easily doped into the WO_x_ without excessively high gate voltage, which is critical for low supply voltage applications.

**FIGURE 3:**
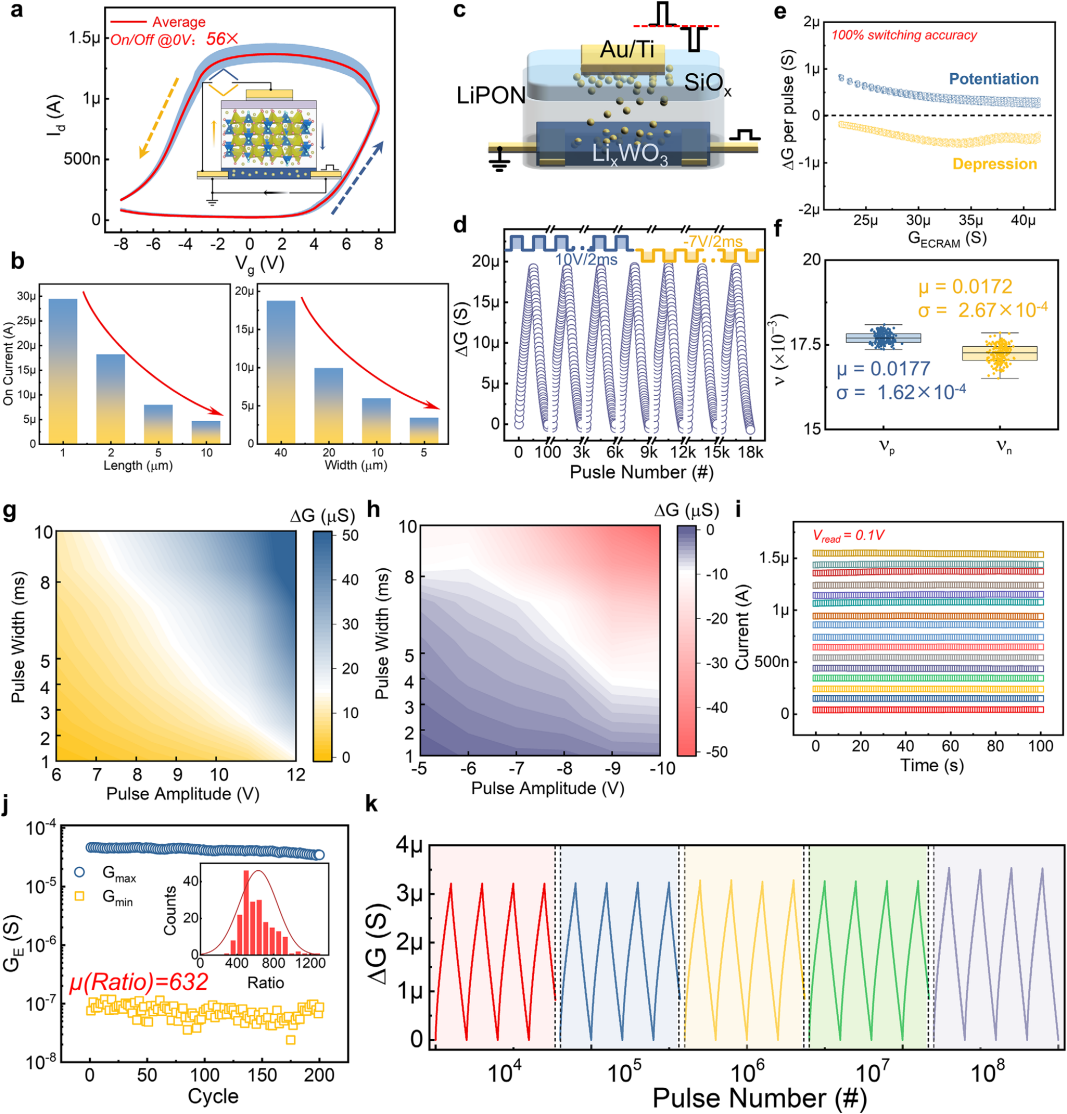
Characterization of the basic properties of ECRAM. a) Transfer characteristics of the ECRAM under 50 continuous cycles, with a maximum scanning voltage amplitude of 8 V for both positive and negative regions, yielding an average switching ratio of 56 at a gate voltage of 0 V. b) The on‐state current of ECRAM in different sizes, including different lengths and widths. c) Schematic diagram of the basic operations of the ECRAM device, illustrating the doping and de‐doping of the channel by mobile ions in the electrolyte, controlled by the electric field applied between the gate and the channel. d) Variation of long‐term potentiation and depression during 180 continuous cycles, where the potentiation and depression pulse amplitudes are 10 and −7 V, respectively, each with a width of 2 ms. e) Statistics of conductance variation for each programming pulse during potentiation and depression. f) The distribution of fitting parameters of programming linearity for both potentiation and depression. g‐h) Statistical data on the potentiation and depression of ECRAM conductance under different programming pulse amplitudes and widths. i) Retention characteristics of 16 different states of the ECRAM, with a channel read voltage of 0.1 V. j) Statistical data on the high switching window of the device during continuous conductance modulation, achieving an average ratio of 632. k) Endurance test for over 10^8^ programming pulses, showing no device degradation during these pulse stimuli, with specific details demonstrating the conductance modulation characteristics of the device after 10^4^, 10^5^, 10^6^, 10^7^, and 10^8^ pulses.

Under the guidance of the electrochemical ion doping mechanism, the channel conductance can be well controlled by the gate voltages, where doping and dedoping are realized by the direction of the electric field. The basic transfer characteristics of the ECRAM under 50 continuous cycles are illustrated in Figure 3a, with switching principle shown in the inset. The channel conductance increases under positive sweeping, while it decreases at negative sweeping region, and an average switching ratio with gate voltage at 0 V reaches 56. Besides WO_x_, amorphous NbO_x_ can also be utilized [49, 50] as ECRAM channel for its outstanding stability and electronic tunability. However, for the more stable nonvolatility in serving as hidden weights in MTT units, we finally choose WO_x_ as the ECRAM channel material. The relationship between the formation energy of interstitial Li defects and Fermi level for NbO_x_ and WO_x_ systems are compared in Figure S5, and the lower overall formation energy in WO_x_ helps improve the stability of doped Li ions.

Furthermore, the size effects on the characteristics of ECRAM are explored in Figure 3b and Figure S6. The device on‐state current can be enhanced by widening or shortening core areas, owing to the uniform ionic doping over the channel region, and the overall conductance *G_on_* can be well expressed by:

(1) $$\begin{array}{c} \;G_{on}=g\frac{W}{L} \end{array}$$

where *g* is proportional to the on‐state conductivity under the ionic doping, which is almost the same for devices of different sizes, and *W* and *L* denote the width and length of device core region, respectively. This provides guidance for choosing proper device sizes under the maximum on‐state current requirement. In addition, as shown in Figure S7, the switching window can be enhanced either by increasing the sweeping intervals or by increasing the sweeping amplitudes, indicating that the electrochemical doping is a gradual process in which ion migration is mainly driven by electric field.

Pulse‐based programming, whose basic operation is diagrammatically depicted in Figure 3c, with source grounded and drain monitored by 0.1 V bias voltage, positive and negative programming pulses are implemented at the gate, which corresponds to potentiation and depression of ECRAM, respectively. The typical 180‐cycle long‐term potentiation and depression programming is demonstrated in Figure 3d, in which 10 V/2 ms and −7 V/2 ms pulses are used to write and erase, respectively. Different from the common programming stochasticity in two‐terminal non‐volatile memory devices, especially for filament‐based RRAM, 100% switching accuracy can be ensured in ECRAM, in which the conductance changes during each pulse are shown in Figure 3e. The high switching accuracy helps improve the programming efficiency, which is critical to the edge applications. In addition, by adopting the fitting model from the work of Chen et al. [51], the statistics of linearity in the conductance updating are concluded in Figure 3f, demonstrating excellent uniformity during the 180‐cycle updating. More systematic results of programming sensitivity under different amplitudes and widths of the programming pulse are illustrated in Figure 3g,h, and we developed a write‐verify scheme based on the increasing pulse amplitude accordingly to accelerate conductance convergence, whose flowchart is shown in Figure S8. In addition to the device programming in training, the retention of different states is important in inference, with the results shown in Figure 3i. It should be noted that the nonvolatility of the hidden weights also ensures that of the inference weights in the proposed MTT units. As shown in Figure 3j, the ECRAM reached a high switching ratio of 632 with updating process and applied pulses in Figure S8, which enables binary quantization fitting in the MTT unit. Another key characteristic of the memory device used in online training is ultrahigh endurance. Thanks to the reversible dynamic ionic doping and dedoping, the damage to the lattice structure can be negligible during repeated programming, and more than 10^8^ pulse endurance is shown in Figure 3k. Detailed programming by µs‐level pulse stimulation can be found in Figure S9a, and the evolution of maximum conductance changes and linearity fitting parameters during the whole programming in Figure 3k are demonstrated in Figure S9b,c, respectively. In addition, conductance updating processes in the endurance tests for the first 16×10^6^ pulses and the last 16×10^6^ pulses are compared in Figure S10, indicating that the ECRAM can keep excellent performances during the endurance tests. The above characteristics of the ECRAM ensure this kind of analog memory device is highly appropriate to serve as hidden weights in the following discussed MTT units.

### 2.2 Weight Transfer Functions in MTT Units

The excellent analog programming characteristics discussed above ensure highly efficient weight mapping in the MTT units. As the theoretically derived model in Figure S11a, the symmetrical structure is adopted to form a differential weight unit. It should be noted that reference resistors denoted by yellow rectangles with conductance *G_Ref_* is used to simplify model derivation, though they are implemented by transistors with fixed gate voltage in actual circuits. We consider the resistance value *R_Ref_* of the reference resistors to be 1. The purple variable resistor represents the analog memory device, and the transistors whose gates are controlled by the voltage division between the variable resistors and fixed resistors provide the binary inference weights, realizing the purpose of in situ weight transfer.

Considering *V_T_* to be the threshold voltage of the transistors used in MTT units and their transfer characteristic described in the Experimental Section, the hidden weight reading bias voltage $V_{read\_H}$ is set as 2 × *V_T_*. The reading bias voltages applied at the drains of the inference transistors, whose conductances are denoted by $G_{B\_+}$ and $G_{B\_-}$, are 0.1 and −0.1 V, respectively. Thus, the final binary inference weight *G_B_* is the difference between the two, that is:

(2) $$\begin{array}{c} \;G_{B}=G_{B_{+}}-\;G_{B_{-}} \end{array}$$

For the mapping and programming of the analog hidden weight *W_h_*, when *W_h_* ≥ 0, negative analog memory device $G_{A_{-}-}$ will be immediately programmed to *R_Ref_*, then $G_{A_{-}+}$ will be programmed to the corresponding values according to a linear mapping relationship. When *W_h_* < 0, positive analog memory device $G_{A_{-}+}$ will be fixed at value 1, then we will program the negative analog memory device. The final analog conductance *G_A_* can be expressed as $\max\{G_{A\_+},\;G_{A\_-}\}$, and the quantized hidden conductance *G_H_* can be written as:

(3) $$\begin{array}{c} \;G_{H}=\left\{\begin{array}{c} \frac{G_{A}-G_{Ref}}{K\times G_{Ref}},\;\;W_{h}\geq 0\\ \frac{-G_{A}+G_{Ref}}{K\times G_{Ref}},\;\;W_{h}<0 \end{array}\right. \end{array}$$

Here, *K* is a scaling factor constraining *G_H_* to [−1, 1], effectively representing the dynamic range (on/off ratio) of the analog memory device. Due to the MTT unit's symmetrical design, Figure S11b shows how the binary inference weight *G_B_* relates to the quantized hidden conductance *G_H_* for varying *K*. Larger *K* values make this relationship approximate an ideal binary quantization function. The electrical behavior of the devices translates this to a functional mapping between binary inference weight *W_b_* and analog hidden weight *W_h_* as follows:

(4) $$W_{b}=\left\{\begin{array}{c} 2\times \left(\frac{K\times W_{h}+1}{K\times W_{h}+2}-0.5\right),\;\;W_{h}\geq 0\\ -2\times \left(\frac{-K\times W_{h}+1}{-K\times W_{h}+2}-0.5\right),\;W_{h}<0 \end{array}\right.$$

By varying *K*, the weight transfer functions are shown in Figure S11c. As before, larger *K* values make the function approach an ideal Sign function for binarization. However, practical device programming faces trade‐offs: excessive dynamic range degrades device performance, necessitating a balanced operating range that preserves quantization accuracy. To evaluate this, we test different *K* values in a continual learning neural network model [52] inspired by synaptic metaplasticity (simulation details are described in the Experimental Section). Results (Figure S11d–f) show that continual learning performance saturates at *K* ≥ 20, suggesting this as the optimal lower bound for subsequent weight transfer studies.

When the hidden weight reading bias voltage $V_{read\_H}$ is below 2 × *V_T_*, the inference transistor operates in the subthreshold region at low analog memory conductance. As simulated in Figure S12a, this creates a zero‐gradient interval (with half its length defined as the radius). Figure S12b shows the abstracted weight transfer function under different radii, while Figure S12c–e presents the corresponding network performance. Under lower $V_{read\_H}$, larger radii exacerbate deviations from the ideal Sign function, degrading performance, which likely results from constrained hidden weight updates in the subthreshold regime. Thus, $V_{read\_H}$ should exceed twice *V_T_* to ensure robust binary quantization.

The MTT unit can alternatively employ an asymmetric differential structure (Figure S13a). A potential advantage of this configuration is the consistency of the two analog hidden weights during weight mapping. The quantized hidden conductance *G_H_* is then determined by the analog conductance *G_A_* through the following relationship:

(5) $$G_{H}=\frac{G_{A}-G_{Ref}}{K\times G_{Ref}}$$

where all the parameters are the same as before. With the hidden weight reading bias voltage set to 2 × *V_T_*, electrical characterization (Figure S13b) reveals the asymmetric weight transfer function between binary inference weight *G_B_* and quantized hidden conductance *G_H_* across different on/off ratios. The derived mathematical relationship is:

(6) $$\begin{array}{c} \;W_{b}=\frac{K\times W_{h}}{K\times W_{h}+2\times G_{ref}} \end{array}$$

where we set *G_ref_* at 50 for convenience, and the specific function graph is shown in Figure S13c. The asymmetry between positive and negative weight transfer functions intensifies with larger operating ranges, even saturating even when *W_h_* gets close to 1, which results in significant deviation from the ideal Sign function. Network‐level performance tests (Figure S13d–f) confirm this limitation. While the asymmetric MTT unit eliminates hidden weight programming identification, its poor binary quantization fidelity renders it unsuitable for in‐situ weight transfer applications.

**FIGURE 4:**
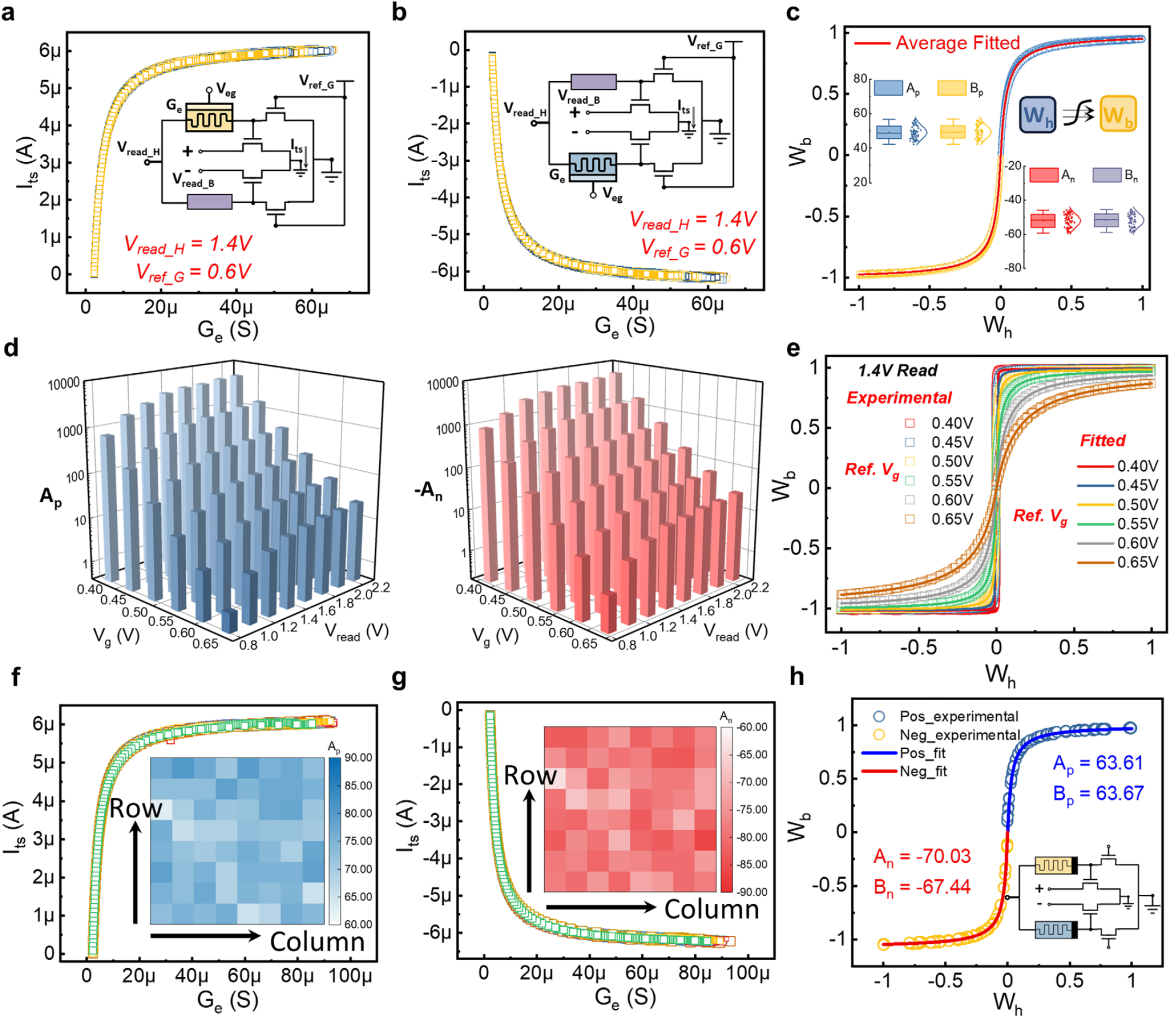
The performances under different biases for MTT units. a,b) The relationship between ECRAM conductance and the channel current of the inference transistor under positive and negative bias conditions, respectively. The hidden weight bias voltage is set at 1.4 V, with the gate voltage of the reference transistor set at 0.6 V. The positive and negative read voltages at the drain of the inferred transistor are set at 0.01 and −0.01 V, respectively. c) The relationship between the quantized hidden weights and the binary weights corresponding to (a) and (b), with the inset showing the distribution of the fitted parameters under different cycle stimuli. d) Parameter fitting results for the weight transfer function of the MTT unit at different hidden weight bias voltages and gate voltages of the reference transistor. e) Relationships between the quantized hidden weights and the binary weights in the MTT unit, with the hidden weight bias voltage fixed at 1.4 V and varying gate bias voltages of the reference transistor. f,g) Statistical data on the positive and negative relationships between the conductance of ECRAM and the channel current of the inference transistor among the 64 different MTT units, with the inset displaying the distribution of the corresponding fitted parameters. h) The relationship between the quantized hidden weights and binary weights in the RRAM‐based MTT unit, with the fitted curve indicated by the solid line in the figure.

In experimental implementation, we replace the reference element with a transistor biased at constant gate voltage ($V_{ref\_G}$ = 0.6 V) for CMOS compatibility and operational flexibility, while also improving the weight transfer function's dynamic range. Figure 4a,b shows the measured relationships between ECRAM conductance *G_e_* and inference transistor current *I_ts_* at $V_{read\_H}$ = 1.4 V, with experimental setups illustrated in insets. Figure 4c presents the multi‐cycle binary weight *W_b_* versus quantized hidden weight *W_h_* relationship, with quantization details in the Experimental Section and parameter distributions shown in the inset. This leads to the generalized model:

(7) $$\begin{array}{c} \;W_{b}=\left\{\begin{array}{c} 2\times \left(\frac{A_{p}\times W_{h}+1}{B_{p}\times W_{h}+2}-0.5\right),\;\;W_{h}\geq 0\\ -2\times \left(\frac{A_{n}\times W_{h}+1}{B_{n}\times W_{h}+2}-0.5\right),\;\;W_{h}<0 \end{array}\right. \end{array}$$

where *A_p_*, *B_p_*, *A_n_*, and *B_n_* parameters take the similar role of *K*, indicating the effective operating dynamic range for the memory device. Figure S14a–c shows the transistor characteristics, where linear region operation is crucial for analog processing since inference transistor conductance is not purely binary. To systematically study the weight transfer function, we varied $V_{ref\_G}$ and $V_{ref\_G}$ while fixing maximum device conductance at 40 µS. The resulting fitting parameters (Figure 4d, Figure S15) reveal consistent trends: lower $V_{ref\_G}$ and higher $V_{read\_H}$ produce better approximation of ideal binary quantization. Notably, replacing the reference transistor with a fixed resistor (Figure S16) yields transfer functions insensitive to bias voltages, with nearly identical fitting parameters across all tested dynamic ranges (Figure S16f). Figure S14d,e shows enhanced scaling factors in the reference‐transistor‐based MTT unit, particularly at high $V_{read\_H}$ The nonlinear channel resistance increase with drain‐source bias (Figure S14c) accelerates node voltage rise, causing faster transfer function saturation as |*W_h_* | approaches 1. This nonlinearity effectively expands the dynamic range, reflected in the fitting parameters. Parameter pair consistency (*A_p_*, *B_p_*) and (*A_n_*, *B_n_*) in Figure S14f ensures transfer symmetry. Reducing $V_{ref\_G}$ lowers overall channel conductance, including the minimum memory device conductance (equal to reference transistor conductance at $\frac{V_{read\_H}}{2}$), thereby enhancing dynamic range under fixed upper limits. Figure S17 shows the ECRAM conductance‐current relationship across $V_{ref\_G}$ values, with corresponding weight transfer functions in Figure 4e showing excellent model agreement. While lower $V_{ref\_G}$ improves Sign function approximation (crucial for BNNs), it demands higher switching ratios, requiring trade‐offs between fitting accuracy and programming efficiency. Uniformity tests across MTT units (Figure 4f,g, Figure S18a,b) demonstrate consistent weight transfer functions, enabling low‐variation array implementation. Notably, Figure S18c,d reveals a near‐linear relationship between transfer parameters and maximum operational range.

While ECRAM serves as an excellent analog hidden weight in the MTT unit due to its superior programming characteristics, the concept can be extended to other nonvolatile memory devices. RRAM, a two‐terminal memory technology, has gained prominence in AI inference chips for its CMOS compatibility, high density, and compute‐in‐memory potential [53, 54]. However, its stochastic and nonlinear programming behavior has limited its adoption in edge training, despite device‐level improvements [55]. Here, we explore RRAM‐based MTT units to broaden hidden memory device options. Figure S19a shows DC switching characteristics of TaO_x_‐based RRAM devices, exhibiting filamentary switching [56]. The RRAM‐MTT unit (Figure 4h inset) uses the 1T1R intermediate node voltage to control the inference transistor, while the reference transistor modulates RRAM analog switching. Multi‐state programmability (16 states, ∼75 on/off ratio) is demonstrated in Figure S19b–e. Despite excellent analog switching, RRAM suffers from nonlinear read behavior (Figure S19f), particularly at high resistance states under large read voltages, which can disrupt stored states. Unlike ECRAM's linear resistance (Figure S20), RRAM requires careful selection of $V_{read\_H}$. For $V_{read\_H}$ = 0.8 V and $V_{ref\_G}$ = 0.7 V, the RRAM–inference transistor current relationship yields a weight transfer function (Figure 4) with fitting parameters >50, meeting transfer requirements. In advanced CMOS nodes with lower threshold voltages, RRAM performs well even with $V_{read\_H}$ at 0.2 V and $V_{ref\_G}$ at 1.2 V, as validated in Figure S21c–e.

### 2.3 Basic Operations for MTT‐Unit‐Based Arrays

In addition to the static weight transfer function of different hidden memory device states, dynamic programming and reading operations are also important in MTT units. To reduce hardware overhead, the reference transistor will be utilized as the selector in array‐level programming. However, for conventional ECRAM‐selector‐based arrays, the selector transistor is mostly connected to the gate terminal of ECRAM [50], which is termed as vertical 1E1T unit (Figure S22a). In this unit, by controlling the switching of the selector transistor, the programming signal can be applied to the ECRAM or not. The selector transistor can also be connected to drain/source terminal of the ECRAM (Figure S22b), and we call this horizontal 1E1T unit (H‐1E1T). A selective programming scheme is proposed based on the H‐1E1T, and the applied signals in each terminal during different programming processes are summarized in Table S2, including potentiation, remaining and depression stages. During all the stages, the V_ED_ terminal and V_ES_ terminal will be kept unchanged, biased at depression voltage V_D_ and grounded, respectively. The electrochemical doping or dedoping within ECRAM is determined by the electric field between gate terminal and drain/source terminal. For potential programming, V_EG_ terminal is applied by the potential voltage V_P_ while V_TG_ terminal is at ground state, and the selector transistor is at Off state; thus, the bias voltage of the ECRAM drain terminal is approximately 0 V, the voltage difference between gate and drain is almost V_P_, which enables conductance to increase. If the selector transistor is at On state, V_TG_ terminal is applied by source supply voltage V_SS_, thus the bias voltage of the ECRAM drain terminal is approximately V_D_, and the effective programming voltage of ECRAM is V_P_ − V_D_, based on previously half‐selected ECRAM programming method [57], the device state can hardly be changed. On the contrary, during the depression stage, the V_EG_ terminal is grounded state and the V_TG_ terminal is applied by source supply voltage V_SS_, then the effective programming voltage of ECRAM is ‐V_D_, and the backward gate‐channel electric field will help drive ions back into electrolyte and enable depressed programming. However, when V_TG_ is also grounded, there will be negligible voltage differences within ECRAM. Compared with the selective programming in vertical 1E1T unit, there is no use of any negative bias signals, reducing the complexity of peripheral CMOS circuits.

**FIGURE 5:**
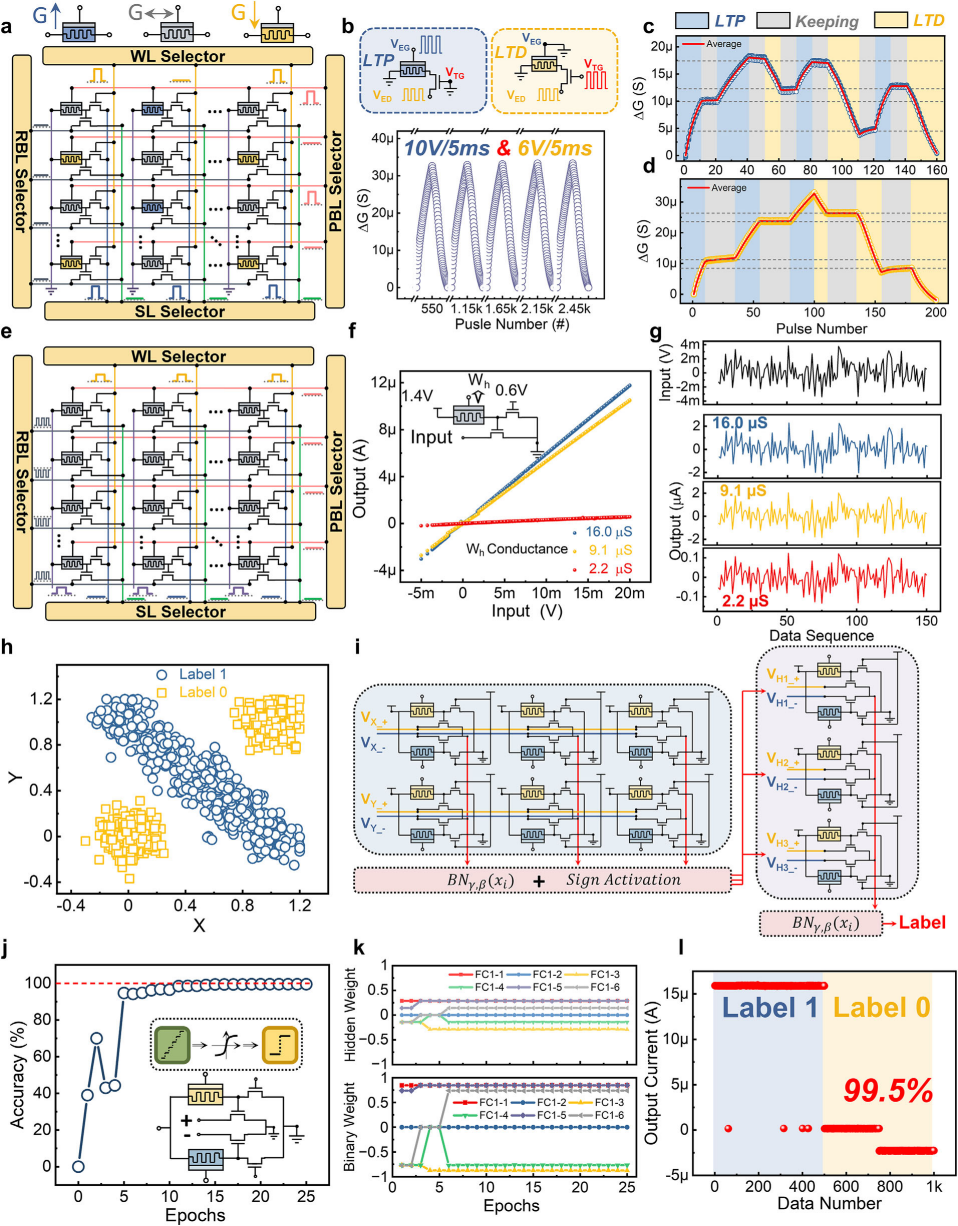
Programming and inference of MTT‐based arrays. a) Schematic of array‐level programming, where programming pulse signals at different ports are labeled alongside the respective signal lines. The blue, gray, and yellow blocks represent increases, maintenance, and decreases in the conductance of the ECRAM after stimulus, respectively. b) Variation of long‐term conductance potentiation and depression in the H‐1E1T unit. The upper panel illustrates the application of pulse signals for conductance potentiation and depression in the H‐1E1T unit, with VEG and VED pulse amplitudes set at 10 and 6 V, respectively, and a pulse width of 5 ms for both. c,d) Selective programming results of the H‐1E1T unit based on the pulse programming scheme shown in panel (a), where the red line indicates the average conductance change during programming. e) Schematic diagram of inference based on the MTT‐unit‐based array, where the inference input waveform is applied at the drain terminal of the inference transistor controlled by the voltage division in the MTT unit. f) Relationship between the simulated input voltage and output current under different hidden weight conductance. g) Specific simulated input voltage waveforms and output current waveforms from panel (f), demonstrate a strong linear relationship that enables analog multiplication computations controlled by hidden weights. h) Linearly nonseparable datasets are used for testing the array composed of MTT units, where shapes and colors represent different classes. i) Schematic representation of a 2×3×1 array composed of MTT units, which also includes batch normalization layers and activation function units based on the Sign function. j) Training performance visualization of the dataset in panel (h) within a BNN based on a 2×3×1 architecture. k) Changes in hidden weights and binary inference weights of the first layer neural network throughout the training process, where the hidden weights are quantized to 3 bits during training. l) Inference results mapped to hardware from the final weights in panel (k), achieving a practical inference accuracy of 99.5%.

The proposed two‐terminal selective programming method enables efficient array‐level parallel operations in our CIM architecture, supporting both row‐wise and column‐wise programming with separate potentiation and depression phases (Figure 5a). Experimental results demonstrate precise conductance control under varying programming voltages (V_P_ and V_D_), showing an exponential relationship between conductance change per pulse and voltage amplitude (Figure S22c,d). Remarkably, this two‐terminal approach maintains excellent programming linearity (Figure S22e), as evidenced by the 100% accuracy achieved in long‐term potentiation/depression cycles (Figure 5b) and its superior linear characteristics (Figure S22f). The system's robustness is further confirmed through continuous selective programming tests across all operational stages, where device states remain stable during retention periods (Figure S23). More complex programming scenarios (Figure 5c,d) successfully validate the architecture's capability for reliable online training applications.

During inference operations, the selector transistors transition into reference transistors with uniformly biased gate terminals, while all ECRAM gates are grounded or left floating to prevent state degradation. Input data is applied to the drain terminals of inference transistors, enabling in situ weight transfer through voltage division between ECRAM devices and reference transistors—a process governed by the previously discussed ECRAM conductance relationship (Figure 5e). This architecture allows dynamic regulation of binary inference weights through modulation of hidden analog memory states (Figure 5f), achieving idealized linear multiplication between input voltages and hidden weights via the inference transistors operating in their linear region. The system's analog computation capability is demonstrated in Figure 5g, showing precise linear transformation of randomly generated input waveforms.

To demonstrate the application of MTT units in mapping neural networks, firstly, a small two‐layer fully connected BNN (2×3×1) is utilized to classify a self‐made XOR dataset (Figure 5h). The hardware architecture based on MTT unit is shown in Figure 5i, which also include batch normalization layers and Sign activation function units, with the data flow indicated by red arrows. Based on the weight transfer function in the MTT unit, the neural network training converges to nearly 100% accuracy within 25 epochs (Figure 5j), and more specific details of the used weight transfer function can be found in the Experimental Section. The evolution of hidden weights and binary inference weights of the first layer in training is compared in Figure 5k, and approximate discrete binarization of analog weights is successfully realized. By mapping the trained hidden weights into ECRAM conductance, the final measured classification accuracy can reach 99.5%, with another similar example (Figure S24) achieving accuracy of 98.5%.

Moreover, for RRAM‐based MTT units, the programming scheme resembles conventional 1T1R. Array‐level programming (Figure S25a) leverages bipolar characteristics: set/reset operations are performed via the PBL and SL selectors, respectively. Programming proceeds row‐by‐row or column‐by‐column, with separate potentiation and depression phases. Inference in RRAM‐MTT arrays mirrors ECRAM‐MTT arrays—an analog input waveform is applied at the drain terminal of the inference transistor, regulated by voltage division in the upper MTT unit (Figure S25b). The two‐terminal compact structure of RRAM‐MTT enables significant area reduction.

With the weight transfer function enabled in both ECRAM‐ and RRAM‐based MTT units, we extend their application to practical binary neural networks (BNNs), which are ideal for edge platforms due to their lightweight nature. Simulations on fully connected (FCNN) and convolutional neural networks (CNN) (see the Experimental Section) show comparable performance between ECRAM‐ and RRAM‐based in‐memory‐binarization (IMB) functions when using idealized Sign binarization (Figure S26). Final testing accuracies across multiple trials are summarized in Table S3. Notably, binary CNN performance improves when employing the physical weight transfer function in MTT units, suggesting that the fuzzy binary quantization can somehow keep more precision at the original hidden weights in CNN and that the physical weight transfer in MTT unit is not just a simple approximation of Sign function.

Prior energy‐efficient computing units often trade functionality for hardware complexity. In contrast, our MTT unit's programming scheme maintains quantization capability while minimizing components. For fair comparison, we evaluate within an in‐memory computing architecture using crossbar‐like arrays. As shown in Figure S27a,c, conventional differential structures require 8 components (4T4E or 4T4R) to store both analog hidden weights and binary weights. However, our MTT unit, optimized for edge quantization networks, achieves this with only 4T2E (Figure S27b) or 4T2R (Figure S27d), enabling simultaneous analog and binary weight storage. Thus, MTT units not only enable in situ weight transfer but also reduce hardware complexity, improving both energy efficiency and footprint.

### 2.4 In Situ Weight Quantization in MTT Units for Continual Learning and LLM

To verify and expand the versatility of the MTT unit in other kinds of important edge applications, two other important applications, continual learning and LLM, are employed to do the examination. [58]

Notably, A. Laborieux's metaplasticity‐inspired BNN [52, 59] demonstrates particular promise for edge implementation, combining synaptic‐centric training with hardware‐friendly characteristics suitable for emerging learning approaches.

Edge applications should adapt to unpredictable real‐world variations, requiring neural networks to continually learn new scenarios without degrading performance on prior tasks, which is a challenge known as catastrophic forgetting. While standard deep learning methods fail to learn incrementally without access to old data, simply retraining on all data (both old and new) is computationally prohibitive for resource‐constrained edge devices. Recent research has developed continual learning algorithms to address this, including regularization, experience replay, dynamic architectures, and knowledge distillation [58]. Among these methods, a metaplasticity‐inspired BNN proposed by A. Laborieux [52, 59], can be generally used in a range of emerging learning approaches. The synaptic‐centric training with hardware‐friendly features make it more suitable for implementation at edge devices.

**FIGURE 6:**
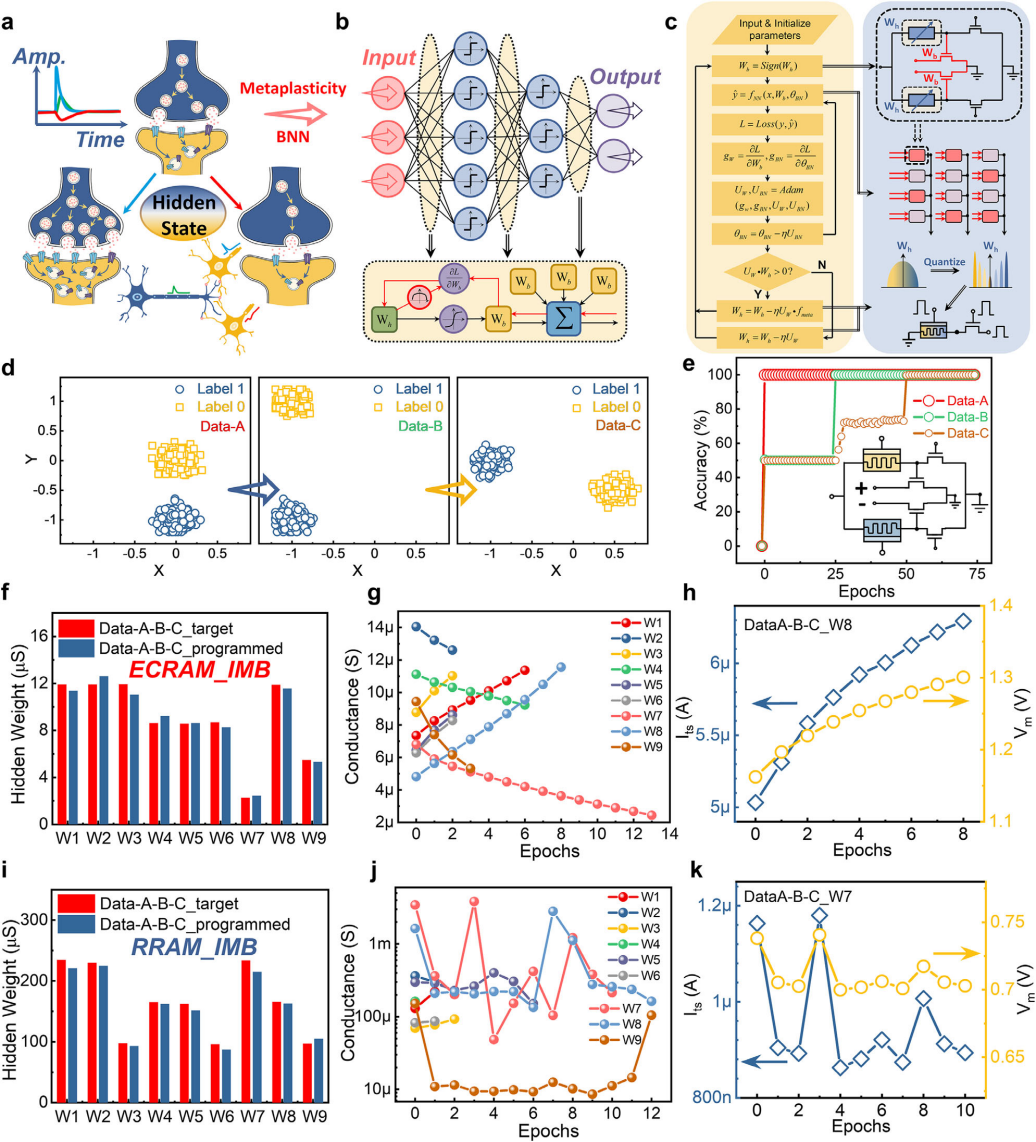
Applications of weight transfer relationships in MTT units for continual learning. a) Schematic diagram of the relationship between synaptic metaplasticity and changes in synaptic weights in biological neural networks. b) Schematic diagram of a continual learning model that combines metaplasticity with binary neural networks, where each weight in the model consists of an analog hidden weight for training and a binary‐value inference weight for inference. During network training, the gradient computation is modulated by the value of hidden weights. c) Algorithm flowchart for the binary neural network based on metaplasticity and its corresponding hardware acceleration, which primarily includes weight binary quantization and in‐memory computation inference components. d) Datasets are used to test the continual learning performance of small networks, comprising three distinct datasets: Data‐A, Data‐B, and Data‐C. e) Training performance of the BNN based on metaplasticity on the different datasets in panel (d), where hidden weights are quantized to 3 bits during training, and the binary quantization curve utilizes weight transfer relationships from ECRAM‐based MTT units. f–h) Statistical distributions of target conductance versus actual conductance for hidden weights in the first layer of the network; the variation of hidden weight conductance during the write‐verify programming process; and the read current of an inference transistor for a typical weight, along with the variation of the intermediate node voltage during the programming process, where weights are implemented using ECRAM‐based MTT units. i–k) Statistical distributions of target conductance versus actual conductance for hidden weights in the first layer of the network; the variation of hidden weight conductance during the programming process; and the read current of an inference transistor for a typical weight, along with the variation of the intermediate node voltage during the programming process, where weights are implemented using RRAM‐based MTT units.

Metaplasticity, inspired by neural mechanisms enabling task retention (Figure 6a), governs synaptic strength changes through hidden states, effectively representing the plasticity of synaptic plasticity. This synaptic consolidation mechanism is crucial for memory storage, where a synapse's metaplastic state quantifies its task importance, preventing catastrophic forgetting. Laborieux et al. [52] incorporated this concept into BNNs by linking real‐valued hidden weights to synaptic importance, enabling continual multi‐task learning. As shown in Figure 6b, their modified BNN training alters gradient computation; that is, when binary inference and hidden weight signs align, the gradient is scaled by a nonlinear meta function *f_meta_*:

(8) $$\begin{array}{c} f_{meta}\;\left(m,W_{h}\right)=1-tanh^{2}\left(m\times W_{h}\right) \end{array}$$

Here, *W_h_* represents the real‐valued hidden weight and m controls the decay rate in non‐zero regions. The gradient computation for binary inference weights *W_b_* incorporates *W_h_* to minimize sign changes, particularly for larger |*W_h_* | values, thereby preserving performance on learned tasks. As shown in left of Figure 6c, conventional CIM architectures face significant overhead from frequent weight binarization between *W_h_* and *W_b_*, as gradient computation and weight quantization occur in separate arrays. This leads to both increased latency/energy from data movement and reduced efficiency from post‐quantization programming. Our solution (Figure 6c, right) integrates MTT units with CIM architecture to eliminate this quantization bottleneck, enabling direct mapping of quantized hidden weights to analog memory devices.

To evaluate continual learning performance, we created three custom datasets (Data‐A/B/C, Figure 6d) and trained a 2×3×1 binary FCNN using the metaplasticity algorithm (see the Experimental Section). ECRAM‐MTT units enabled low‐precision weight quantization. As shown in Figure 6e, the model successfully learned new tasks without catastrophic forgetting. The larger model, which consists of a large number of weight parameters, will be more stable in continual learning for the excess weights to learn new things and keep memory for old things, and the continual learning capacity of larger‐level neural network will be also explored later.

Weight mapping only requires programming analog hidden weights into MTT units, eliminating additional binarization steps. To address device non‐idealities, we employ a write‐verify scheme (Figure S28) with adaptive programming intensity for reliable Set/Reset operations. This universal approach applies to various emerging memories (RRAM, PCM, FeFET, ECRAM, etc.) [18, 19], differing only in specific Set/Reset implementations. For ECRAM‐MTT units, Figure 6f demonstrates precise conductance programming with minimal error, while Figure 6g shows the deterministic switching from random initial states. Figure 6h illustrates the coordinated binary/hidden weight updates in MTT operation. Additional validation (Figure S29a–f) confirms software‐equivalent inference accuracy. RRAM‐MTT implementations (Figure 6i–k, Figure S30) achieve similar accuracy but suffer from nonlinear conductance changes, increasing latency and energy consumption, which highlights the need for RRAM with improved linearity for efficient online training.

**FIGURE 7:**
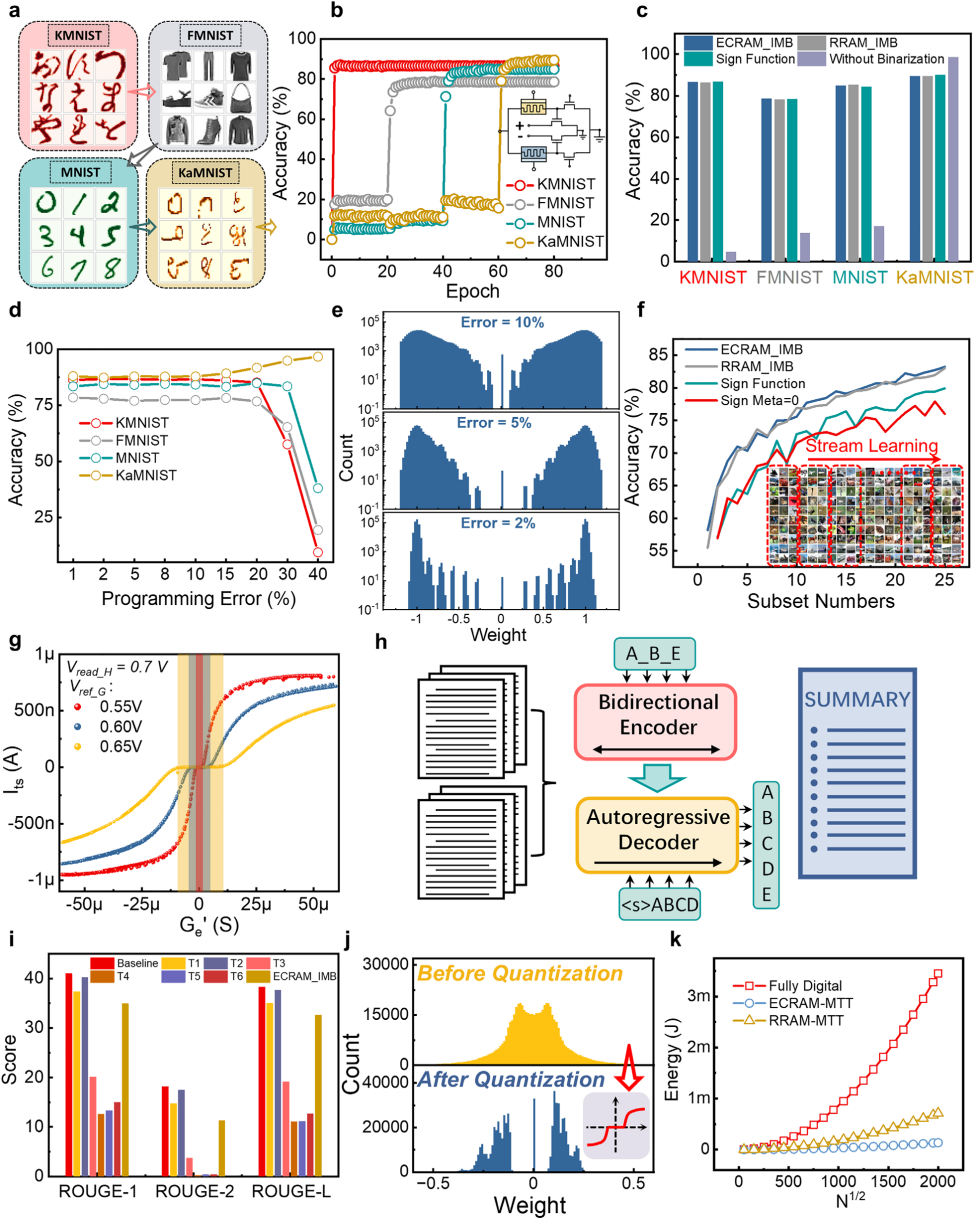
Large network applications and validation of in situ weight quantization relationships based on MTT units. a) Continual learning datasets for simulation validation, with each image in the different datasets sized at 28×28×1, and the datasets being independent of one another. b) Training results of continual learning for the datasets in panel (a), where the learning sequence is KMNIST‐FMNIST‐MNIST‐KaMNIST, employing a weight binary quantization function based on ECRAM MTT units. c) Statistical results of the final training accuracy across different datasets using various binary weight conversion functions. d) Network training performance is based on different relative programming error levels, where the network performance is almost unaffected when the error level is below 20%. e) The final distribution of hidden weights at the first layer of the network after training under different programming errors. f) Stream learning performances based on different binary weight conversion functions, with the dataset using Cifar‐10 and the network model being VGG‐7. g) Weight ternary quantization relationships based on MTT units, where the hidden weight bias voltage is set at 0.7 V, and the gate bias voltages of the reference transistor are set at 0.55, 0.60, and 0.65 V, respectively. h) Schematic diagram of the BART text summarization model application, which primarily consists of a bidirectional encoder and an autoregressive decoder. i) LLM learning results of the weight conversion relationships of MTT units under different bias conditions, where both the model input and activation functions undergo relevant quantization. j) Distribution diagrams before and after quantization of some weights based on the T‐2 weight conversion relationship. k) Energy consumption comparison between in‐place quantization based on MTT units and weight conversion on traditional CMOS digital platforms.

A larger‐level neural network simulation is necessary to demonstrate the practicality of MTT units. Four non‐overlap datasets are shown in Figure 7a, which are Kuzushiji MNIST (KMNIST) [60], Fashion MNIST (FMNIST), MNIST and Kannada MNIST (KaMNIST) [61]. All the image sizes in the datasets are 28×28×1, thus the neural network can keep the same structure in training sequentially. In this metaplasticity‐inspired algorithm, the analog hidden weights serve as important factors behind the binary weights, and two sets of mixed‐precision weights are essential in continual learning, which is verified in Figure S31a where the catastrophic forgetting happens without the binarization. This demonstrates that low‐precision quantization is crucial not only for resource efficiency but also for maintaining fundamental network performance in continual learning scenarios.

To optimize memory efficiency, we quantize hidden weights into discrete states with a boundary of ±1. As pretested (Figure S32), 3‐bit precision maintains continual learning capability, and is therefore adopted for both ECRAM‐MTT and RRAM‐MTT units. Figure 7b and Figure S31b show the training processes, while Figure 7c compares final accuracies across binarization methods, confirming the IMB function's effectiveness. Programming error tolerance is critical for weight mapping. Figure 7d demonstrates that ECRAM‐MTT units tolerate up to 20% programming noise (with weight distributions in Figure 7e). However, larger errors accumulate during training, causing significant performance degradation with increasing epochs (Figure S33). Table S4 quantifies this trade‐off between training duration and accuracy under varying programming errors, highlighting the need for careful balance.

Apart from continual learning of different tasks, another variant is stream learning, which is utilized to tackle the situation where only part of the training data are available at a given time. As shown in Figure S34 and Figure 7f, both FCNN and CNN architectures (see the Experimental Section for details) demonstrate successful stream learning using the weight transfer function, confirming its utility in both ECRAM‐MTT unit and RRAM‐MTT unit.

For low‐precision quantization, ternary quantization possesses better expressive abilities than binary precision counterparts, and its basic function is:

(9) $$W_{T}=\left\{\begin{array}{c} +1,\;\;W_{h}>\Delta \\ 0,\;\;|W_{h}|\leq \Delta \\ \;-1,\;\;W_{h}<-\Delta \end{array}\right.\;$$

where Δ is the positive threshold parameter, and *W_T_* and *W_h_* are ternary inference weights and real‐valued hidden weights, respectively. The zero‐value region is an important feature in ternary quantization, whereas it does not exist in binarization weight transfer in MTT units. However, by biasing the MTT unit at sub‐threshold regions, as previously predicted in Figure S12 where *W_b_* will always be about 0 at a small value of *W_h_*, it is possible to serve as a physical ternary weight transfer function. By controlling the hidden weight reading bias voltages, which usually are smaller than *V_T_* of the inference transistor, ternary weight quantization function can be well fitted, as experimental tested in Figure 7g and Figure S35a, where the relation between transferred ECRAM conductance $G_{e}^{\prime }$ and original ECRAM conductance *G_e_* is:

(10)

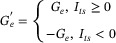




Here, *I_ts_* is the channel current of the inference transistor measured at the source terminal, and the fitting model is based on:

(11) $$\begin{array}{c} \;W_{T}=\left\{\begin{array}{c} 2\times \left(\frac{A_{p}\times \left(W_{h}-r\right)+1}{B_{p}\times \left(W_{h}-r\right)+2}-0.5\right),\;W_{h}>r\\ 0,\;\;|W_{h}|\leq r\\ -2\times \left(\frac{A_{n}\times \left(W_{h}+r\right)+1}{B_{n}\times \left(W_{h}+r\right)+2}-0.5\right),\;\;W_{h}<-r \end{array}\right. \end{array}$$

where *r* is the threshold parameter or the radius of zero regions, and as for other parameters, the previous model can be referred to. Fitting parameters of the ternary weight transfer function in the different bias conditions with corresponding notes are listed in Table S5. For the network‐level verification of the ternary weight transfer function in the sub‐threshold ECRAM‐MTT unit, BART, a kind of LLM featuring a bidirectional encoder similar to BERT and a left‐to‐right decoder akin to GPT, whose low‐bit version are used in resolving text summarization tasks [62], is employed to test (Figure 7h). The statistics‐based quantization algorithm in Liu's work [62] is adopted, including ternary and binary version of the BART model. Recall‐Oriented Understudy for Gisting Evaluation (ROUGE) is employed to evaluate automatic text summarization, which is based on comparing the overlap between generated text (e.g., abstracts) and reference text (e.g., human‐written abstracts). The variants include ROUGE‐1, ROUGE‐2 and ROUGE‐L, and the higher the scores are, the better the model performance is. When the model input and activation are under the relevant quantization, the LLM performances of the different weight transfer functions in MTT units are summarized in Figure 7i, including both ternary and binary quantization. T2‐bias based ternary weight transfer function corresponds to the most outstanding performance among all the tested conditions, where hidden weight bias voltage is 0.7 V and reference gate bias voltage is 0.6 V. Compared with baseline in software, final results only lag behind by 0.80, 0.75, and 0.67 at ROUGE‐1, ROUGE‐2, and ROUGE‐L, respectively. The weights before and after T2 ternary quantization are partly demonstrated in Figure 7j, in which the zero‐region feature is reflected in weight transfer. In addition, when the model input and activation functions are both in 8‐bit float format, as tested in Figure S35b, ECRAM‐MTT‐based binarization is the best choice for quantization. Thus, the physical quantization scheme should be adjusted according to the specific neural network.

In the energy estimation during the weight quantization and saving process, for the digital hardware platform, A100 GPU is adopted as the processor with Samsung 980 PRO NVMe solid‐state drive as the nonvolatile memory for storage of analog weights and binary weights. The evaluation of the MTT‐unit‐based array is presented in Note S1, which is mainly based upon the experimentally measured device data, including the average number of Write‐Verify times and the average power consumption of programming and reading once. Figure 7k shows the energy comparison in the quantization computation and storage process based on different hardware architectures. As the array scale (N) increases, the MTT unit saves more energy in the weight quantization process. When the array scale is 4Mb, the ECRAM‐MTT and RRAM‐MTT platforms save 25.51× and 4.84× of energy consumption, respectively. To evaluate the performance of the MTT unit in the synaptic unit function, it is compared with some related works based on hybrid synaptic units, mainly from the aspects of unit device type, structural complexity, programming characteristics and network computing characteristics. As shown in Table 1, this work only requires 6 components with significant reduction in unit complexity [25, 38, 40, 63, 64]. It supports in situ weight conversion without additional secondary programming, supports mixed precision calculations, possesses scalable unit structure, and is able to be applied to RRAM memory types with a wider range of application scenarios, including BNN‐based continual learning and low‐precision LLM.

**TABLE 1:** Evaluation of hybrid synaptic unit for separate training and inference.

Training device (TD)	Inference device (ID)	Unit complexity	Bit precision of TD	Nonvolatility of TD	Programming‐free ID	In situ weight transfer	In situ inference	Mixed‐precision unit	Application	Refs.
Capacitor	PCM	2×3T1C+2T2P	>8	No	No	Yes	Yes	No	Transfer Learning	[63]
RRAM	Transistor	2×2T1R	1	Yes	Yes	Yes	Yes	No	Transpose, Margin Enhancement	[25]
FeFET	FeFET	2×2T1D‐FeFET	8	Yes	No	Yes	Yes	No	Transfer Learning	[64]
Digital	RRAM	FPGA+2×1T1R	Floating‐point	No	No	No	Yes	Yes	BNN	[40]
FeRAM	RRAM	8T8C+1T1R	3	Yes	No	Yes	No	Yes	BNN	[38]
**ECRAM**	**Transistor**	**2×2T1E**	**5**	**Yes**	**Yes**	**Yes**	**Yes**	**Yes**	**BNN, TNN‐LLM**	**This work**

*Note*: For a more fair comparison, unit complexity is based on the differential structure in CIM.

## 3 Conclusion

In this work, for in situ accelerating low‐precision quantization computations of edge networks, we have developed an in situ quantization hardware unit based on ionic non‐volatile memory‐transistor coupling integration. By in‐situ quantization of analog weights, the traditional in‐memory computing paradigm has been expanded. Our research focuses on revealing the physical mechanism of linear conductance regulation of ECRAM devices, promoting the precise mapping of network analog weights in online training, and clarifying its stable conductance regulation mechanism through a combination of experimental characterization and theoretical modeling. By establishing a theoretical model for device parameter optimization, we systematically study the impact of bias conditions on the in situ quantization function of weights. Moreover, this research successfully extends the technical solution to the RRAM system, and based on its comprehensive performance optimization of switching ratio tolerance, it can also replace the ideal quantization function in software. Experiments show that the classification accuracy of the quantization function based on the electrical characteristics of the unit structure in the binary neural network is consistent with the ideal function, which can support low‐precision continual learning to overcome the catastrophic forgetting problem of traditional neural networks and binary/ternary large language model calculations as well. At the 4 Mb array scale, during the computation and saving of the quantization process, the quantization units based on ECRAM and RRAM achieve 25.51 and 4.84 times energy consumption reductions compared to traditional digital platforms, respectively, providing a more energy‐efficient in situ quantization calculation solution for low‐precision network training at the edge.

## 4 Experimental Section

### 4.1 ECRAM Array Fabrication

The fabrication of the ECRAM array utilized silicon substrates featuring 300 nm of thermally grown silicon dioxide. The process began with electron‐beam lithography to create patterns for the source‐drain contacts, after which 5 nm of Ti and 25 nm of Au were deposited using electron‐beam evaporation. The contact electrodes, essential for monitoring changes in channel conductance, were finalized through a lift‐off procedure. Following this, a second round of electron‐beam lithography was employed to define the channel area, where 60 nm of tungsten trioxide (WO_x_) was deposited by radio frequency magnetron sputtering, acting as the ECRAM channel. After the lift‐off, the electrolyte region was patterned using a third round of electron‐beam lithography, during which 120 nm of LiPON was deposited from a Li_3_PO_4_ target using radio frequency magnetron sputtering under a nitrogen flow of 10 sccm and at a power setting of 150 W. Subsequently, 40 nm of SiO_x_ was deposited to shield the electrolyte from environmental moisture. To isolate the different wires, 40 nm of SiO_x_ was used after the fourth round of electron‐beam lithography. Finally, a fifth round of electron‐beam lithography was utilized to pattern test pads, which were then deposited with 10 nm of Ti and 220 nm of Au via electron‐beam evaporation.

### 4.2 Electrical Measurements

All the electrical measurements were performed using an Agilent B1500A semiconductor analyzer and the electrode pad lead‐out was realized by the TS3000 Probe System. Except for device size exploration, the devices size used in this paper are all kept at 10 µm × 10 µm.

### 4.3 Material Characterization

To analyze the material components of fabricated devices, the TEM samples were prepared first by the focus ion beam technique (Helios G5 UX). After that, TEM testing as well as EDS imaging were performed on Talos F200X G2 systems. TOF‐SIMS tests of WO_x_‐based ECRAM were conducted with a PHI nano TOF II (ULVAC‐PHI Inc., Japan). The sputter etching was performed using an Ar^+^ beam (3 kV 100 nA) to obtain the depth profile of tested samples for different resistance states.

### 4.4 Transistor Characteristics Used in Model Prediction

For a nMOSFET, while *V_gs_* ≥ *V_T_*, if *V_ds_* ≪  *V_gs_* − *V_th_*, the drain current *I_ds_* can be written as:

(12) $$\begin{array}{c} \;I_{ds}=\mu_{eff}\;\times C_{ox}\times \frac{W}{L}\times \left(V_{gs}-V_{T}\right)\times V_{ds}+bias \end{array}$$

where *V_T_* is the threshold voltage of the transistor, *V_gs_* and *V_ds_* are the gate‐source voltage drop and drain‐source voltage drop, respectively, µ_*eff*_ is the effective carrier mobility, *C_ox_* is the oxide capacitance per unit area, *W* and *L* are the width and length of the transistor, respectively, and the *bias* is the compensation. When *V_gs_* < *V_T_*, the transistor will be in the subthreshold region, the channel current *I_ds_* can be written as:

(13) $$\begin{array}{c} \begin{array}{ccc} & & \;I_{ds}=\mu_{eff}\times C_{ox}\times \frac{W}{L}\times \left(m-1\right)\times {T^{\prime }}^{2}\\ & & \times exp\left(\frac{V_{gs}-V_{T}}{m\times T^{\prime }}\right)\times \left(1-\exp\left(-\frac{V_{ds}}{T^{\prime }}\right)\right) \end{array} \end{array}$$

Here, $T^{\prime }=\frac{k\times T}{q}$, where *k* is the Boltzmann constant, *T* is the absolute temperature, *q* is the charge of electrons, and *m* is the subthreshold slope factor. The above parameter values are concluded in Table S1.

### 4.5 Neural Network Simulations in MTT Model Derivation

The metaplasticity‐inspired BNN is adopted to test the weight transfer functions in the MTT model, where the main body of this algorithm is the BNN, whose weight values and neuron activations are limited to be 1 or −1. The distinctions between this approach and a traditional binary neural network are in the training process, where the updated gradients must be multiplied by a nonlinear function, which is associated with the values of the hidden weights and is influenced by a hyperparameter m, which will be fixed at 12. The tested image sizes are all set as 28×28; the network structure is kept at 784×1000×500×10; the learning rate is set at 0.005; the hidden weights are uniformly quantized into 3 bits with quantization boundary of ±1.

### 4.6 Quantized Hidden Weights and Binary Weights from Physical Electrical Signals

By taking the weight transfer function into neural network simulation, the relationship between quantized hidden weights *W_h_* and ECRAM conductance *G_e_* is:

(14) $$\begin{array}{c} \;W_{h}=\left\{\begin{array}{c} \frac{G_{e}-G_{e,min}}{G_{e,max}-G_{e,min}},\;I_{ts}\geq 0\\ \frac{-G_{e}+G_{e,min}}{G_{e,max}-G_{e,min}},\;\;I_{ts}<0 \end{array}\right. \end{array}$$

where *I_ts_* is the channel current of the inference transistor, *G*_*e*,*max*_ and *G*_*e*,*min*_ are maximum and minimum ECRAM conductance during programming, respectively. The relationship between quantized binary weights *W_b_* and channel current of the inference transistor *I_ts_* is:

(15) $$\begin{array}{c} \;W_{b}=\frac{I_{ts}}{I_{ts,max\;}} \end{array}$$

where *I*_*ts*,*max*_ is the channel reading current when the gate is biased at $V_{read\_H}$, which is also the maximum channel current at infinite ECRAM conductance.

### 4.7 Weight Transfer Function Used in Neural Network Training

During all the network simulations unless otherwise specified, the MTT transfer function in ECRAM‐MTT units is selected when the maximum operating conductance is 50 µS, and the specific function is:

(16) $$\begin{array}{c} \;W_{b}=\left\{\begin{array}{c} 2\times \left(\frac{41.36\times W_{h}+1}{41.62\times W_{h}+2}-0.5\right),\;\;W_{h}\geq 0\\ -2\times \left(\frac{-44.67\times W_{h}+1}{-44.45\times W_{h}+2}-0.5\right),\;\;W_{h}<0 \end{array}\right. \end{array}$$

While for RRAM‐based MTT units, the weight transfer function is:

(17) $$\begin{array}{c} \;W_{b}=\left\{\begin{array}{c} 2\times \left(\frac{63.61\times W_{h}+1}{63.67\times W_{h}+2}-0.5\right),\;\;W_{h}\geq 0\\ -2\times \left(\frac{-70.03\times W_{h}+1}{-67.44\times W_{h}+2}-0.5\right),\;W_{h}<0 \end{array}\right. \end{array}$$

### 4.8 ECRAM‐ or RRAM‐Based IMB for Edge Neural Network Simulation

For testing IMB in MTT units in edge light‐weight neural networks, two kinds of common models, FCNN and CNN, are employed. As for FCNN, Fashion‐MNIST is chosen for classification, with neural network size of 784×1000×500×10 and learning rate of 0.005. For CNN simulations, the binary VGG‐7 with six convolution layers of 128‐128‐256‐256‐512‐512 filters and kernel sizes of 3 is employed, and the learning rate is 0.0005. Both neural network simulations of FCNN and CNN are run for 10 rounds, the average final accuracies and variations are shown in Table S3.

### 4.9 Neural Network Simulation for Continually Learning Small Tasks

For neural network simulation on the continual learning of different small tasks, neural network structure is set as 2×3×1; learning rate is 0.005; meta‐parameter m is 10; the quantized precision and boundary of hidden weights are 3 bits and ±1, respectively.

### 4.10 Stream Learning

Basic neural network structures are the same as edge neural network simulations. In the FCNN stream learning, the Fashion‐MNIST dataset is split into 40 subsets, and each subset is learned for 10 epochs, with meta parameter m set as 5. In the CNN stream learning, the Cifar‐10 dataset is split into 25 subsets, and each subset is learned for 20 epochs, with meta parameter m set as 10.

### 4.11 DFT Calculation

The structural properties of the material before and after Li atom doping were investigated through density functional theory (DFT) calculations using the Vienna Ab initio Simulation Package (VASP) [65, 66]. The projector augmented wave (PAW) plane‐wave basis set with the Perdew–Burke–Ernzerhof (PBE) [67] exchange‐correlation functional was employed in the computational framework. To account for the strong correlation effects of d electrons in transition metal elements, DFT+U [68] correction was implemented with Hubbard U values of 4.0 eV for Nb and 6.2 eV for W, respectively. Prior to electronic property calculations, structural relaxation was performed until the atomic forces converged below 0.02 eV/Å. In addition, the migration energy barriers of Li ions within different materials were determined using the climbing image nudged elastic band (CI‐NEB) method [69]. The amorphous structures were generated via melt‐quench molecular dynamics simulations starting from their crystalline phases. The amorphous WO_x_ structure was generated using a 128‐atom WO_3_ supercell, while the amorphous NbO_x_ structure was generated using a 168‐atom Nb_2_O_5_ supercell.

## Conflicts of Interest

The authors declare no conflicts of interest.

## Supporting information




**Supporting file**: advs73957‐sup‐0001‐SuppMat.docx.

## Data Availability

The data that support the findings of this study are available from the corresponding author upon reasonable request.
