# Supplementary material for: In Situ Quantization with Memory‐Transistor Transfer Unit Based on Electrochemical Random‐Access Memory for Edge Applications
Source: Adv Sci (Weinh). 2026 Jan 21;13(18):e21815. doi: 10.1002/advs.202521815 (PMC13042408; doi:10.1002/advs.202521815)
Supplement: Supplementary file 1 — Supporting file: advs73957‐sup‐0001‐SuppMat.docx. [file ADVS-13-e21815-s001.docx]

Supporting Information

**In-situ Quantization with Memory-Transistor Transfer Unit Based on Electrochemical Random-Access Memory for Edge Applications**

Zhen Yang^†^, Yuxiang Yang^†^, Baiqian Wang, Yaoyu Tao, Zelun Pan, Lei Cai, Teng Zhang, Longhao Yan, Xianbin Li*, Yuchao Yang*

*Corresponding authors: Y. Yang: [yuchaoyang@pku.edu.cn](mailto:yuchaoyang@pku.edu.cn), X. Li: [lixianbin@jlu.edu.cn](mailto:lixianbin@jlu.edu.cn)

^†^Equally contributed to this work.


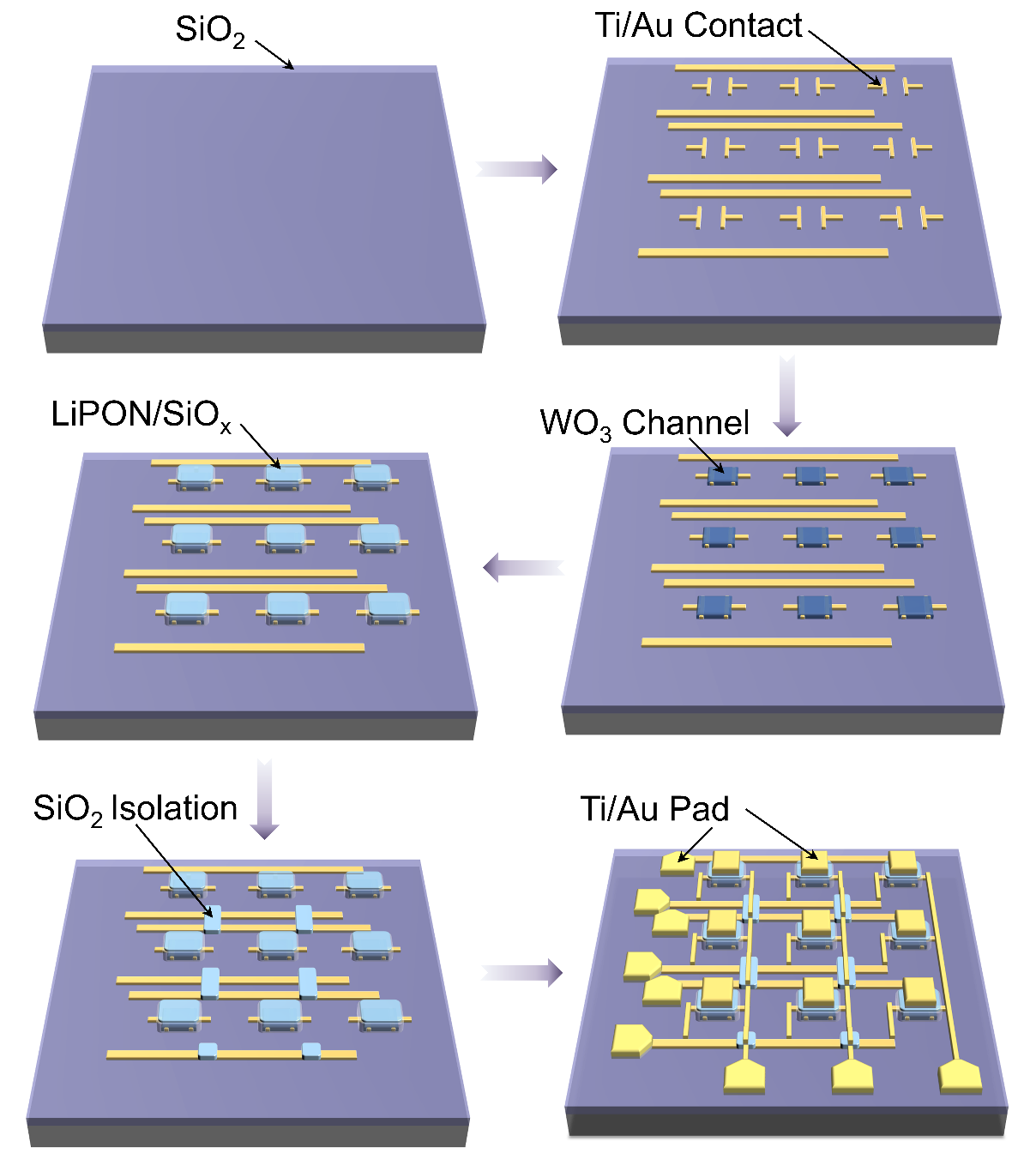


**Figure S1. Fabrication process flow of all-solid-state ECRAM arrays.** The SiO_2_ isolation layer was used to insulate the source line between the gate line and the drain line.

**
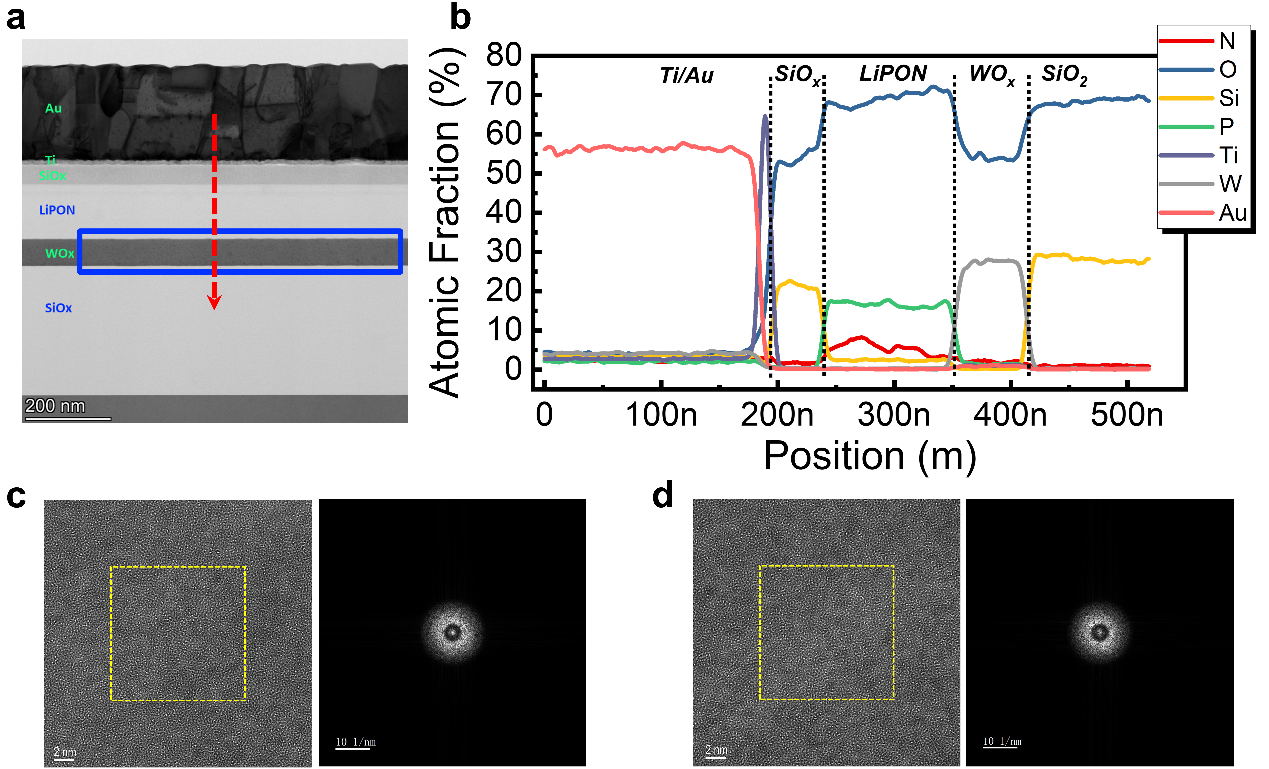
**

**Figure S2.** (a) TEM image of the cross-section of ECRAM core regions, in which it shows clear boundaries between different layers. (b) The EDS line scan of the main elements within the red arrow regions of (a). (c, d) HRTEM image of the WO_x_ layer and the corresponding diffraction pattern extracted by fast Fourier transformation for the yellow rectangular regions, which all prove the amorphous structure of the channel layer.


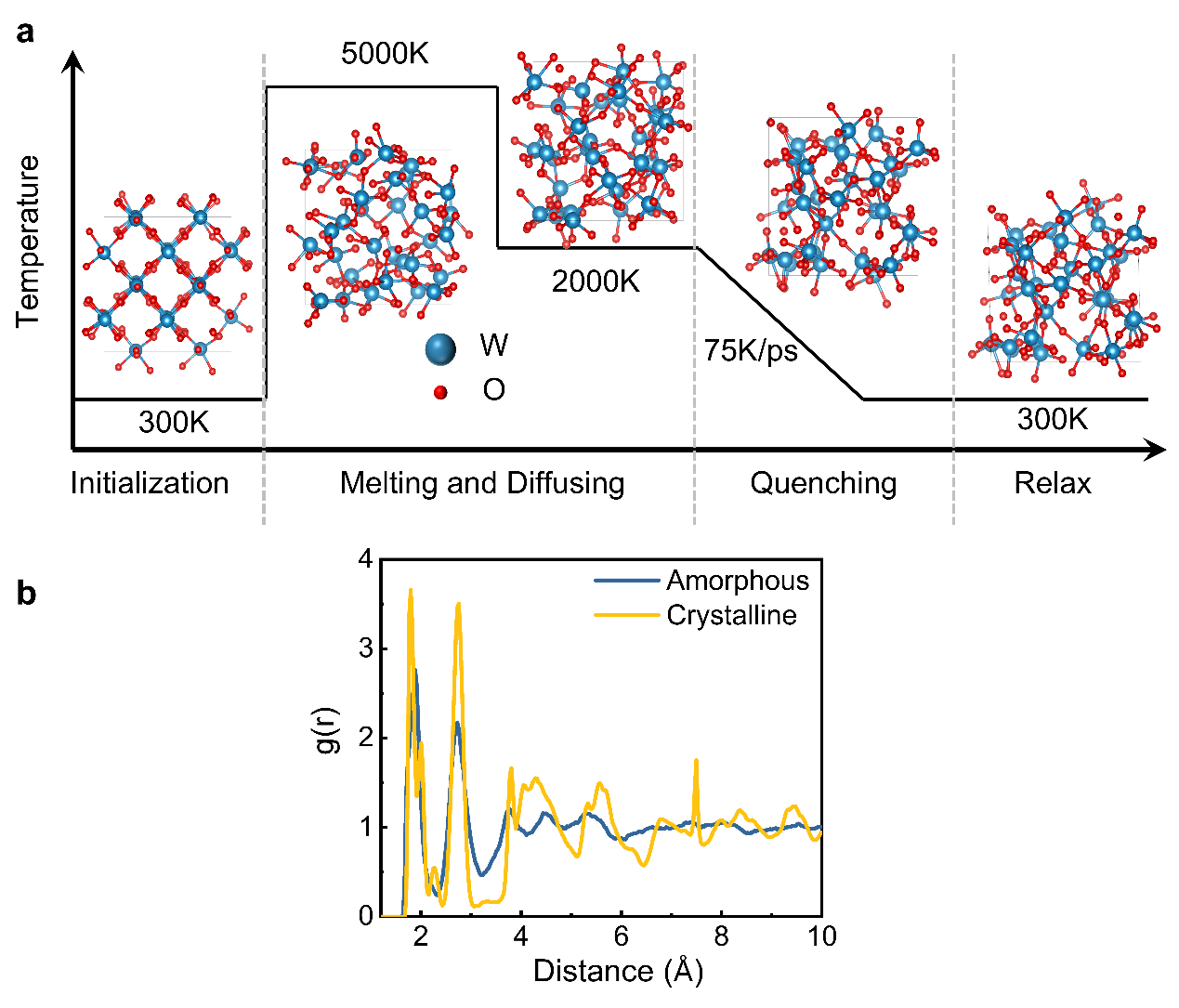


**Figure S3.** (a) Schematic illustration of the construction process of WO_x_ amorphous structure by using the melt-quenching and relaxing method. (b) Comparison of the radial distribution function in crystalline and amorphous structure of WO_x_ at 300K.


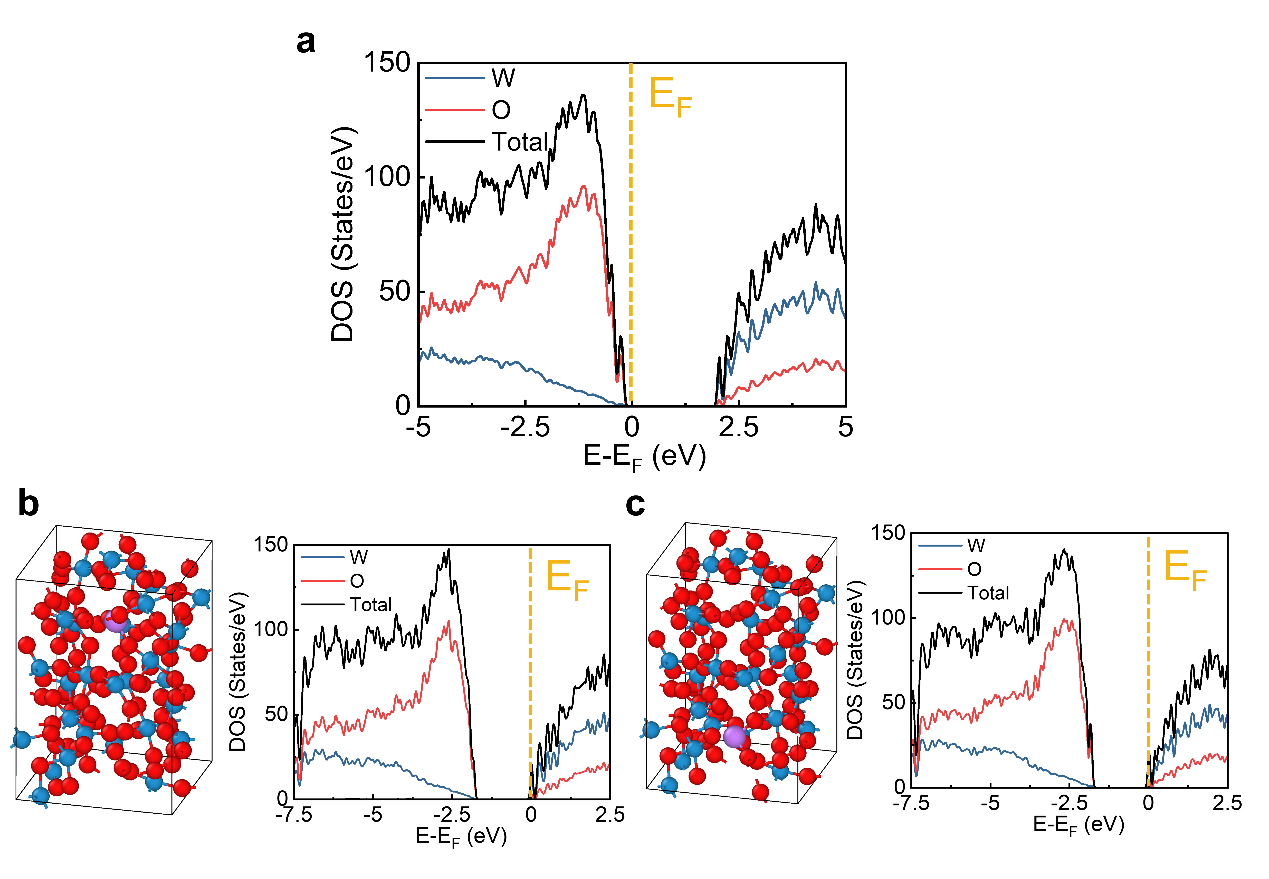


**Figure S4.** (a) The density of states (DOS) for the pristine amorphous WO_x_, which indicates a kind of semiconductor with a significant energy gap. (b, c) The density of states (DOS) of amorphous WO_x_ when Li elements were doped in two other different sites, displaying their n-type electron doping.


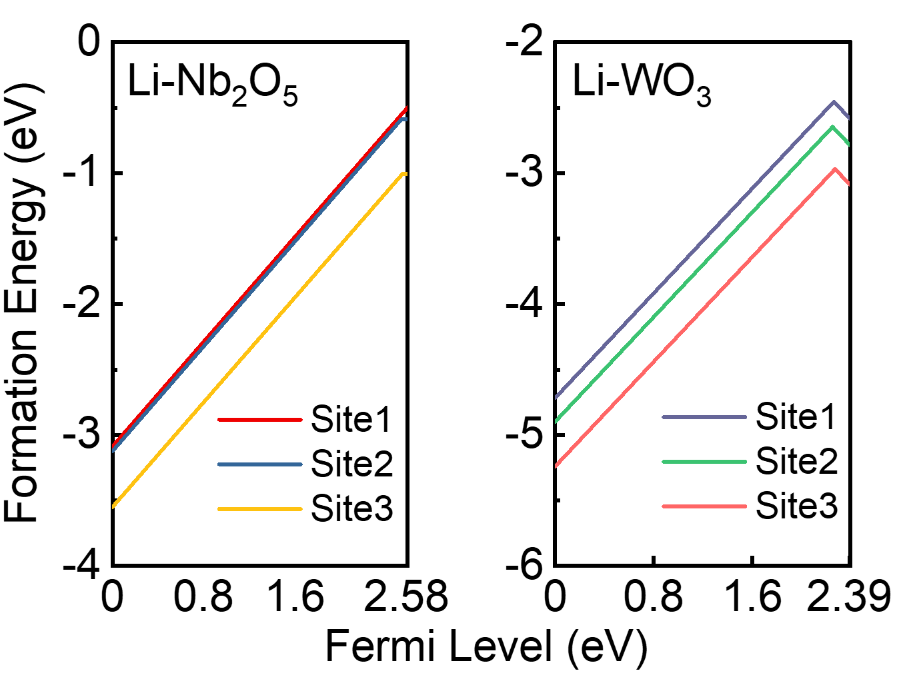


**Figure S5.** The relationship between the formation energy of interstitial Li defects and Fermi level for amorphous Nb_2_O_5_ (at Nb-rich condition) and amorphous WO_3_ (at W-rich condition), respectively.


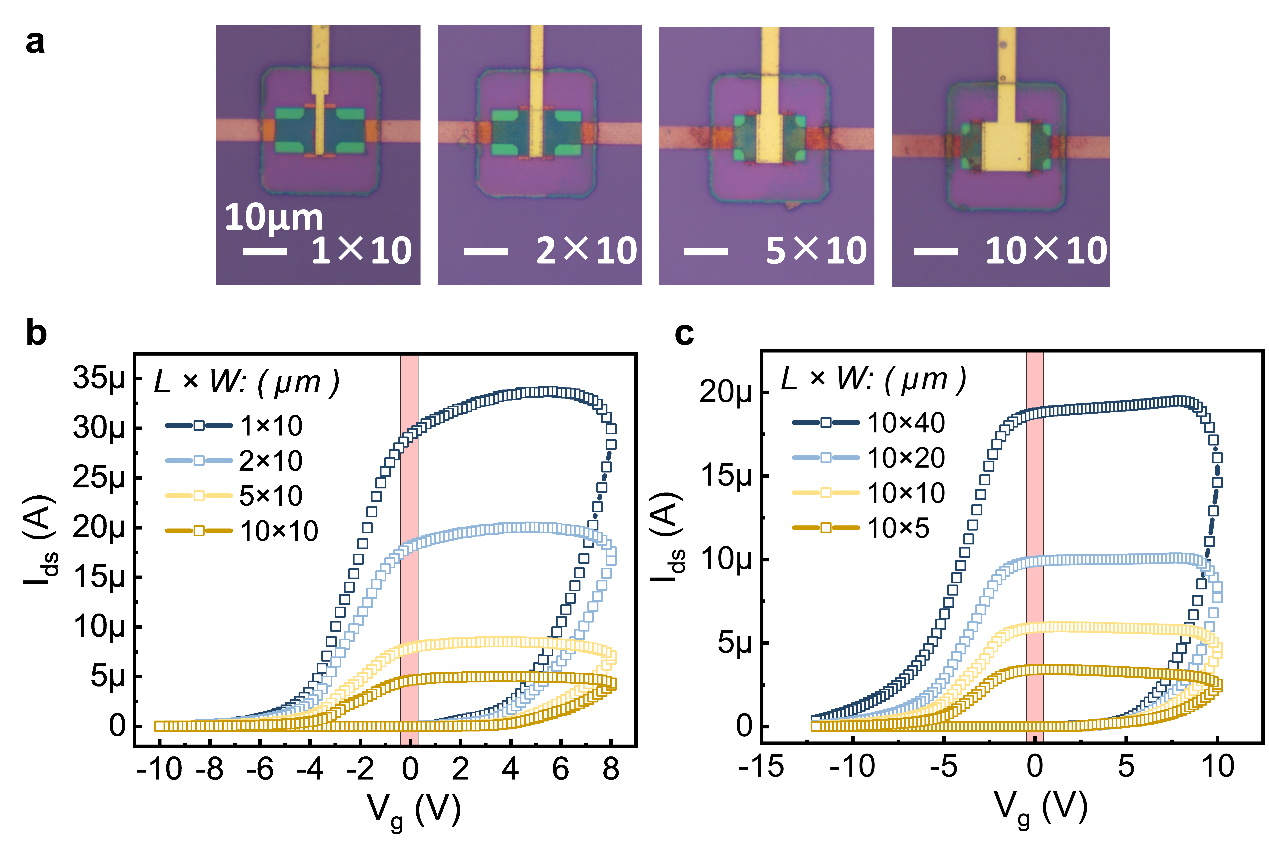


**Figure S6.** (a) Optical microscopy images of the fabricated ECRAM devices of different lengths, including 1, 2, 5 and 10 µm, with width fixed at 10 µm. (b, c) The DC characteristics of ECRAM devices in different sizes, including different lengths and widths.


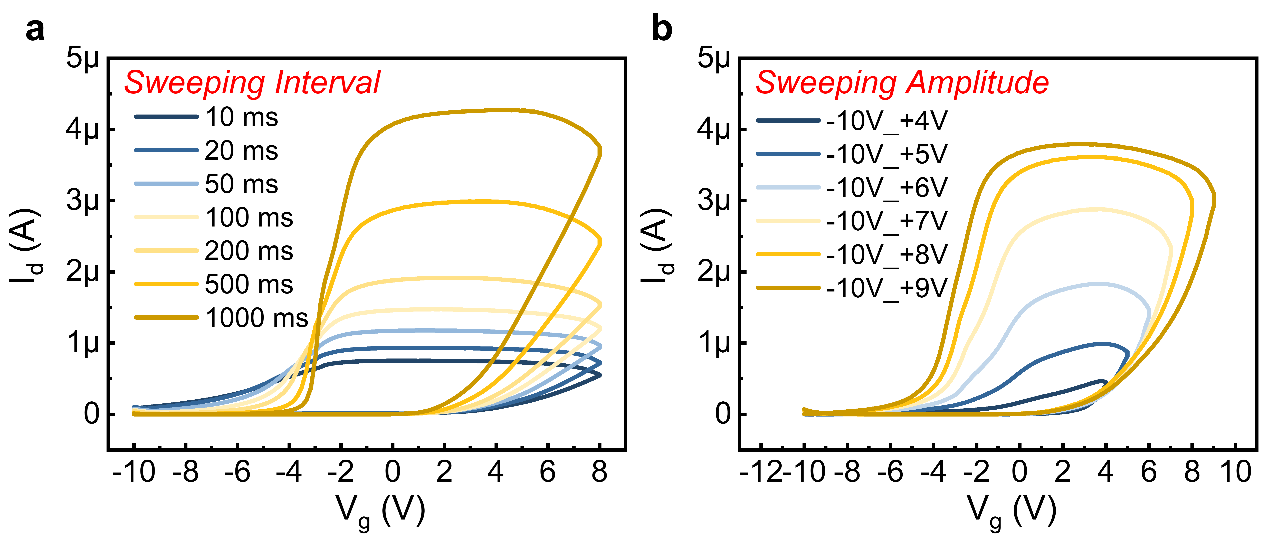


**Figure S7. Transfer characteristics of ECRAM in different sweeping rates (a) and sweeping ranges (b), and the switching window can be enhanced either by increasing the sweeping intervals or by increasing the sweeping amplitudes.**


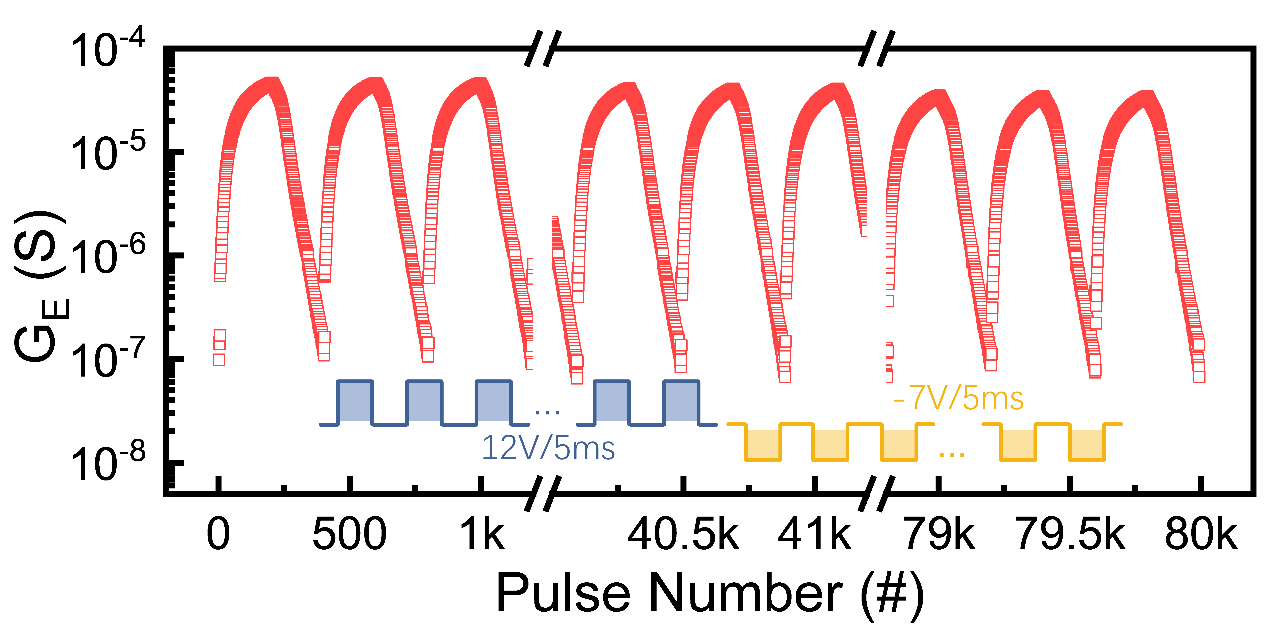


**Figure S8. The changes in ECRAM conductance concerning the programming pulses for the large switching window mode, in which the amplitudes of the potentiation and depression pulse are 12 V and -7 V, respectively, while the widths are both 5 ms, and each cycle of updating is composed of 200 potentiation pulses and 200 depression pulses.**


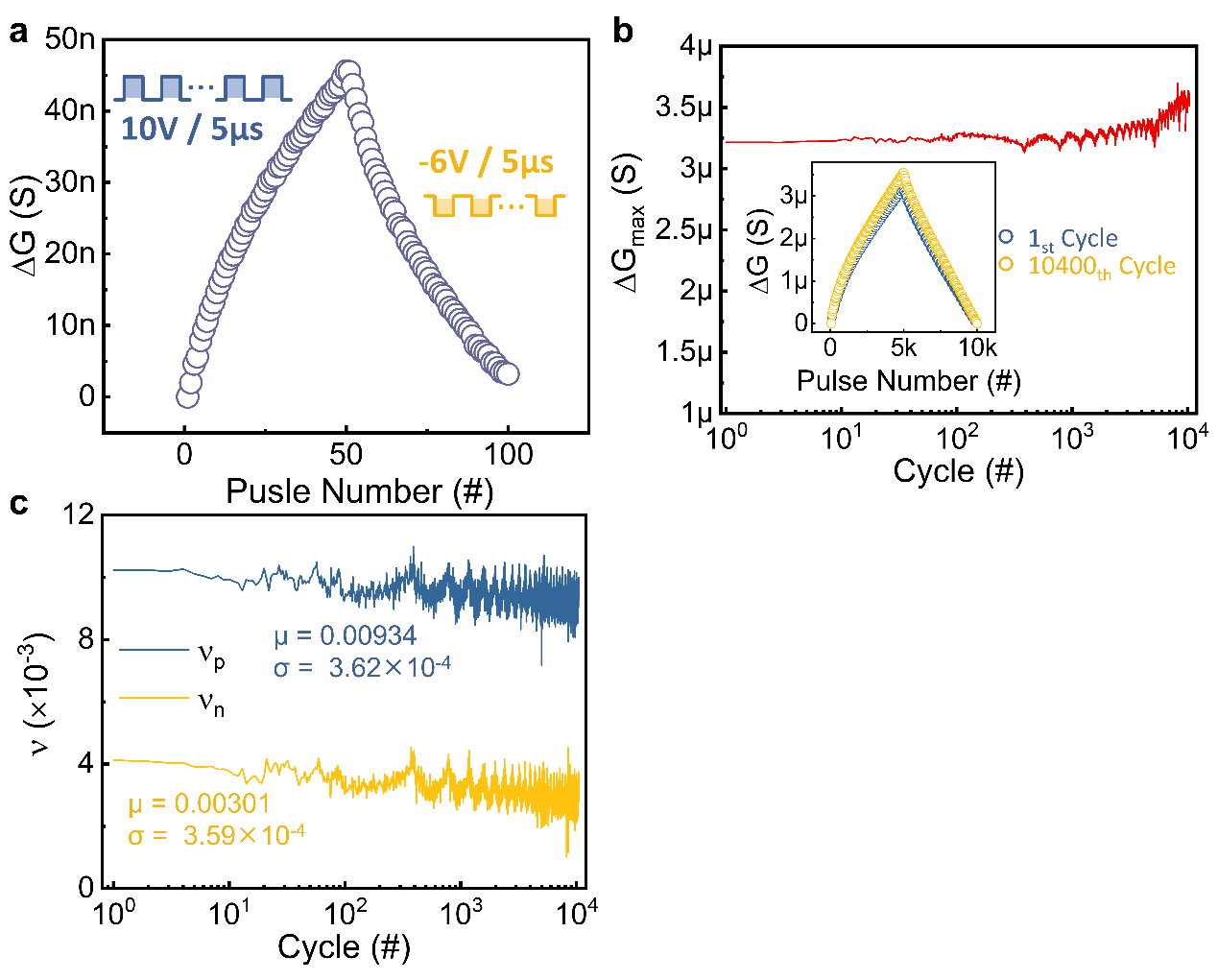


**Figure S9.** (a) Programming by µs-level pulse stimulation, in which the amplitudes of potentiation and depression pulses are 10 V and -6 V, respectively. (b) he evolution of maximum conductance changes during more than 10^4^ cycles of tests, the inset is the comparison of updating characteristics in the first cycle and the last cycle. (c) The linearity parameters of potentiation and depression programming during the endurance tests, and the related normal distribution fitting parameters are shown in the figure.


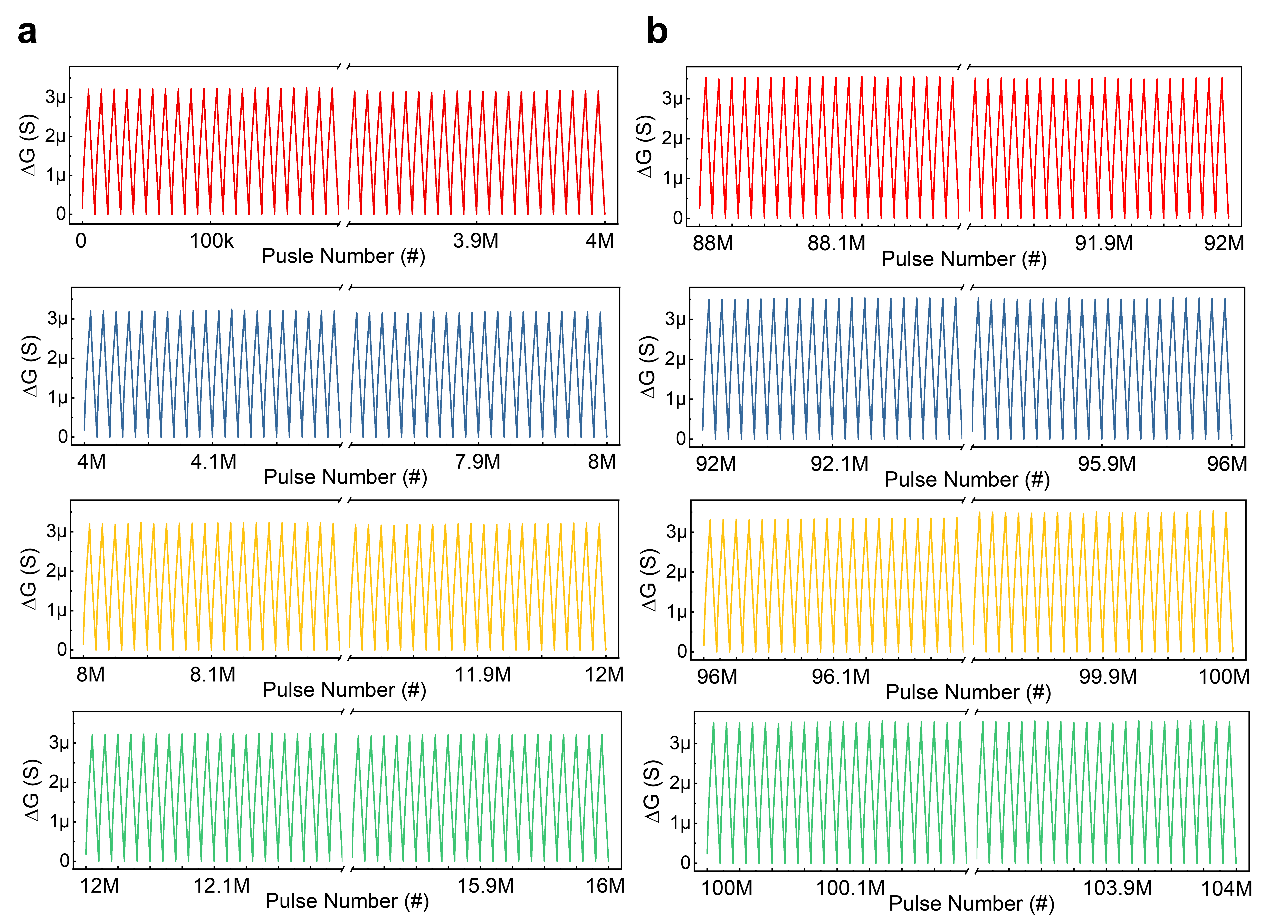


**Figure S10.** **Detailed conductance updating processes in the endurance tests for the first 16🞩10^6^ pulses (a) and the last 16🞩10^6^ pulses (b).**


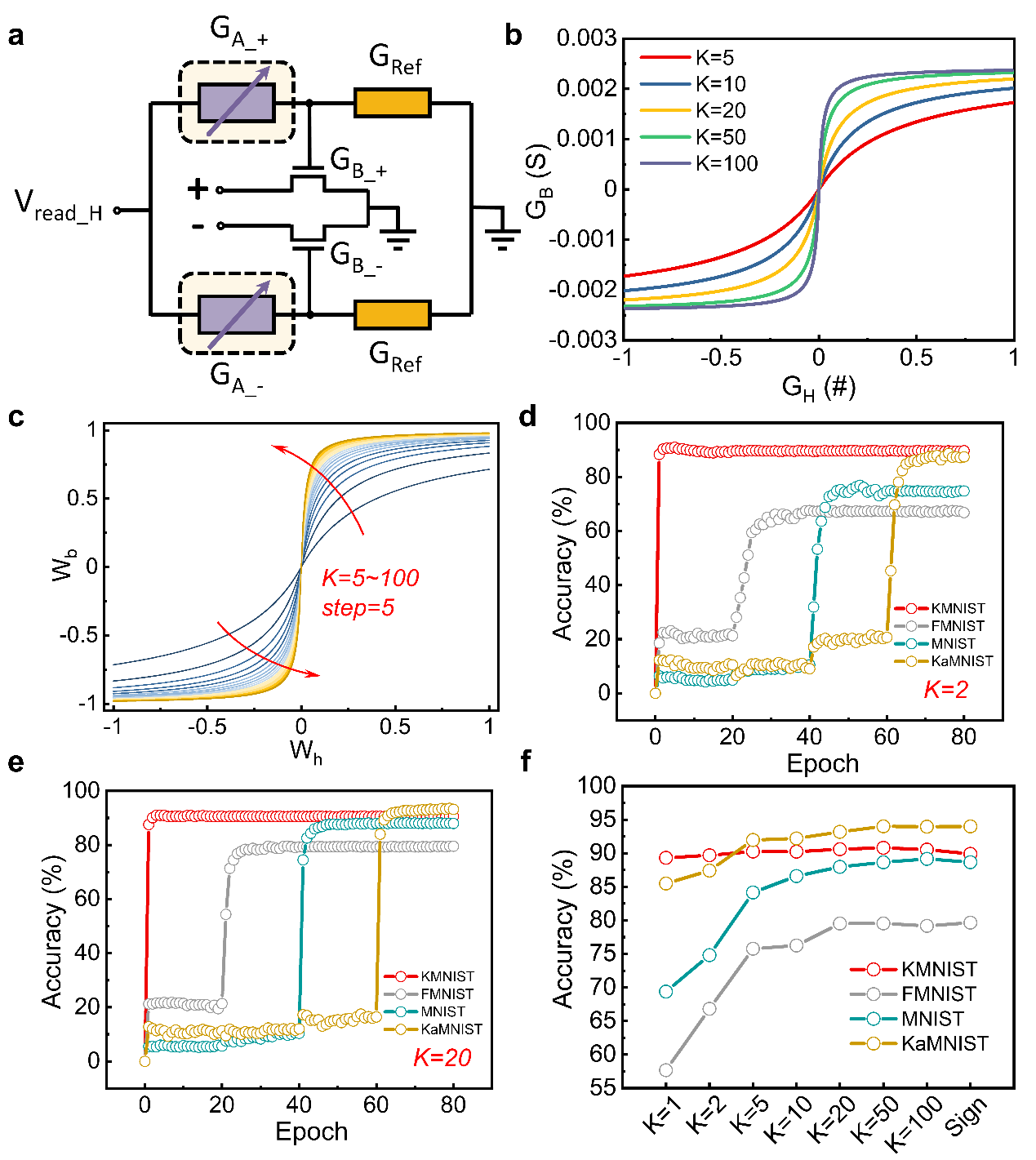


**Figure S11.** **Derivation of the theoretical model for the symmetrical MTT units.** (a) Schematic of the symmetrical MTT units, and the reference transistor is replaced by the reference resistor for simplicity, and the purple variable resistors represent the ECRAM or other non-volatile memory devices. (b) The differential inference transistor conductance versus quantized analog memory conductance at different on/off ratios, which is denoted by K. (c) Relationships between the quantized hidden weights and the binary weights in the MTT unit at different on/off ratios. (d, e) Neural network performances for continual learning based on the theoretically derived binary quantization function, in which the on/off ratios are 2 and 20, respectively. (f) Continual learning capacity based on the weight transfer functions of MTT units with different maximum on/off ratios, which also includes the baseline results obtained on the Sign function.


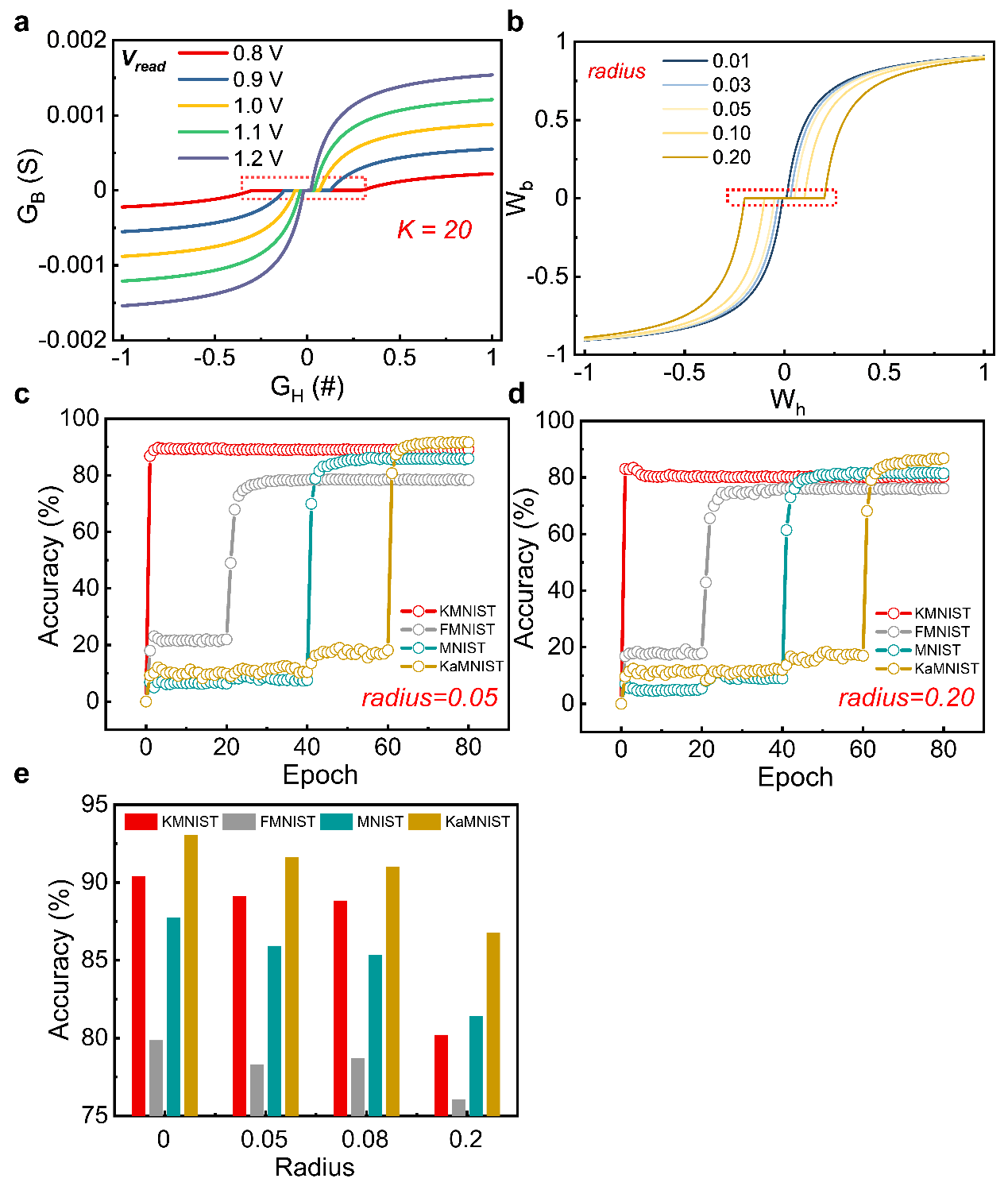


**Figure S12.** (a) The differential inference transistor conductance versus quantized analog memory conductance at different hidden weight bias voltage, in which the inference transistor will be in the sub-threshold regions while the analog memory conductance is not high enough, and the on/off ratio is fixed at 20. (b) Theoretically derived weight conversion relationship when the MTT unit is at sub-threshold reading conditions. (c, d) Neural network performances for continual learning based on the theoretically derived binary quantization function under the sub-threshold reading condition, in which the radius parameters are 0.05 and 0.20, respectively. (e) The comparison of continual learning capacity based on the sub-threshold weight transfer functions of MTT units at different radius parameters, all the on/off ratios are kept at 20.


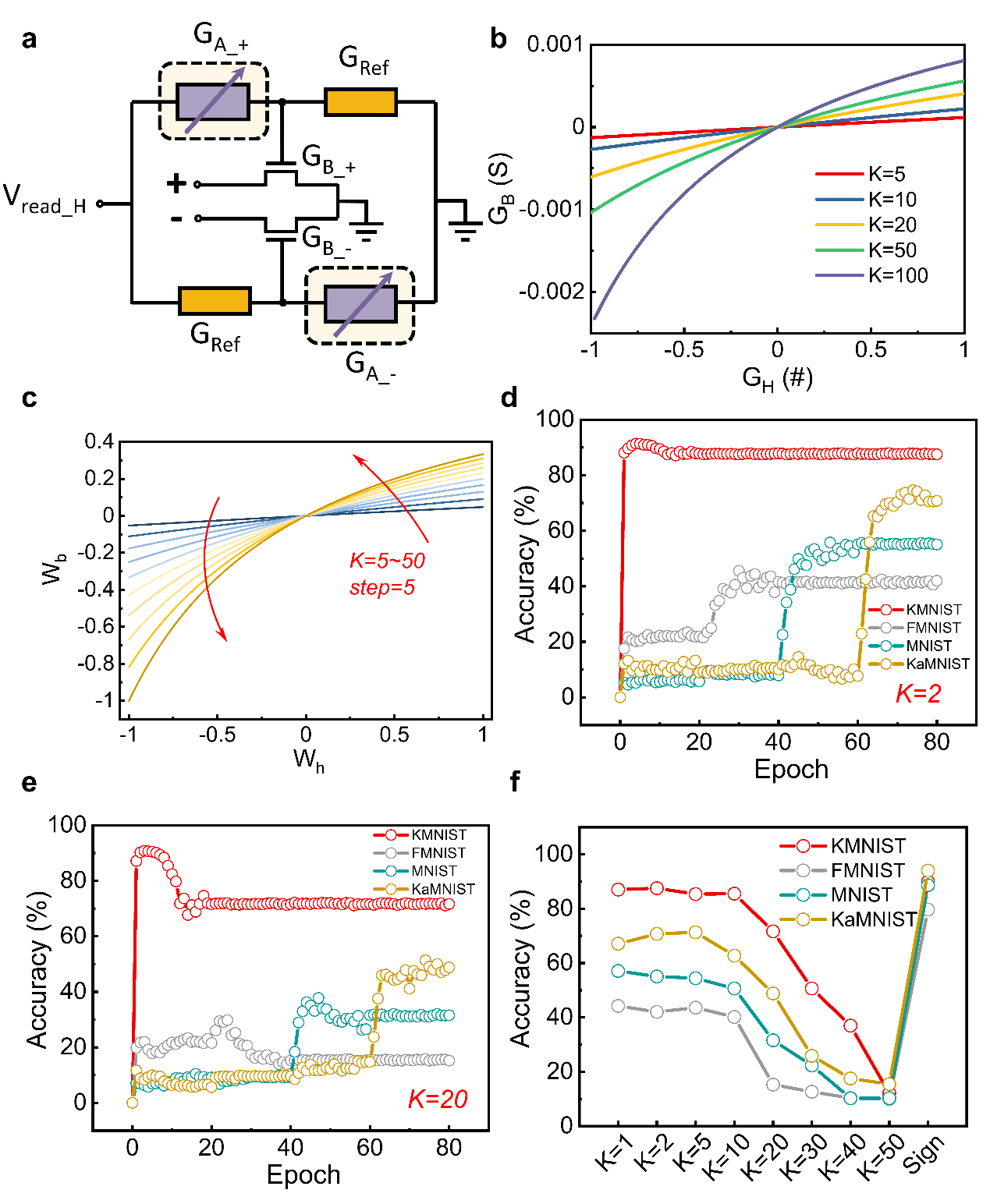


**Figure S13. Derivation of the theoretical model for the asymmetrical MTT units.** (a) Schematic of the asymmetrical MTT units, and the reference transistor is replaced by the reference resistor for simplicity, and the purple variable resistors represent the ECRAM or other non-volatile memory devices, and the conductance of the two devices are kept the same during the actual operations. (b) The differential inference transistor conductance versus quantized analog memory conductance at different on/off ratios, which is denoted by K. (c) Relationships between the quantized hidden weights and the binary weights in the asymmetrical MTT unit at different on/off ratios. (d, e) Neural network performances for continual learning based on the theoretically derived binary quantization functions from the asymmetrical MTT unit, in which the on/off ratios are 2 and 20, respectively. (f) Continual learning capacity based on the weight transfer functions of asymmetrical MTT units with different maximum on/off ratios, which also includes the baseline results obtained on the Sign function.


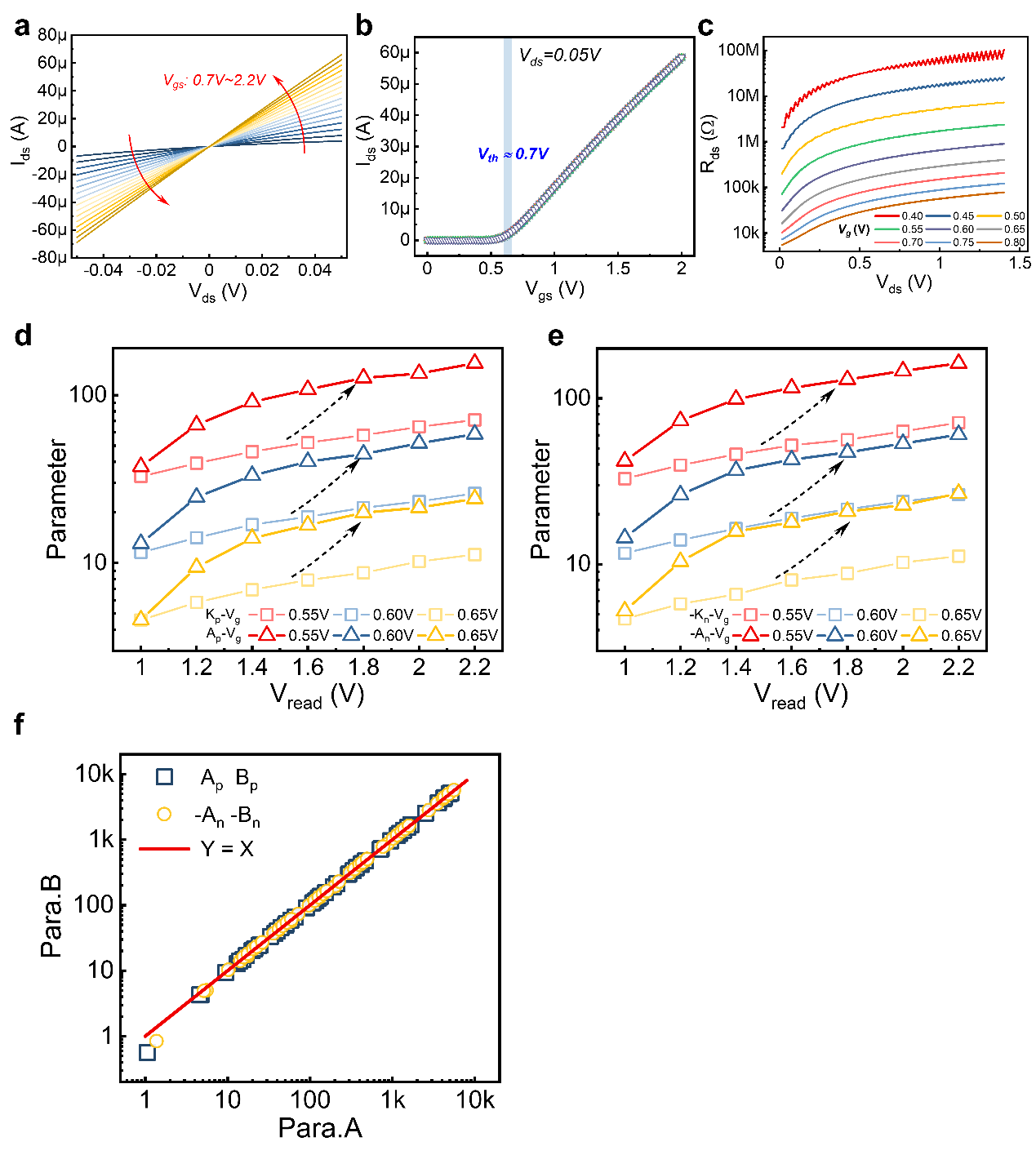


**Figure S14.** (a) Linear reading characteristics when the transistor is at the linear region, which can be configured as the analog multiplication and addition unit in CIM arrays. (b) Typical transfer characteristics of the tested transistor in this work, and V_ds_ is 0.05 V. (c) The channel resistance versus drain-source reading voltages at different gate biases. (d, e) Comparison between fitting parameters and on/off ratios at different reference gate biases and the hidden weight reading bias voltages. (f) The relationship of the parameter pair (A_p_, B_p_) and (A_n_, B_n_).


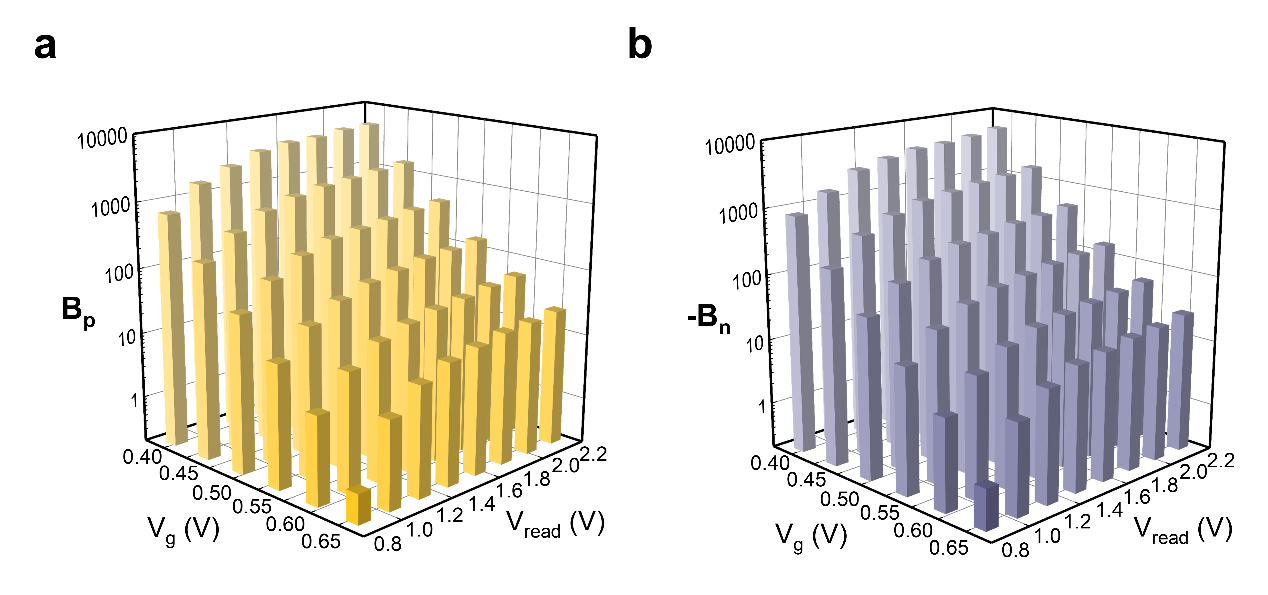


**Figure S15. Parameter fitting results for the B_p_ (a) and B_n_ (b) in the weight transfer function of the MTT unit at different hidden weight bias voltages and gate voltages of the reference transistor.**


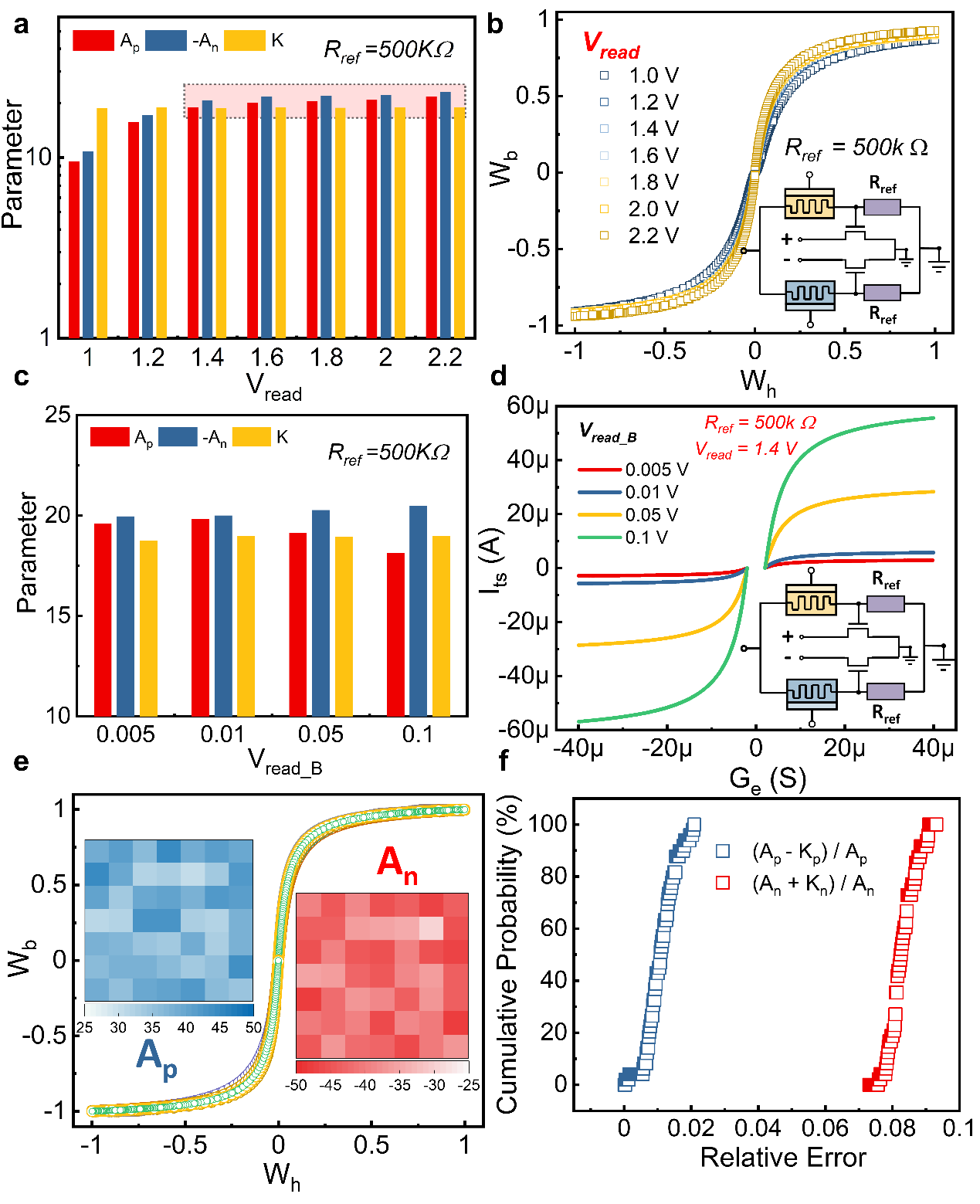


**Figure S16. Characterization of 1E1R1T-based MTT unit.** (a) Fitting parameters and operation switching ratio of the 1E1R1T-based MTT unit using different hidden weight reading bias voltages. (b) Relationships between the quantized hidden weights and the binary weights in the 1E1R1T-based MTT unit under different hidden weight reading bias voltages, in which the reference resistor is fixed at 500 kΩ. (c) Fitting parameters and operation switching ratio of the 1E1R1T-based MTT unit using different binary weight reading bias voltages, which is implemented at the drain terminal of the inference transistor. (d) Relationships between the quantized hidden weights and the binary weights in the 1E1R1T-based MTT unit under different binary weight reading bias voltages, in which the reference resistor is fixed at 500 kΩ and hidden weight reading voltage is kept at 1.4 V. (e) Statistical data on the weight transfer function in the 1E1R1T-based MTT unit among the 49 different MTT units, and the summary data of the gap between the fitting parameters and the on-off ratio is also shown in (f).


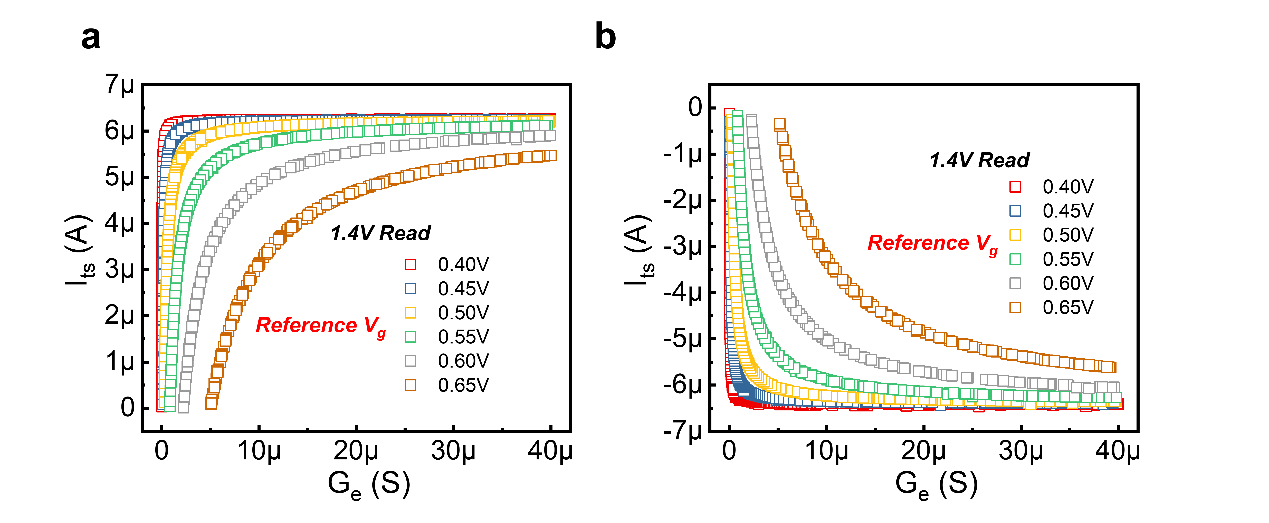
**Figure S17. Positive (a) and negative (b) relationships between the conductance of ECRAM and the channel current of the inference transistor under different reference gate bias voltages, while the hidden weight reading voltage is kept as 1.4 V.**


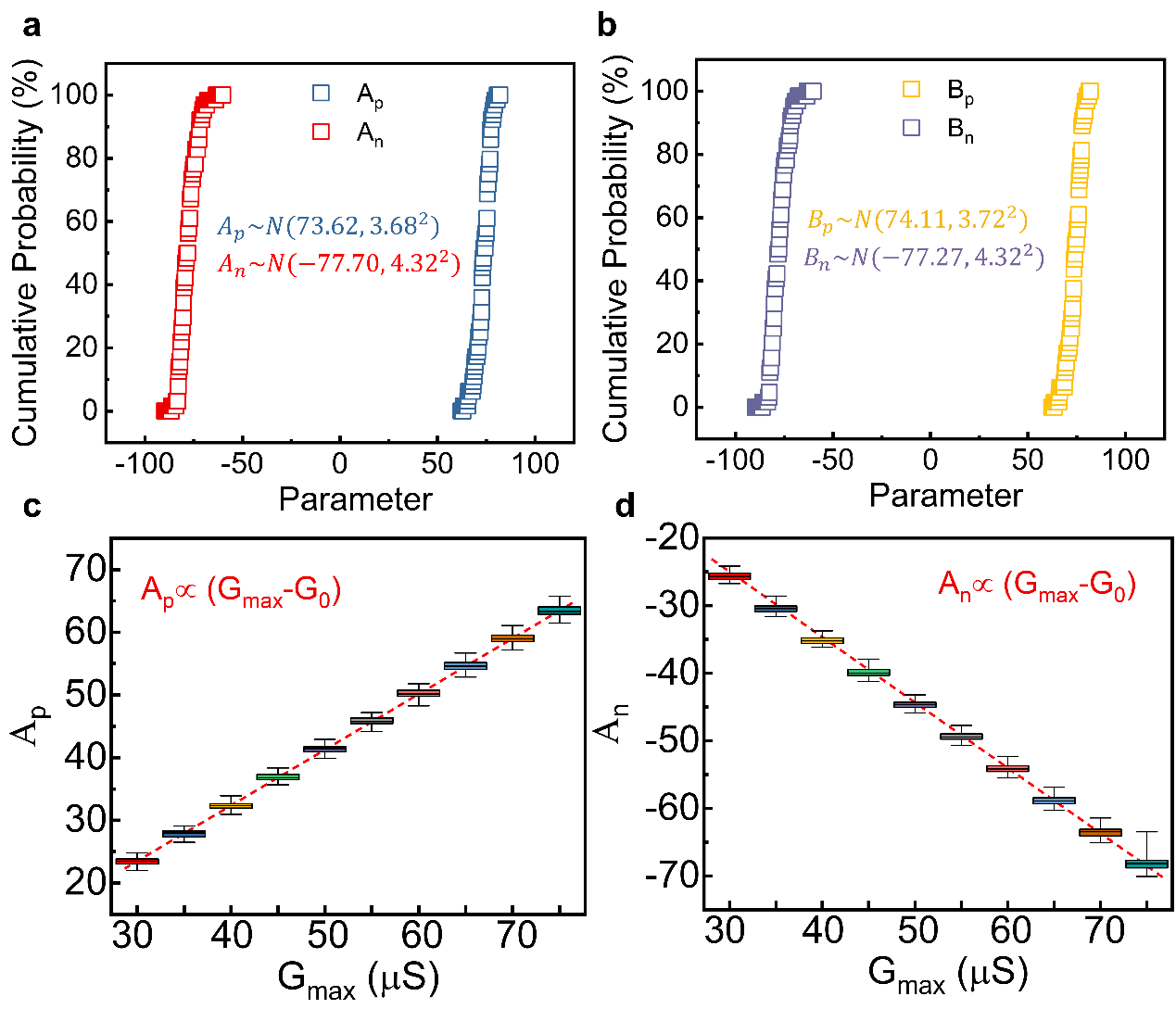


**Figure S18.** (a, b) Statistics of fitting parameters for weight transfer functions of the MTT unit in different devices, and the corresponding normal distribution fitting parameters are also attached to the figure. (c, d) Weight transfer parameters as a function of the maximum operation range for the hidden analog memory device.


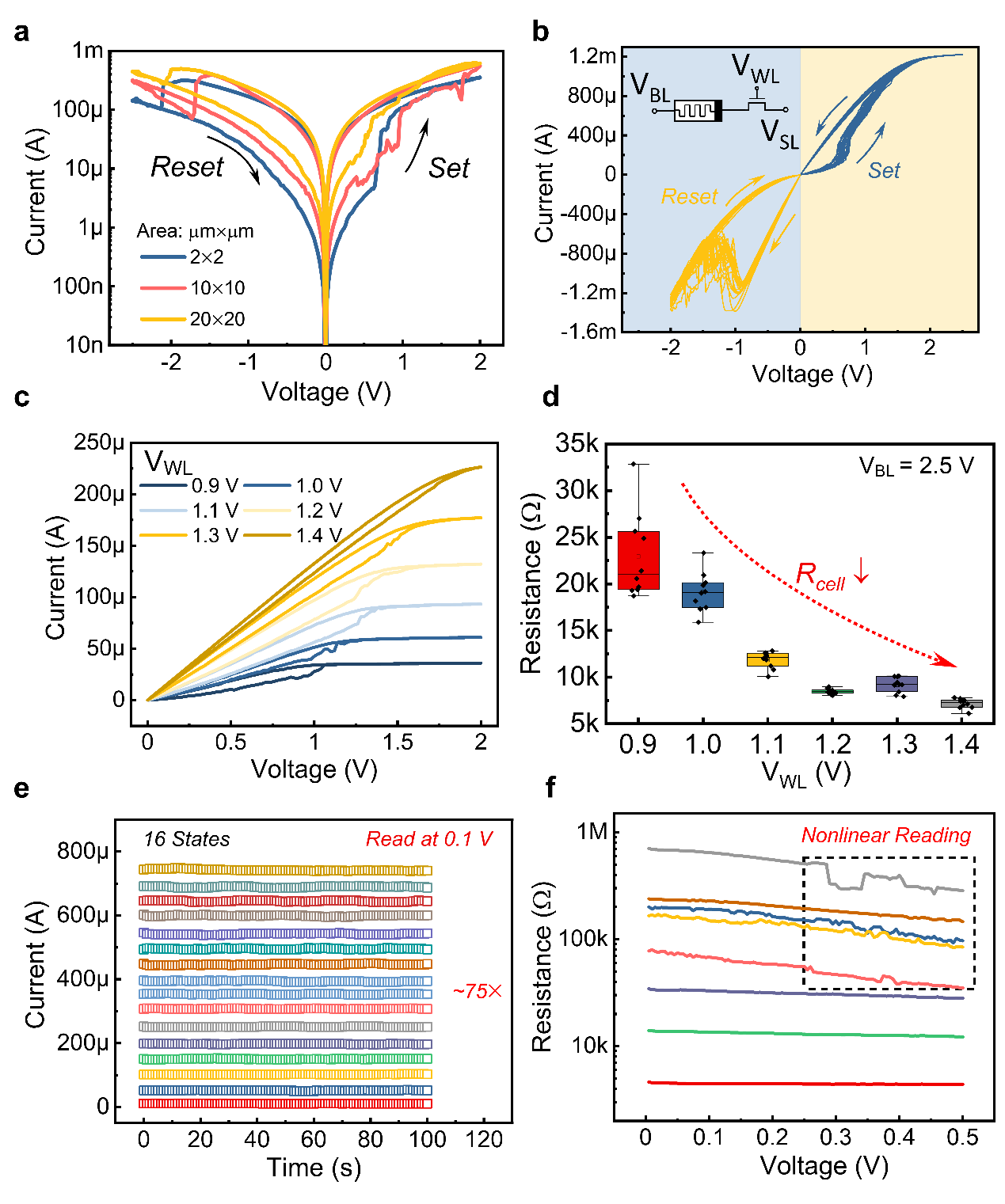


**Figure S19. Basic resistive switching characterization of RRAM device.** (a) DC switching characteristics of the RRAM device in different sizes, which mainly have an impact on the high resistance states due to the filamentary switching mechanisms. (b) The typical resistive switching characteristics of the 1T1R unit, which is repeated for 25 cycles, and V_WL_ is set as 2.5 V in the Set region. (c) Gradual set behavior of the 1T1R unit, in which the different states can be well controlled by the V_WL_. (d) The distribution of the final device states under the different V_WL_, in which it will be tighter under the larger V_WL_. (e) Retention of 16 distinguished states for 100 s under the 0.1 V reading voltage, in which the maximum ratio between different resistance states is about 75. (f) Nonlinear reading behaviors during the larger read voltage, which will result in the disturbance of some device states, especially high resistance states.


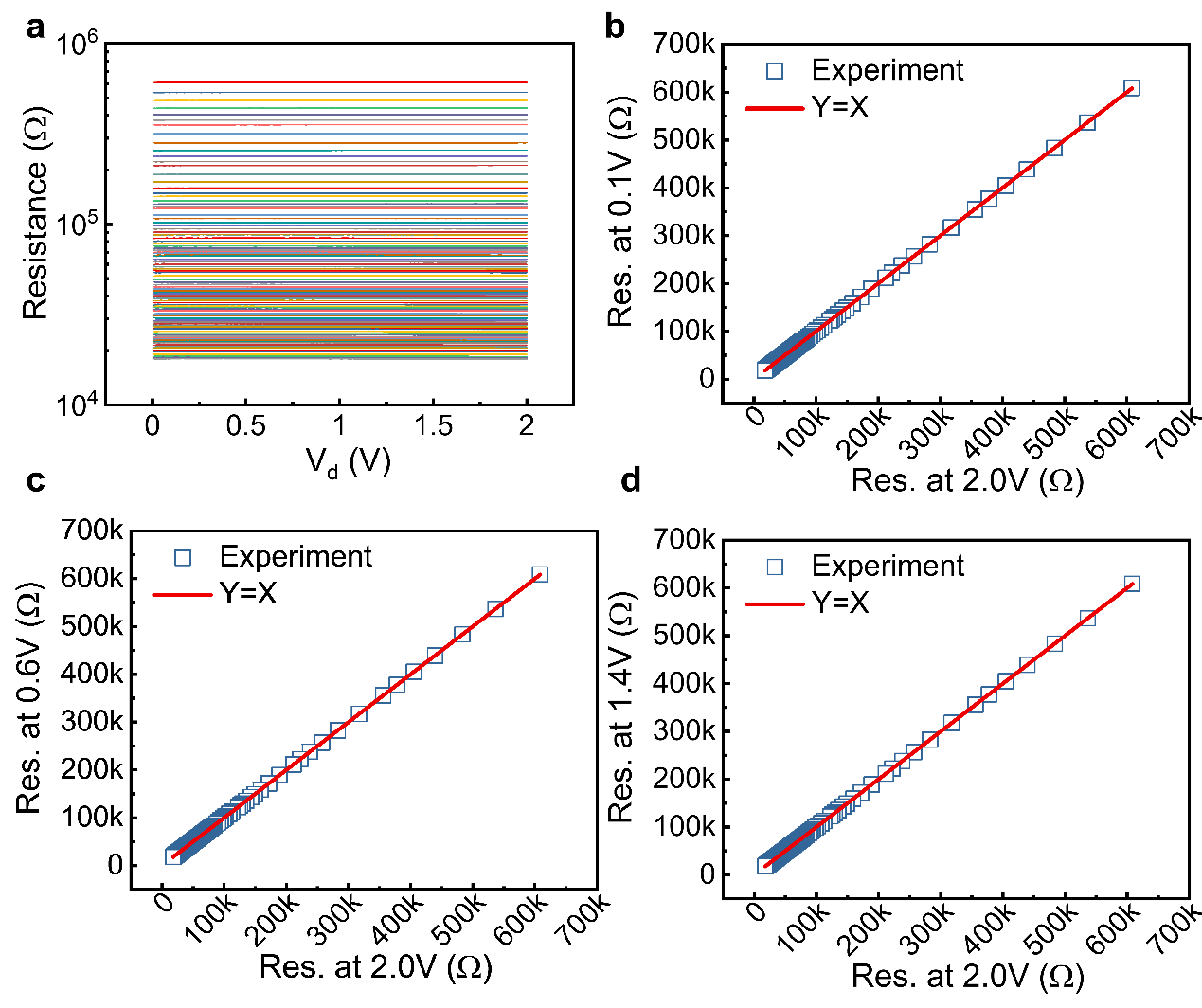


**Figure S20. Linear reading under large channel reading voltages of the ECRAM.** (a) ECRAM channel resistance as a function of drain-source reading voltage ranging from 0 V to 2 V, 98 different states are included in the total. (b-d) The relationship between the resistance read at medium voltages and 2.0 V, in which the medium voltages comprise 0.1 V, 0.6 V and 1.4 V in turn.


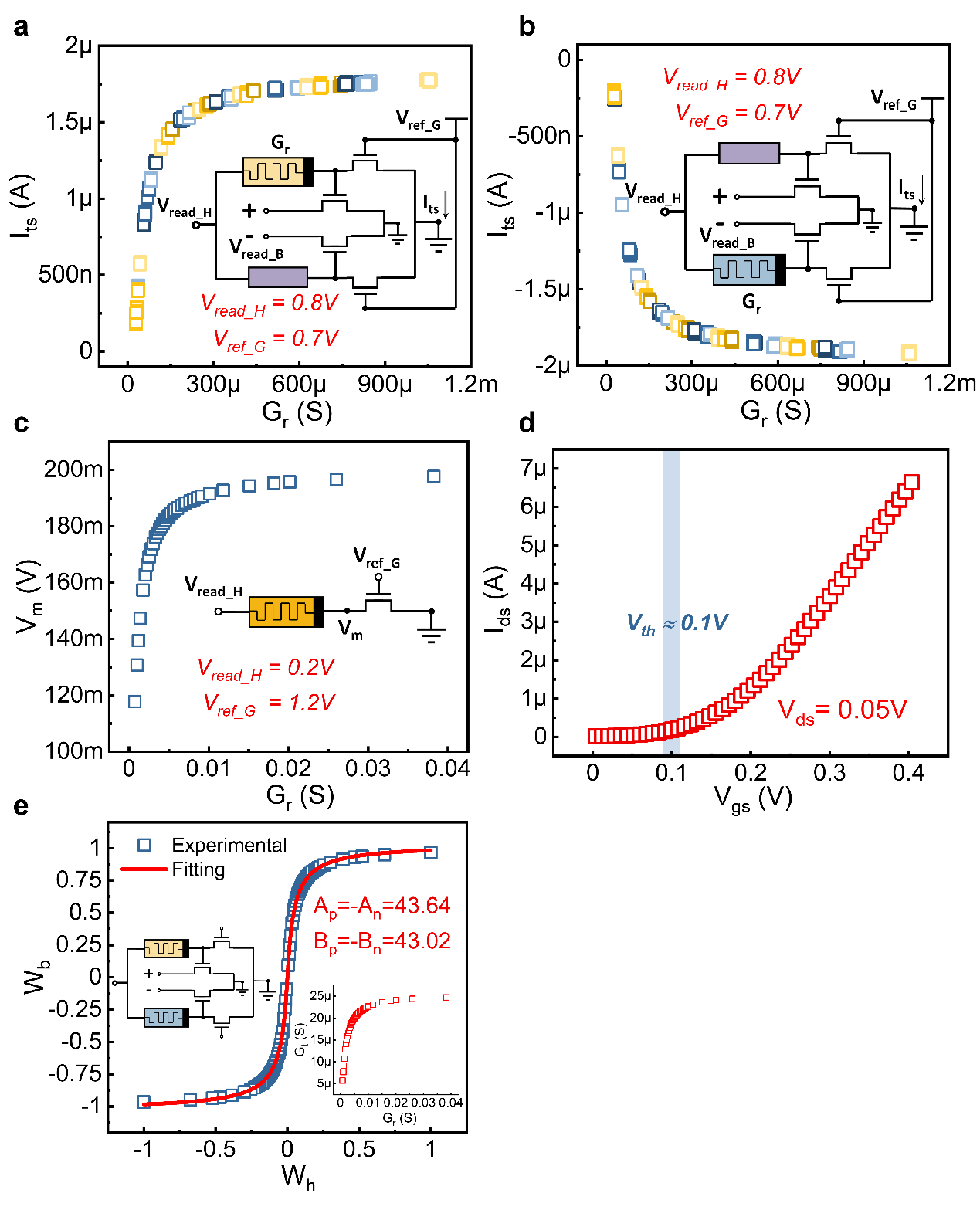


**Figure S21. Weight transfer functions of the RRAM-based MTT unit.** (a, b) The relationship between RRAM conductance and the channel current of the inference transistor under positive and negative bias conditions, respectively. The hidden weight bias voltage is set at 0.8 V, with the gate voltage of the reference transistor set at 0.7 V, while the positive and negative read voltages at the drain of the inferred transistor are set as 0.01 V and -0.01 V, respectively. (c) Measured node voltage V_m_, which is located between RRAM and reference transistor shown in the inset, as a function of RRAM conductance, in which the hidden weight bias voltage is 0.2 V and the gate voltage of the reference transistor is 1.2 V. (d) Transfer characteristic of the low threshold voltage transistor, in which V_th_ is about 0.1 V. (e) Simulated weight transfer function between analog hidden weights and binary inference weights, the inset is the simulated relationship between actual RRAM conductance and inference transistor conductance, which is implemented in more advanced CMOS platform.


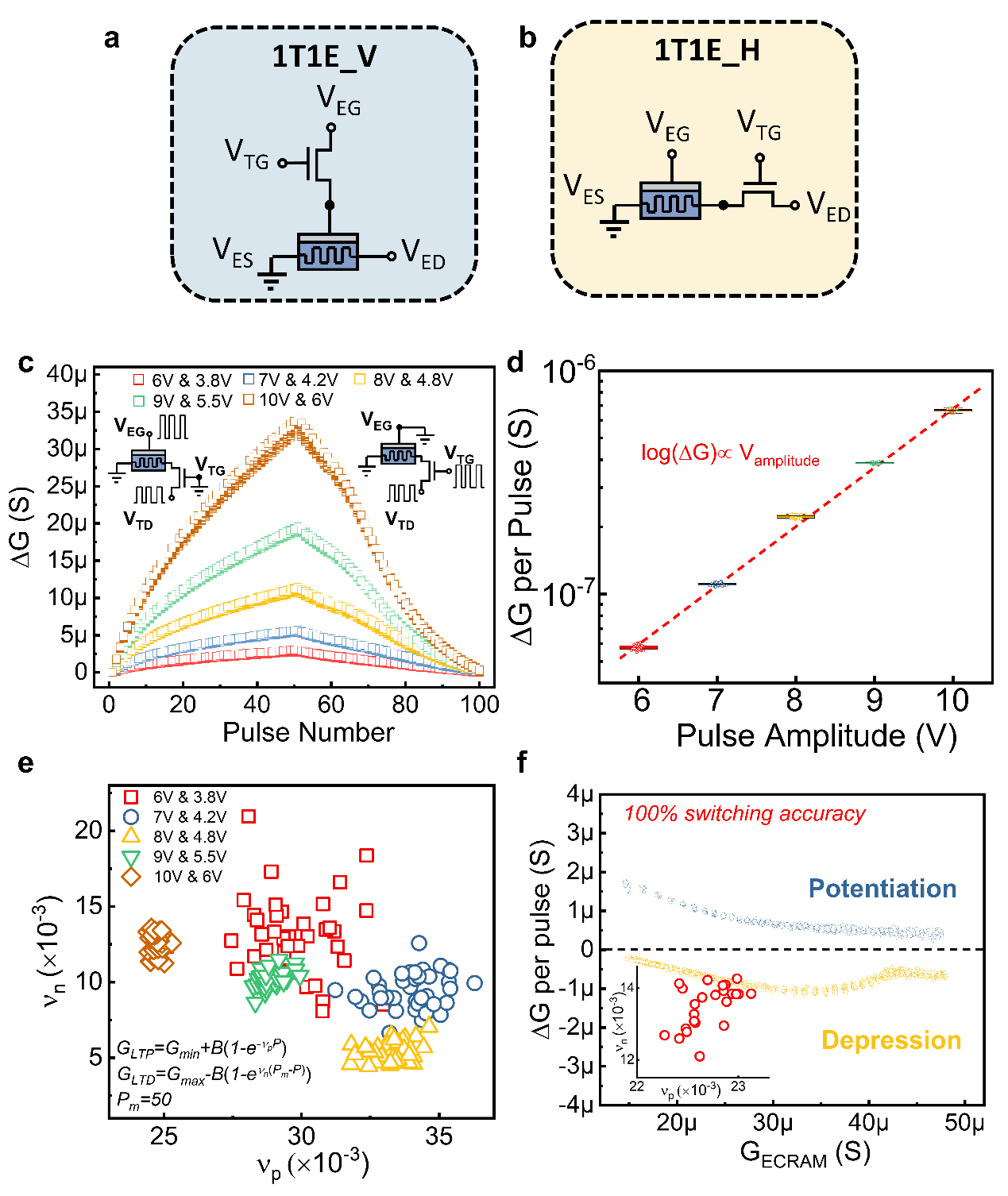


**Figure S22. Selective programming of the horizontal 1T1E unit.** (a, b) Schematic of conventional vertical 1T1E unit and newly introduced horizontal 1T1E unit, respectively. (c) epeated writing and erasing based on the two-terminal programming method, which is schematically reflected in the inset, and the conductance updating range can be well controlled by the amplitude of programming pulses. (d) Conductance change during each pulse as a function of programming pulse amplitude in the (c), which can be well fitted by the exponential function. (e) Linearity fitting parameters of potentiation and depression programming of different pulse amplitudes. (f) Statistics of conductance variation for each programming pulse during potentiation and depression, whose V_EG_ pulse and V_TG_ pulse during programming are 10 V/5 ms and 6 V/5 ms, respectively, the inset is the linearity fitting parameters of the conductance updating.


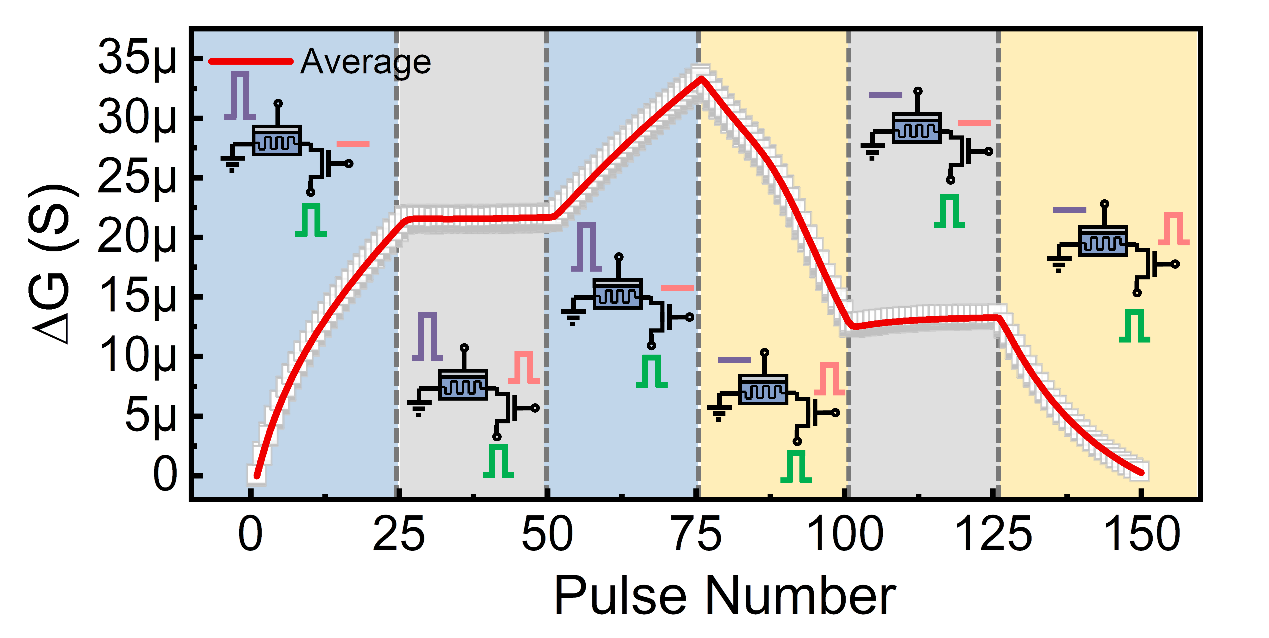


**Figure S23. Continual selective programming of the horizontal 1T1E unit, including three different modes which are potentiation, remaining and depression, corresponding pulse application schematics are also shown in the inset of the figure.**


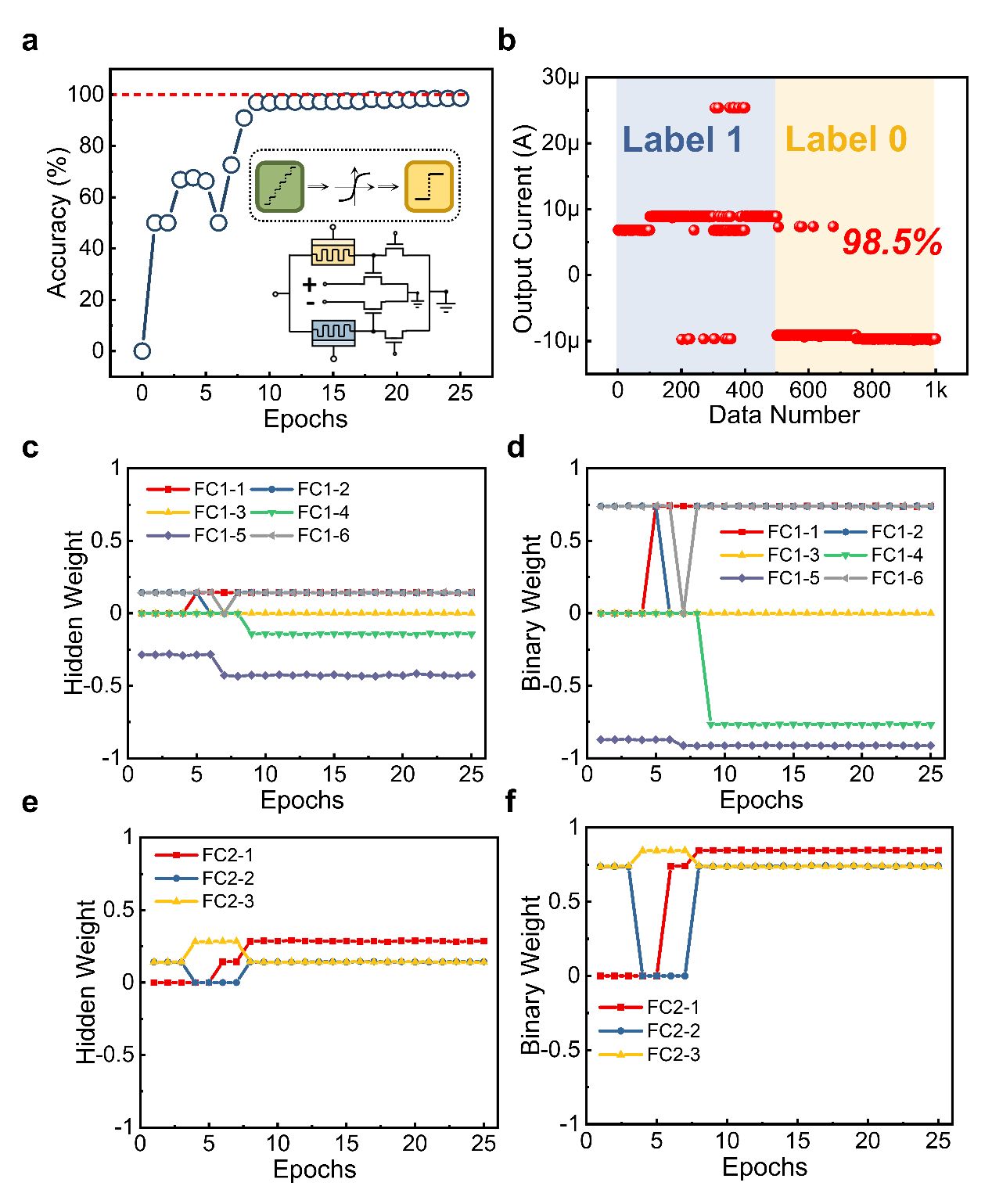


**Figure S24.** (a) Training performance of the XOR datasets with different initialization conditions. (b) Inference results mapped to hardware from the final trained weights, achieving a practical inference accuracy of 98.5%. (c-f) Hidden-weight and Binary-weight variation of all layers during network training.


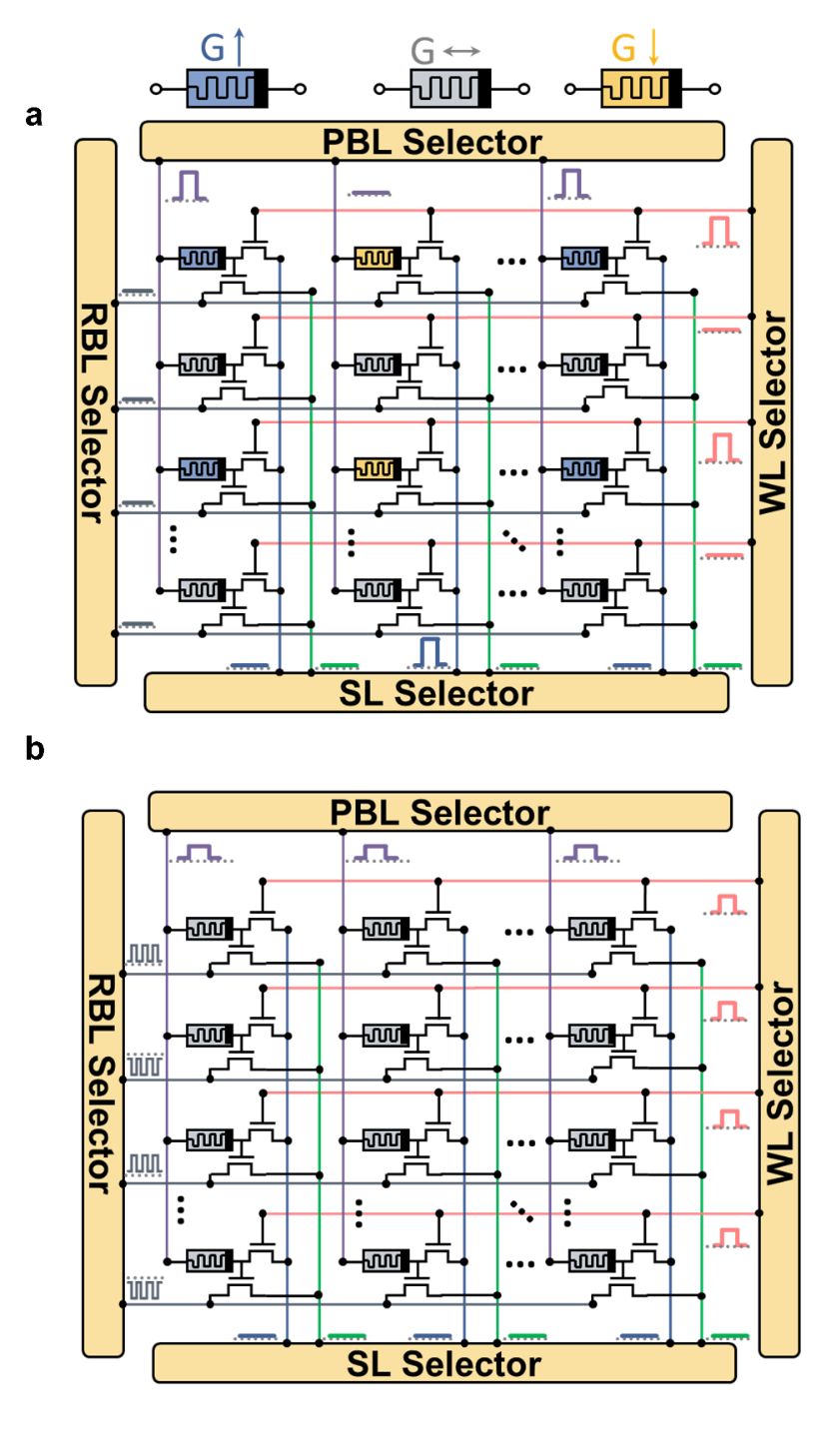


**Figure S25. Programming and inference of RRAM-MTT-based arrays.** (a) Schematic of array-level programming, where programming pulse signals at different ports are labeled alongside the respective signal lines. The blue, gray, and yellow blocks represent increases, maintenance, and decreases in the conductance of the RRAM after stimulus, respectively. (b) Schematic of inference based on the RRAM-MTT-based array, where the inference input waveform is applied at the drain terminal of the inference transistor controlled by the voltage division in the MTT unit.


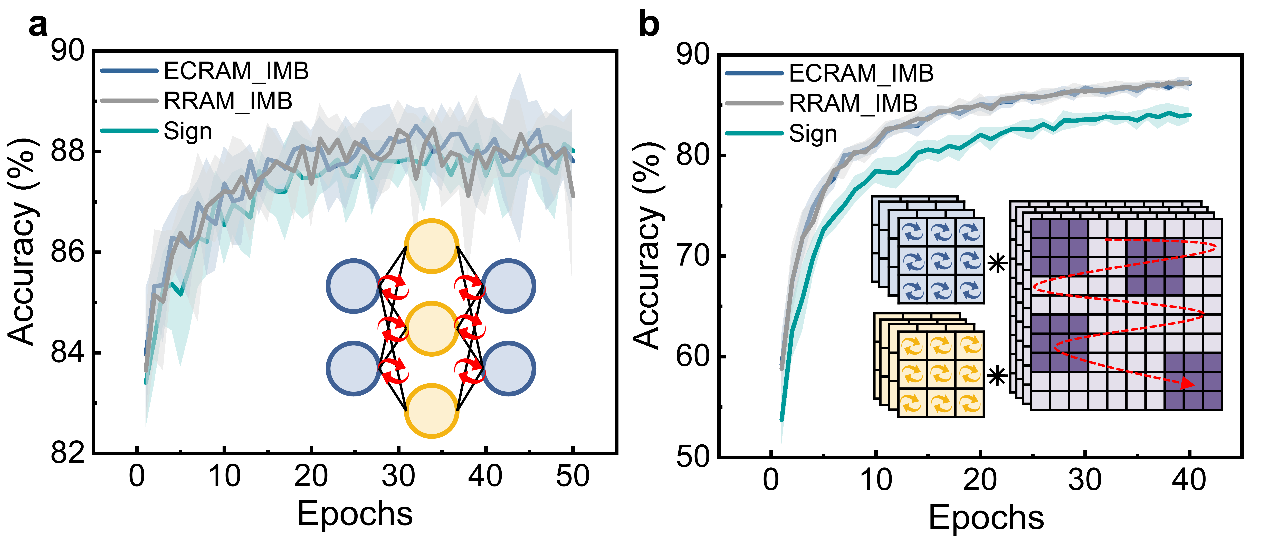


**Figure S26. Correlation of neural network performances when adopting different weight transfer functions, including ECRAM-based IMB function, RRAM-based IMB function and Sign-based transfer function, which is tested in both fully connected neural network (a) and convolutional neural network (b).**


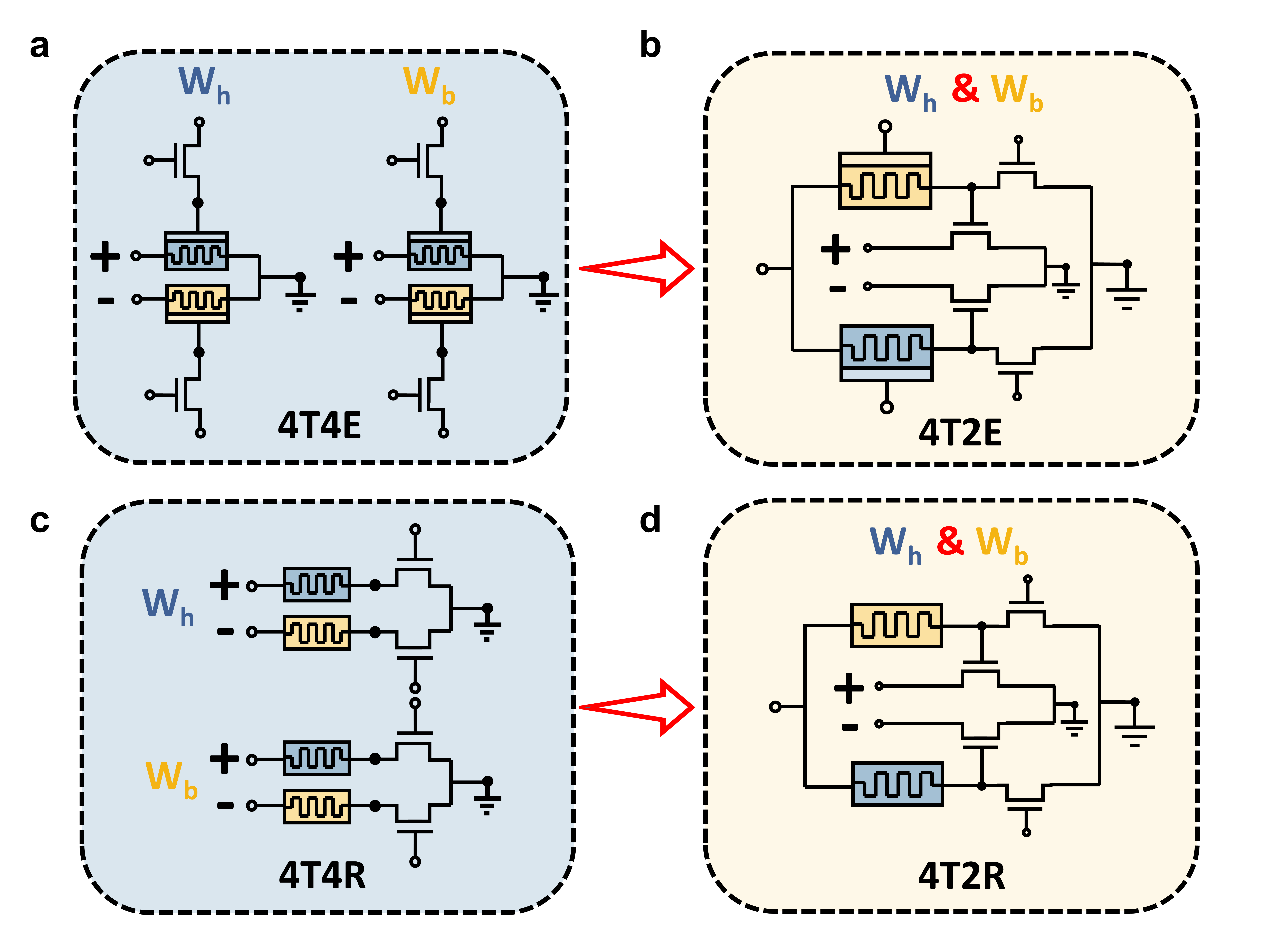


**Figure S27. Comparison of hardware overhead in the conventional CIM arrays and MTT-unit-based arrays, in which ECRAM and RRAM situations are both considered.**


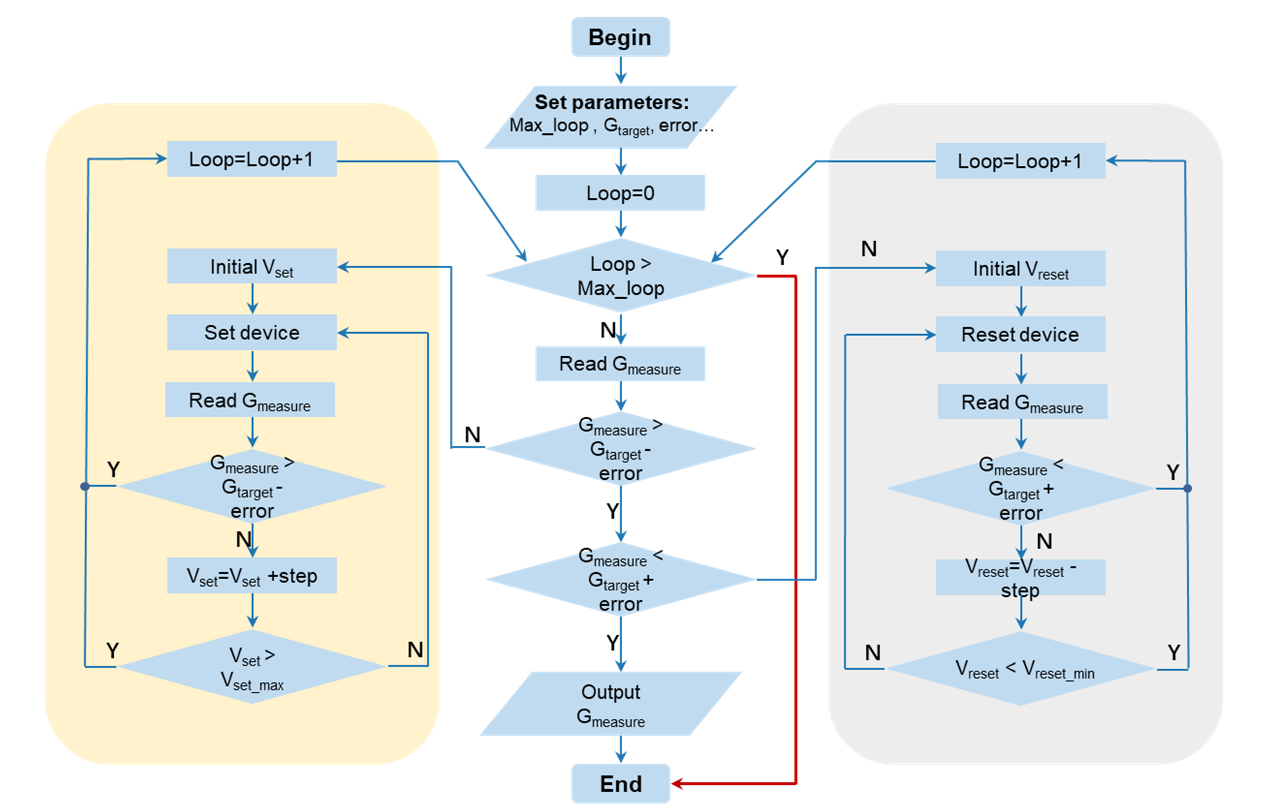


**Figure S28.** **Flowchart of the write-verify programming algorithm.** It mainly includes two modules, SET and RESET, both work in negative feedback principle and will continuously increase the intensity of stimulation if device states remain unchanged.


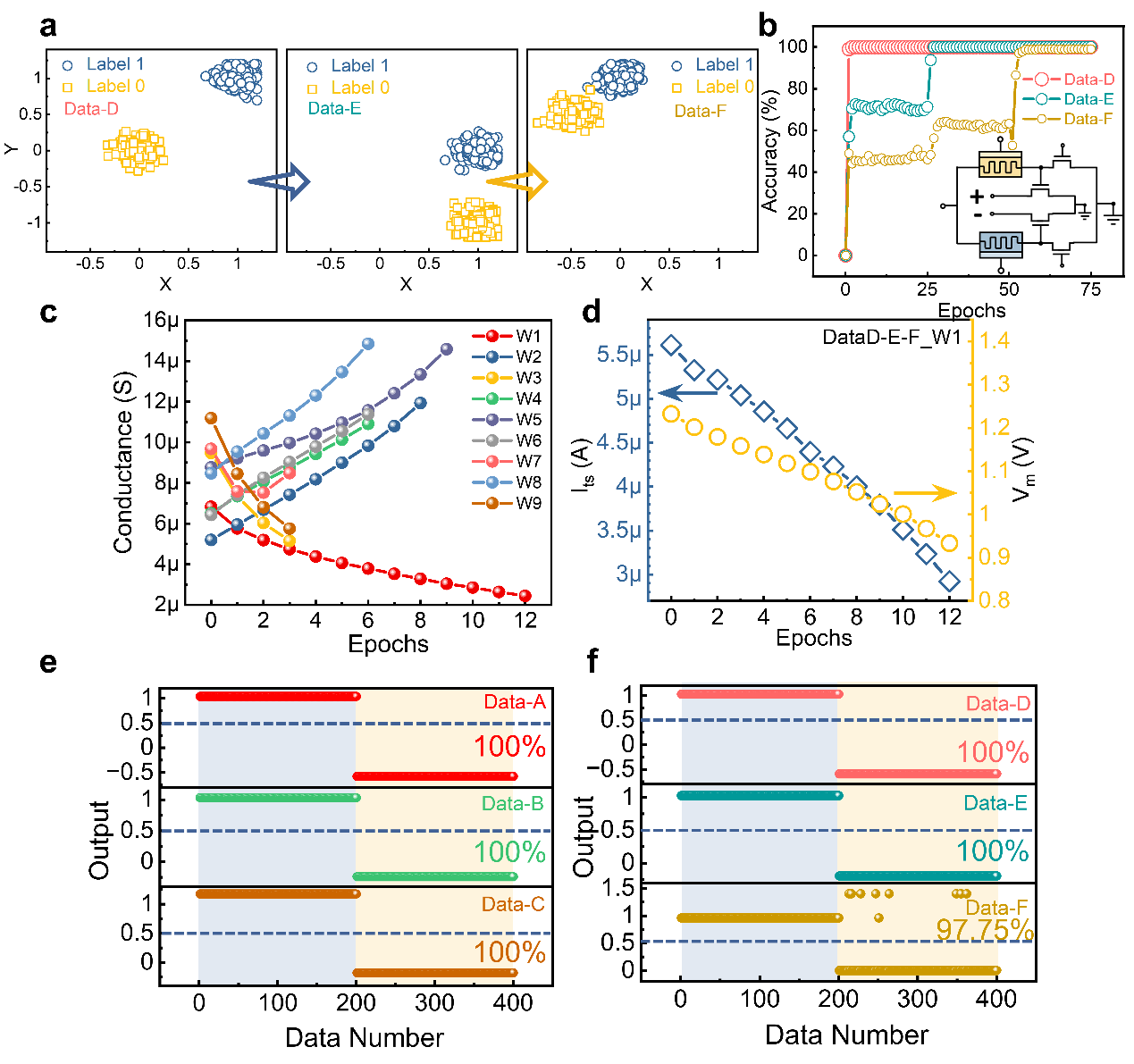


**Figure S29.** (a) Datasets used to test the continual learning performance of small networks, comprising three distinct datasets: Data-D, Data-E, and Data-F. (b) Training performance of the BNN based on metaplasticity on the different datasets in panel (a), where hidden weights are quantized to 3 bits during training, and the binary quantization curve utilizes weight transfer relationships from ECRAM-based MTT units shown in the inset. (c) The variation of hidden weight conductance during the write-verify programming process. (d) The read current of an inference transistor for a typical weight, along with the variation of the intermediate node voltage during the programming process. (e, f) Actual inference results when taking the programmed weights into neural networks, which include Data-A-B-C and Data-D-E-F.


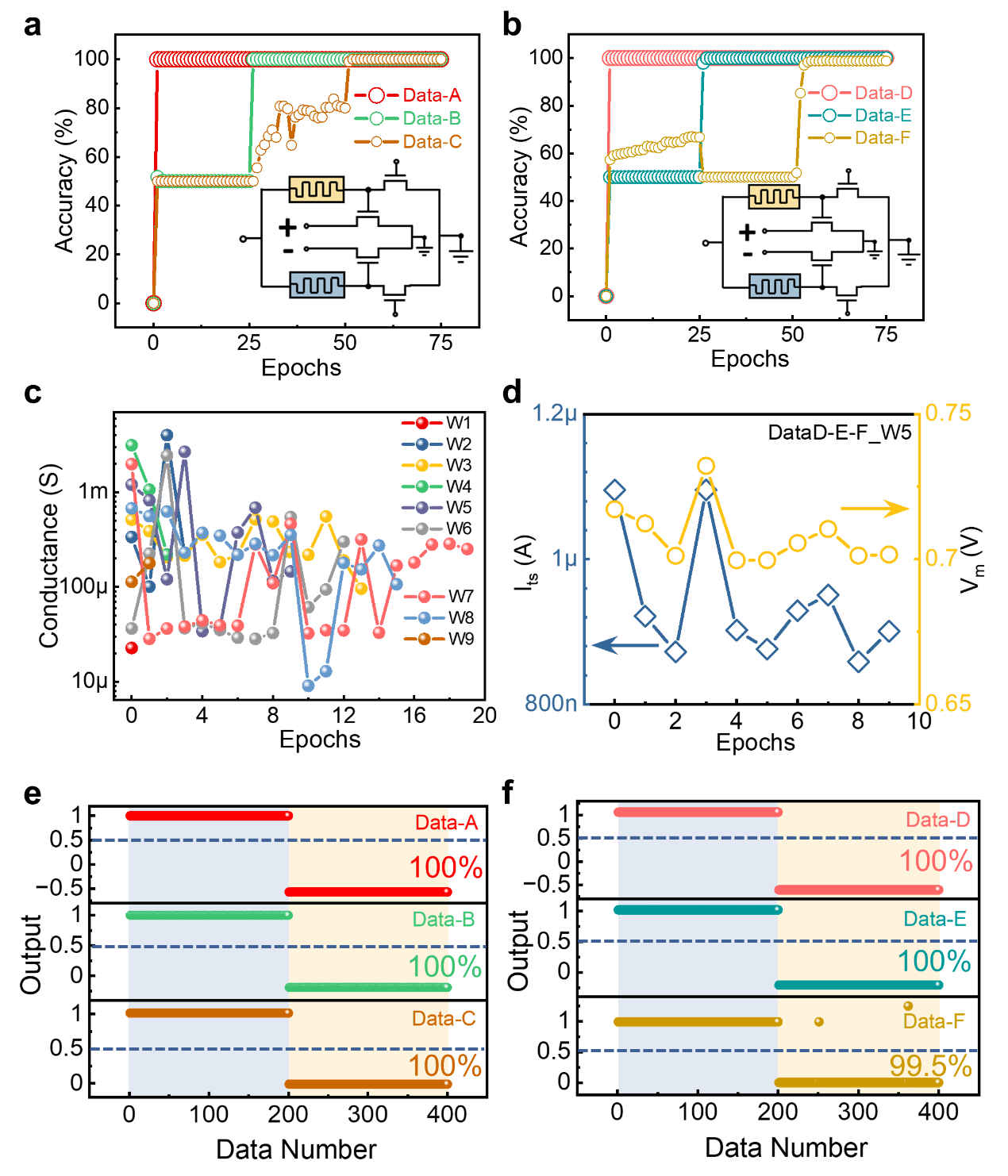


**Figure S30. Applications of weight transfer relationships in RRAM-based MTT units for continual learning.** (a, b) Neural network training for Data-A-B-C and Data-D-E-F, respectively, in which weight transfer function in RRAM-based MTT units is used. (c) The variation of hidden weight conductance during the write-verify programming process. (d) The read current of an inference transistor for a typical weight, along with the variation of the intermediate node voltage during the programming process. (e, f) Actual inference results when taking the programmed weights into the neural network, which include Data-A-B-C and Data-D-E-F, all the weight transfer functions are based on RRAM-MTT units.


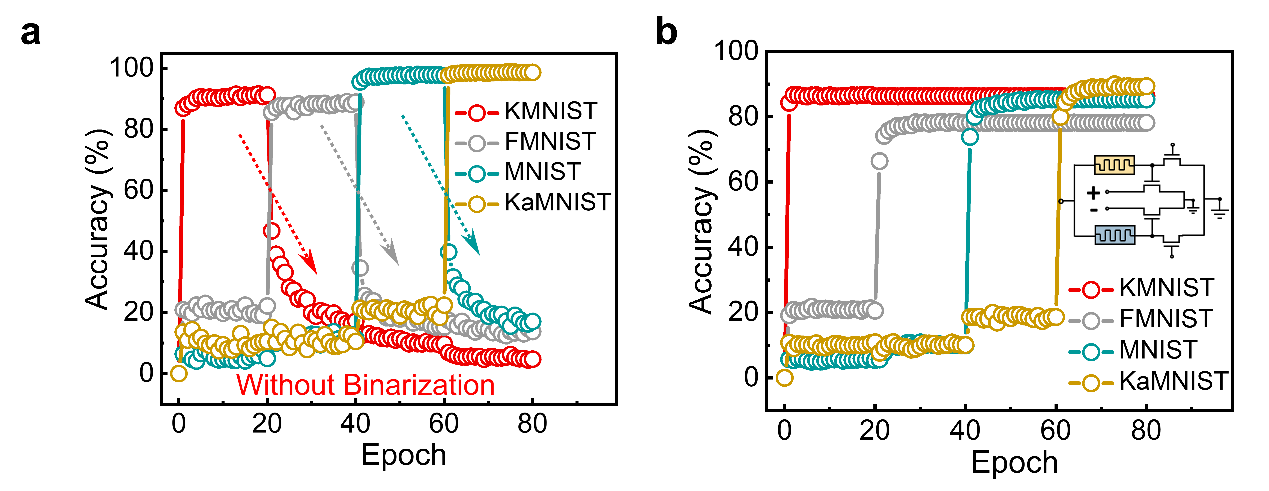


**Figure S31.** (a) Catastrophic forgetting in continual learning when the network is trained without binarization. (b) Training results of continual learning for KMNIST-FMNIST-MNIST-KaMNIST, employing a weight binary quantization function based on RRAM MTT units shown in the inset.


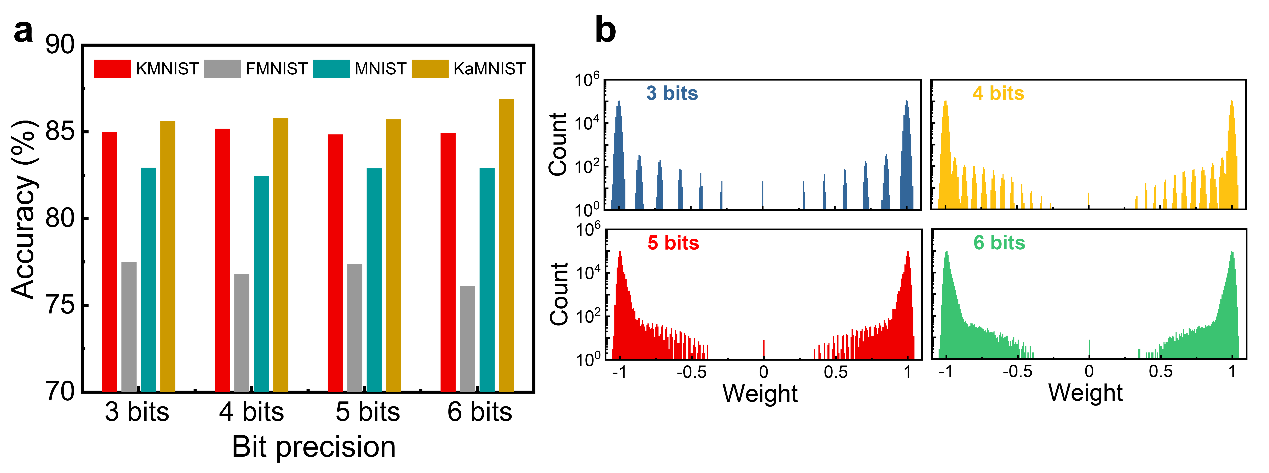


**Figure S32.** (a) Final testing accuracies of all the trained tasks concerning the quantized bit precision, ranging from 3 bits to 6 bits. (b) The trained weight distribution of the first layer of the neural network under the different quantized bit precisions.


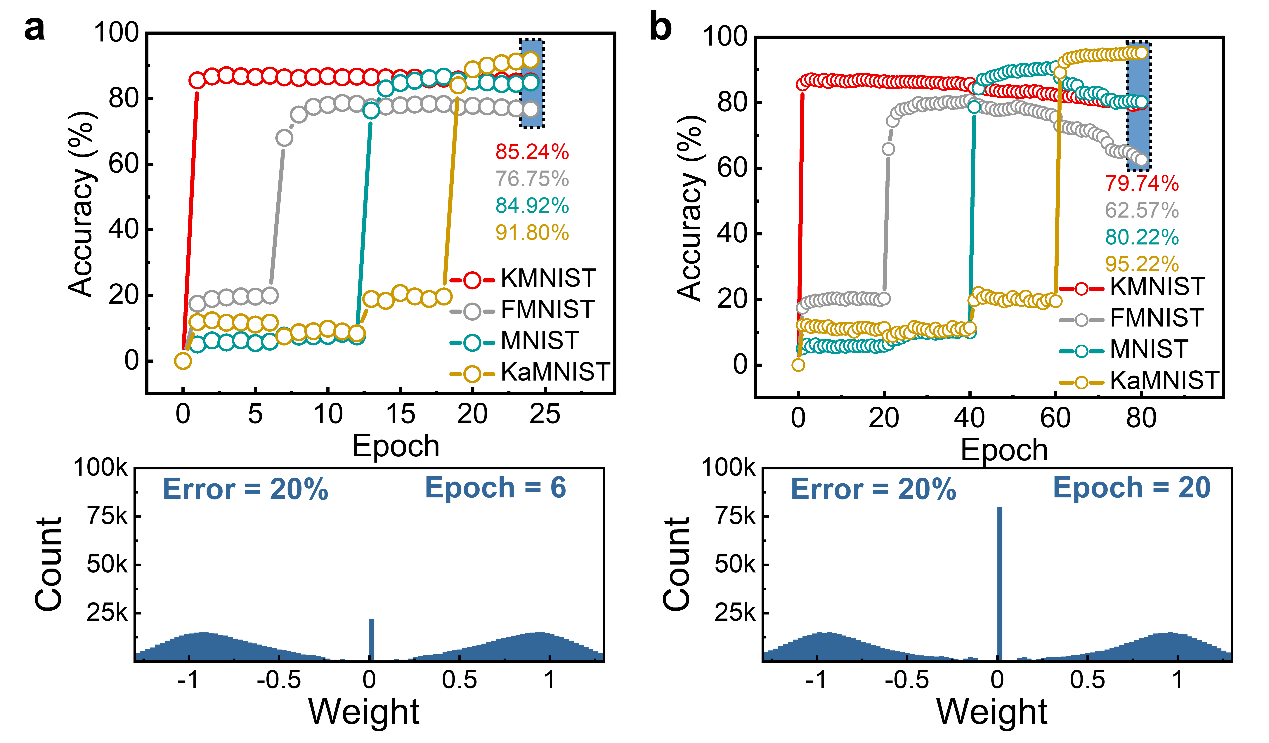


**Figure S33. The impacts on the continual learning capacity under the different training epochs and the given programming errors.** (a, b) Neural network training performances at the training epoch for each task of 6 and 20, respectively, and below is the corresponding final weight distribution of the first layer, both programming relative errors are set as 20%.


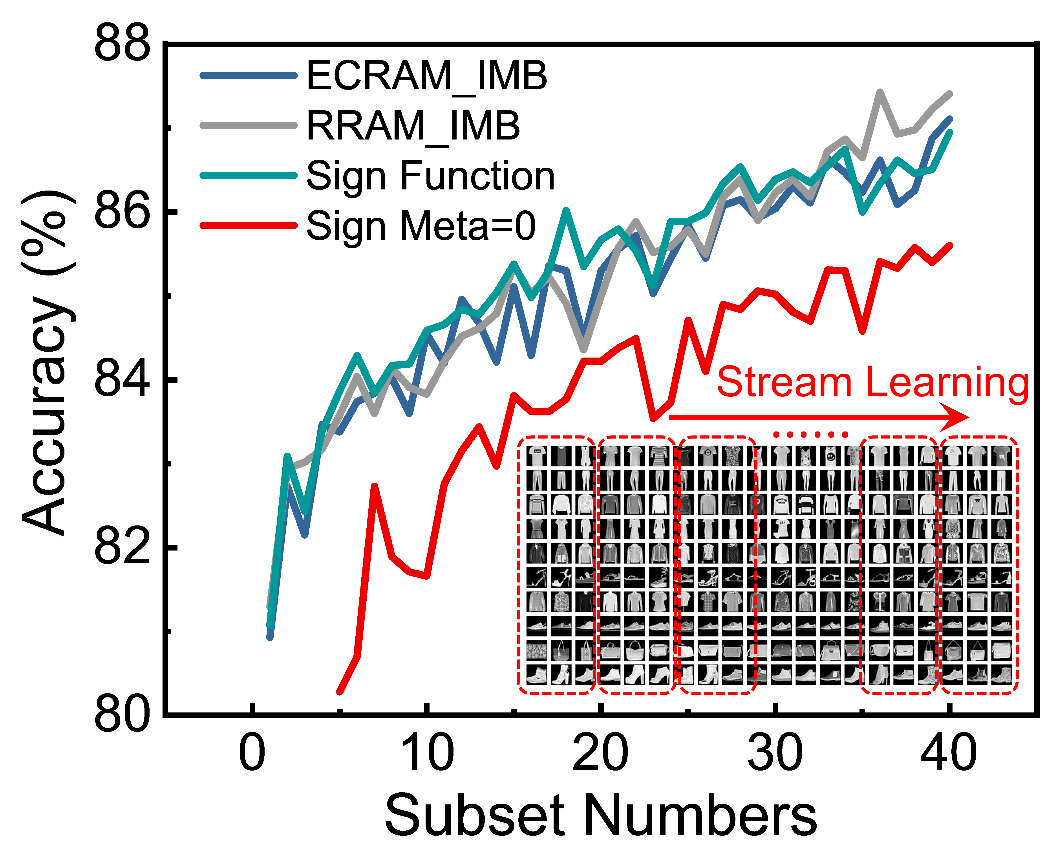


**Figure S34. Stream learning for the Fashion-MNIST, in which the weight transfer is based on ECRAM-MTT unit, RRAM-MTT unit, Sign Function, also the metaplasticity in the learning is also explored.**


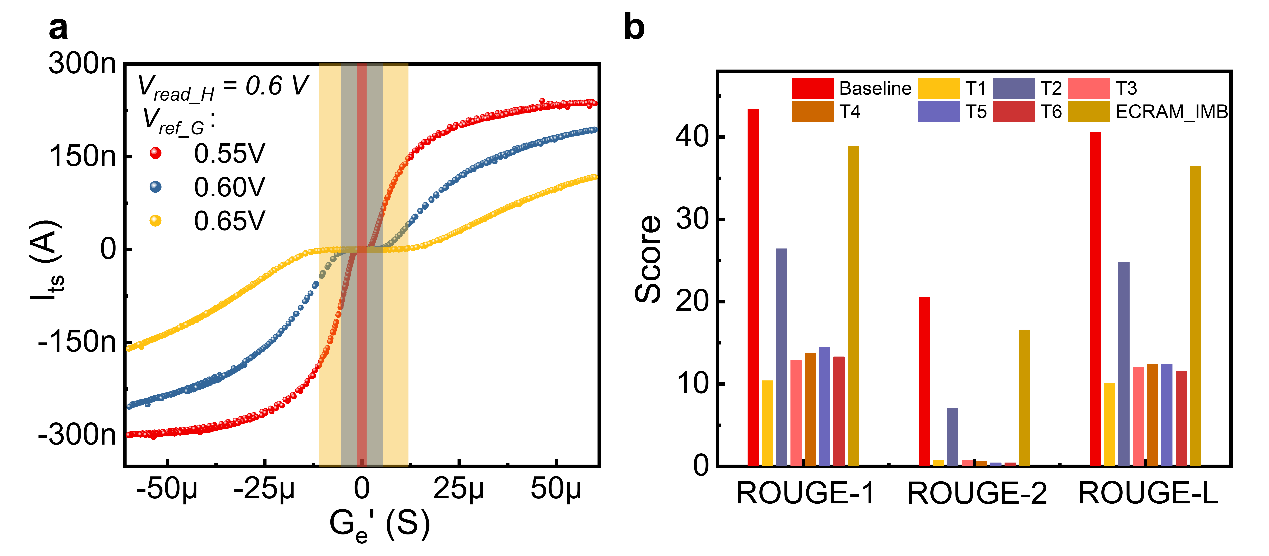


**Figure S35.** (a) Weight ternary quantization relationships based on MTT units, where the hidden weight bias voltage is set at 0.6 V, and the gate bias voltages of the reference transistor are set at 0.55 V, 0.60 V, and 0.65 V, respectively. (b) LLM learning results of the weight conversion relationships of MTT units under different bias conditions, where both the model input and activation functions are in 8-bit float format.

**Supplementary Note 1:** **The benchmark of energy efficiency in different hardware** **architecture**

To compare the energy efficiency in weight quantization of low-precision neural network hardware implementations, four different kinds of hardware architectures are evaluated on the analog hidden weight programming, weight binarization and one complete inference on the binary weights. It should be noted that the gradient computation is not included given that it is almost the same among the different hardware implementation methods, thus we mainly focus on one complete round of weight quantization and inference. Specifically, two kinds of hardware architecture include:

1. H_1_: Fully digital hardware implementation, in which both analog hidden weights and binary inference weights are stored in digital systems.
2. H_2_: MTT-array implementation, in which the MTT unit is responsible for storing both analog hidden weights and binary inference weights, including RRAM-MTT and ECRAM-MTT units.

During the whole weight quantization and inference process, energy consumption

and latency can be divided into 7 stages, which successively include:

1. E_1_: Power consumption for programming analog weights into memory arrays. (Data Programming)
2. E_2_: Power consumption for taking analog weight values out of digital memory arrays into binary computing in the processor. (Data Access)
3. E_3_: Power consumption for doing binary computation in processor. (Data Computation)
4. E_4_: Power consumption for accessing data after binary computing into digital memory. (Data Access)
5. E_5_: Power consumption for accessing binary weight values from digital memory. (Data Access)
6. E_6_: Power consumption for programming binary weights into non-volatile memory arrays. (Data Programming)
7. E_7_: Power consumption for doing one round of inference based on the binary weights, which is different in conventional Von-Neumann architecture and CIM architecture. (Data Computation)

For hardware implementation of H_1_, all the above power consumption is included, whereas as for H_2_, due to the in-situ weight transfer after programming analog hidden weights, E_2_~E_6_ could be canceled out, which only involves E_1_ and E_7_. The estimations of energy efficiency for both ECRAM-MTT units and RRAM-MTT units are conducted. Within a certain array size range, power consumption is positively correlated to array size, the most uncontrollable factor is the energy cost during programming analog weights into memory arrays, which will be estimated based on the experimental results in this paper, whether for ECRAM or RRAM. In the specific energy estimation, for the digital hardware platform, the processor uses the data of the A100 GPU^1^, and the non-volatile memory uses the Samsung 980 PRO NVMe solid-state drive^2^, including storage simulation weights and binary weights.

**Supplementary Table 1. Parameters used to simulate the weight transfer functions in the 1E1T1R-based MTT unit.**

| **Parameters** | **Value** | **Unit** |
| --- | --- | --- |
| µ_eff_ | 100 | cm^2^/(V·s) |
| C_ox_ | 3.45🞩10^-5^ | F/cm^2^ |
| W | 1 | µm |
| L | 1 | µm |
| V_T_ | 0.7 | V |
| m | 1.1 | - |
| k | 1.38🞩10^-23^ | J/K |
| T | 300 | K |
| q | 1.6🞩10^-19^ | C |
| V_ds_ | 0.1 | V |
| bias | 2.26🞩10^-7^ | A |

**Supplementary Table 2. Summary of applied signals in each terminal of the horizontal 1E1T unit during different conductance updating processes.**

| **Terminal** | **ECRAM Conductance** | | | |
| --- | --- | --- | --- | --- |
|  | **Potentiation** | **Remaining** | **Depression** | **Remaining** |
| V_EG_ | V_P_ | V_P_ | Ground | Ground |
| V_TG_ | Ground | V_SS_ | V_SS_ | Ground |
| V_ED_ | V_D_ | | | |
| V_ES_ | Ground | | | |

**Supplementary Table 3. Final testing accuracies in Figure S27 when adopting different weight transfer functions.**

| **Model** | **Dataset** | **Quantization** | **Accuracy (%)** |
| --- | --- | --- | --- |
| MLP | Fashion-  MNIST | Sign | 88.01(±0.36) |
|  |  | ECRAM_IMB | 87.81(±1.04) |
|  |  | RRAM_IMB | 87.12(±1.72) |
| CNN | CIFAR-10 | Sign | 84.03(±0.72) |
|  |  | ECRAM_IMB | 87.11(±0.69) |
|  |  | RRAM_IMB | 87.25(±0.34) |

**Supplementary Table 4. Final testing accuracies of all the tasks under the different weight programming errors and different training epochs.**

| **Error** | **Epoch** | **KMNIST** | **FMNIST** | **MNIST** | **KaMNIST** |
| --- | --- | --- | --- | --- | --- |
| 5% | 6 | 86.74% | 77.05% | 84.14% | 87.94% |
|  | 20 | 87.09% | 78.34% | 84.50% | 89.04% |
| 10% | 6 | 86.61% | 77.37% | 84.22% | 87.92% |
|  | 20 | 85.82% | 77.90% | 84.56% | 90.58% |
| 15% | 6 | 86.13% | 78.27% | 83.31% | 89.25% |
|  | 20 | 82.15% | 71.87% | 83.46% | 93.96% |
| 20% | 6 | 85.24% | 76.75% | 84.92% | 91.80% |
|  | 20 | 79.74% | 62.57% | 80.22% | 95.22% |
| 30% | 6 | 57.58% | 65.32% | 83.48% | 94.95% |
|  | 20 | 32.37% | 38.18% | 68.72% | 96.47% |

**Supplementary Table 5. Fitting parameters of the ternary weight transfer function in different bias conditions and corresponding notes are also demonstrated.**

| **V_read_H_ (V)** | **V_ref_G_ (V)** | **Note** | **r** | **A_p_** | **B_p_** | **A_n_** | **B_n_** |
| --- | --- | --- | --- | --- | --- | --- | --- |
| 0.7 | 0.55 | T1 | 0.02 | 18.66 | 18.38 | -23.21 | -21.45 |
|  | 0.60 | T2 | 0.06 | 6.00 | 5.69 | -7.28 | -6.40 |
|  | 0.65 | T3 | 0.14 | 1.43 | 0.99 | -1.73 | -1.10 |
| 0.6 | 0.55 | T4 | 0.03 | 6.96 | 10.56 | -8.52 | -12.23 |
|  | 0.60 | T5 | 0.07 | 1.71 | 2.44 | -2.22 | -3.01 |
|  | 0.65 | T6 | 0.20 | 0.22 | 0.15 | -0.37 | -0.33 |

**Reference**

1. https://www.nvidia.com/en-us/data-center/a100
2. https://www.samsung.com.cn/memory-storage/nvme-ssd/980-pro-nvme-m-2-1tb-mz-v8p1t0bw
